# Evidence from a broad-range PNA probe links several *Prevotella* species to bacterial vaginosis

**DOI:** 10.7717/peerj.20902

**Published:** 2026-03-26

**Authors:** Taynara Mulinde, Lúcia G. V. Sousa, Joana Castro, Sheridan D. George, Christina A. Muzny, Nuno Cerca

**Affiliations:** 1 Centre of Biological Engineering, University of Minho, Braga, Portugal; 2 INIAV—National Institute for Agrarian and Veterinary Research, Vila do Conde, Portugal; 3 Division of Infectious Diseases, University of Alabama—Birmingham, Birmingham, AL, United States of America; 4 LABBELS—Associate Laboratory, Braga, Portugal

**Keywords:** *Prevotella* spp, *P. bivia*, Novel molecular probe, PNA FISH, Bacterial vaginosis

## Abstract

**Background:**

Bacterial vaginosis (BV) is the most prevalent vaginal infection among reproductive-age women. It is associated with multiple adverse health outcomes in women including adverse pregnancy outcomes, an increased risk of pelvic inflammatory disease, infertility, and an increased risk of HIV and other sexually transmitted infections. BV is characterized by an imbalance in the vaginal microbiota, namely a decrease in protective *Lactobacillus* species and an overgrowth of facultative and strict anaerobic bacteria, leading to the development of a polymicrobial biofilm. Despite extensive research, the etiology of BV remains unclear, and its pathophysiology is not fully understood. It has been hypothesized that *P. bivia*, in combination with *Gardnerella* spp., plays an important role in the early development of the BV biofilm. We previously developed a peptide nucleic acid (PNA) probe specifically targeting *P. bivia* to investigate its role as a potential early colonizer. However, our recent findings have raised doubts about the specificity of this association, suggesting a broader involvement of other *Prevotella* species in incident BV (iBV).

**Methods:**

A new PNA probe targeting *Prevotella* spp. 23S rRNA was developed compared to the existing *P. bivia*-specific probe. This new probe was optimized *in vitro* through a variation of hybridization temperatures and times. Its performance was evaluated using a collection of 28 *Prevotella* strains representing 24 different species and 38 non-*Prevotella* spp. typically found in BV in order to assess its sensitivity and specificity. Both probes were tested on vaginal swab specimens from women with and without BV to assess the bacterial count and detection of *Prevotella* species.

**Results:**

*In vitro* validation demonstrated that the new *Prevotella* spp. probe achieved a specificity of 100% and sensitivity of 96%. As expected, its broader detection allowed identification of a wider range of *Prevotella* spp. compared to the *P. bivia*-specific probe, which was intentionally restricted to a single species. Application to clinical specimens revealed that the new probe identified a significantly higher count of *Prevotella* spp. in 6/9 (66.6%) BV-positive specimens compared to the *P. bivia*-specific probe. In 2/9 (22.2%) healthy control specimens, greater *Prevotella* spp. detection was also observed.

**Conclusions:**

Our findings suggest that the involvement of *Prevotella* spp. in BV extends beyond *P. bivia*, implicating a wider range of species which could be present in the polymicrobial BV biofilm. The broader specificity of this new *Prevotella* spp. probe provides a valuable tool for future research on the vaginal microbiome and the pathogenesis of BV.

## Introduction

Bacterial vaginosis (BV) is the most prevalent vaginal infection among women of reproductive age, with significant global impact (Chen et al., 2021; Bradshaw et al., 2025). It is associated with an increased risk of multiple adverse health outcomes including adverse birth outcomes (Hendler et al., 2007), pelvic inflammatory disease (Wiesenfeld et al., 2002), infertility (Ravel, Moreno & Simón, 2021), and an increased risk of acquisition of HIV and other sexually transmitted infections (STIs) (Atashili et al., 2008). BV is characterized by a shift from a protective, *Lactobacillus* spp.-dominated vaginal microbiota to a dysbiotic environment, characterized by a polymicrobial biofilm enriched with anaerobic bacteria (Muzny et al., 2019). Prior research suggests that *P. bivia* may act as an early colonizer in incident BV (iBV) pathogenesis, as it is often found in association with other BV-associated bacteria (BVAB) including *Gardnerella* spp. and *Fannyhessea vaginae*, forming a complex biofilm that may tolerant to traditional treatments (Sousa et al., 2023; Muzny et al., 2018; Sousa, Pereira & Cerca, 2023).

The role of *Prevotella* spp. in BV extends beyond *P. bivia* with *P. amnii, P. disiens*, and *Hoylesella timonensis* (previously known as *P. timonensis*) also found in women with BV (Tett et al., 2021; George et al., 2024; Dillard et al., 2025). To investigate BV biofilm development, we used a multiplex Peptide Nucleic Acid (PNA) Fluorescence In Situ Hybridization (FISH) approach targeting *Gardnerella* spp. (Machado et al., 2013), *F. vaginae* (Sousa et al., 2021) and *P. bivia* (Sousa et al., 2023), in longitudinal vaginal samples from women who developed incident BV (iBV) compared to healthy non-case participants matched by age, race and contraceptive method (Muzny et al., 2024). Unexpectedly, *P. bivia* counts remained low and similar between groups (George et al., 2025), in contrast to prior studies (Zozaya-Hinchliffe et al., 2010; Gilbert et al., 2019) including our own (Muzny et al., 2018; Elnaggar et al., 2023), which implicated *P. bivia* in iBV pathogenesis. This discrepancy may come from 16S sequencing limitations in distinguishing *Prevotella* spp. (Chen et al., 2015). Thus, the role of other vaginal *Prevotella* spp. in iBV pathogenesis requires further research (Segui-perez et al., 2024). To address this, we developed a novel PNA probe targeting the *Prevotella* genus, based on 23S rRNA gene sequences, designed to detect common vaginal *Prevotella* spp. Its performance was evaluated *in vitro* and applied to vaginal swab specimens to assess its potential for genus-wide detection of *Prevotella* spp. in iBV.

## Materials & Methods

### *In silico* design of the *Prevotella* spp. PNA probe

A PNA probe specific to the detection of *Prevotella* spp. was developed using previously described protocols (Sousa et al., 2021; Sousa et al., 2023). Briefly, the sequences of the 16S and 23S rRNA genes from the four most common vaginal *Prevotella* spp. (*P. bivia*, *P. amnii*, *P. disiens*, and *P. timonensis*) were retrieved from the ARB-SILVA database (version 138.1; https://www.arb-silva.de/search/) using the following criteria: a minimum length of 1,200 bp for 16S rRNA and 1,600 bp for 23S rRNA, and a minimum quality score of 90. To evaluate probe specificity, 16S rRNA sequences from eight closely related bacterial species were retrieved from ARB-SILVA using the same selection criteria, namely *Hallella seregens*, *Paraprevotella clara*, *Paraprevotella xylaniphila*, *Bacteroides fragilis*, *Parabacteroides distasonis*, *Tannerella forsythia*, *Alloprevotella rava* and *Porphyromonas catuniae*. The sequences were aligned using Clustal Omega (version 1.2.2), which is implemented in Geneious Prime software (Biomatters, Auckland, New Zealand). Conserved regions of the *Prevotella* sequences were chosen as potential probes, based on perfect matches with sequences of interest and mismatches (preferably > 2) with sequences of non-*Prevotella* spp. Theoretical sensitivity and specificity of the PNA probes were calculated according to the procedures described by Almeida et al. (2013). The potential probes were evaluated using the TestProbe tool from Arb-Silva with no mismatches allowed. Sequences with the highest theoretical sensitivity and specificity, complementarity with a low number of non-interest sequences, GC content between 40% and 60%, high melting temperature (>50 °C), and Gibbs free energy ranging from-13 kcal/mol to -20 kcal/mol were selected (Yilmaz & Noguera, 2004; Almeida et al., 2011). The selected probe was then synthesized (Eurogentec, Seraing, Belgium) and the oligonucleotide N-terminus was linked to an Alexa Fluor molecule *via* a double 8-amino-3,6-dioxaoctanoic acid linker (*Prevotella* spp. probe: Alexa Fluor 488-OO- AGGCTCGCTTTCACT).

### Growth conditions and strains

The source of all *Prevotella* spp. used in this study is listed in Table S1. Strains were kept at −80 °C before each experiment. The strains were grown on Columbia Blood Agar Base plates (Oxoid, Basingstoke, UK) supplemented with 5% (v/v) of defibrinated horse blood (Oxoid). Most bacteria were grown at 37 °C and 10% CO_2_ for 48 h, with the exception of *Actinomyces urogenitalis, Aerococcus christensenii, Bifidobacterium bifidum, F. vaginae, Lactobacillus iners, Megasphaera micronuciformis, Mobiluncus curtisii, M. mulieris, Mycoplasma hominis, Peptostreptococcus anaerobius, Porphyromonas asaccharolytica, Prevotella* spp., *Cutibacterium acnes, Sneathia sanguinegens* and *Veillonella parvula*, which were grown at 37 °C under anaerobic conditions (AnaeroGen Atmosphere Generation system, Oxoid).

### PNA-FISH procedure

For PNA-FISH experiments, a bacterial suspension was prepared in phosphate-buffered saline (PBS) solution, with the optical density (OD) adjusted to 0.1 at 620 nm. A 2-fold dilution was prepared from this bacterial suspension. Afterward, 20 μL of the suspension was spread on epoxy coated microscope glass slides (Thermo Fisher Scientific, Lenexa, KS, USA) and left to dry at 37 °C for 1 h. Once the slides were dry, the fixation and permeabilization step was performed using 100% (v/v) methanol (Thermo Fisher Scientific) for 15 min, 4% (w/v) paraformaldehyde (Thermo Fisher Scientific) for 10 min, followed by 50% (v/v) ethanol (Thermo Fisher Scientific) for 15 min. The slides were then allowed to dry at room temperature. For the hybridization step, 10 μL of hybridization solution containing 10% (w/v) dextran sulfate (Sigma-Aldrich, Munich, Germany), 10 mM NaCl (Sigma-Aldrich), 30% (v/v) formamide (Thermo Fisher Scientific), 0.1% (w/v) sodium pyrophosphate (Thermo Fisher Scientific), 0.2% (w/v) polyvinylpyrrolidone (Sigma-Aldrich), 0.2% (w/v) Ficoll (Sigma Aldrich), five mM disodium EDTA (Panreac, Barcelona, Spain), 0.1% (v/v) Triton X-100 (Thermo Fisher Scientific), 50 mM Tris–HCl Microscopic Analysis (pH 7.5; Thermo Fisher Scientific), and 200 nM of the PNA probe was applied to the slides which were then covered with a coverslip. The slides were placed in wet paper inside of opaque containers and incubated at various selected temperatures and time intervals. Hybridization time and temperatures were optimized using the *P. bivia* ATCC 29303 strain, with evaluations conducted at temperatures ranging from 52 °C to 65 °C and durations between 60 and 90 min. The optimized temperature and incubation time (56 °C, 60 min) were used for sensitivity and specificity assays in all strains tested. After incubation, the slides were removed and placed into a wash solution containing five mM Tris-base (Thermo Fisher Scientific), 15 mM NaCl, 0,1% (v/v) Triton-X100 (Thermo Fisher Scientific), where they were incubated for 30 min at 56 °C. After washing, the slides were left to air dry in a dark environment until microscopic analysis.

### Fluorescence microscopic analysis

**Figure 1:**
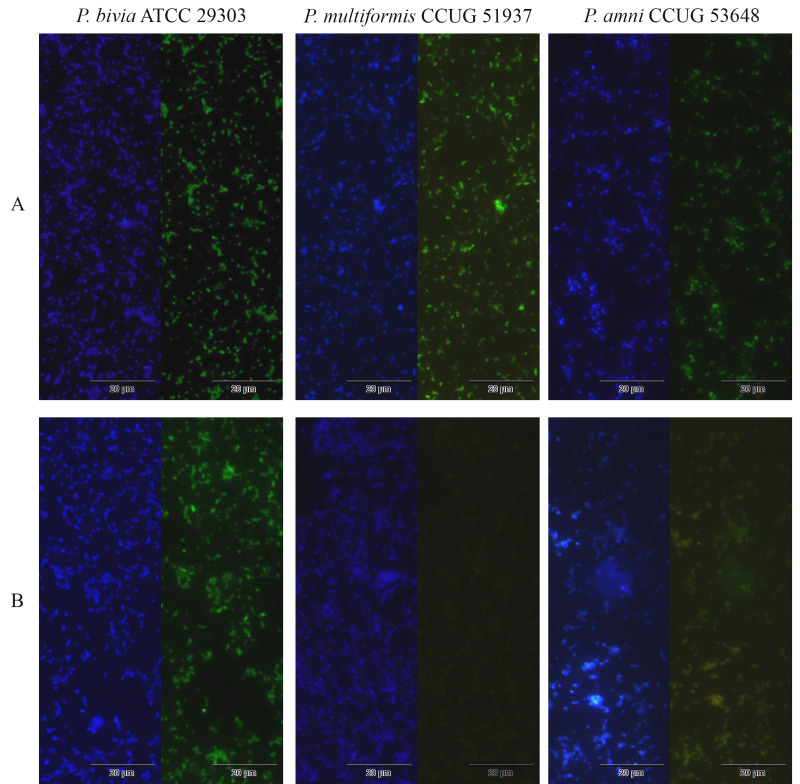
Fluorescence microscopy results of *Prevotella* spp. (A) and *P. bivia* (B) probe hybridizations. The images were obtained by DAPI staining (DAPI filter) and *Prevotella* spp. or *P. bivia* probe hybridizations (FITC filter) with the strains *P. bivia* ATCC 29303, *P. multiformis* CCUG51937, and *P. amnii* CCUG 53648. Images with a magnification of 400x; scale bars represent 20 µm.

Microscopic analysis was performed using an Olympus BX51 epifluorescence microscope (Olympus, Lisbon, Portugal) with the FITC filter (BP 470-490, FT500, LP 516 sensitive to the Alexa Fluor 488 molecule). The fluorescence signal of the probe was observed using the filter FITC and the other filters were used to discriminate any autofluorescence from the cells. In each experiment, a negative control was included with hybridization solution without a probe. The experiments were performed with at least two independent assays. Images were acquired at 40 × magnification, with the same exposure time used for both target and non-target species. Hybridizations results were evaluated qualitatively according to the following classification: (−) Absence of hybridization; (+) Poor hybridization; (+ +) Moderate hybridization; (+ +  +) Good hybridization. Figure 1 shows examples of microscopic images obtained from the hybridization of *Prevotella* spp. (Fig. 1A) and *P. bivia* (Fig. 1B) probes tested on *P. bivia* ATCC 29303, *P. multiformis* CCUG 51937 and *P. amnii* CCUG 53648. For the determination of the experimental sensitivity and specificity of the *Prevotella* spp. probe, 28 different strains of *Prevotella* representing 24 different species and 38 other BVAB were used, respectively. The sensitivity and specificity of the novel *Prevotella* spp. probe were calculated, as described previously (Almeida et al., 2013). Briefly, specificity was calculated as (nPs/TnP) × 100, where nPs was the number of non-*Prevotella* strains that did not react with the probe, and TnP was the total number of non-*Prevotella* strains examined. Sensitivity was calculated as (Ps/TPs) × 100, where Ps was the number of *Prevotella* strains detected by the probe and TPs was the total number of *Prevotella* strains tested (Almeida et al., 2013).

### Recruitment of participants

Potential participants were recruited in the Birmingham, Alabama Metropolitan Area for participation in a BV pathogenesis study. Women presented to the University of Alabama at Birmingham (UAB) Sexual Health Research Clinic for screening. Eligible women signed a screening written informed consent form prior to engaging in any study-related procedures. They provided urine for a pregnancy test and were then tested for BV by the Amsel criteria and Nugent score (Amsel et al., 1983; Nugent, Krohn & Hillier, 1991). Participants were excluded from the study if they were found to be pregnant, had self-reported HIV infection, were currently on their menses, or took any oral or intravaginal antibiotics within the past 14 days, as previously described (Muzny et al., 2024). Non-pregnant women with no Amsel criteria and a normal Nugent score of 0–3 were subsequently enrolled into the study after enrollment written informed consent was obtained. Enrolled women completed an enrollment questionnaire on their sociodemographic characteristics, sexual history (sexual partners, STI history), hygienic practices, contraception methods, and substance use. Next, a vaginal swab was obtained from women to test for *Chlamydia trachomatis*, *Trichomonas vaginalis*, *Neisseria gonorrhoeae*, and *Mycoplasma genitalium* by nucleic acid amplification testing (NAAT). Enrolled participants without current STIs were taught how to self-collect vaginal specimens on a twice daily basis. They were also asked to complete a daily diary to document their sexual practices, vaginal symptoms, medication use, and menstrual cycle days. The study duration for each participant was 60 days or until iBV was diagnosed (defined as a Nugent score 7–10 on at least four consecutive vaginal specimens). Throughout the study, participants self-collected three vaginal swab specimens twice daily for 60 days that were dropped off at the research clinic on a weekly basis. Participants used one of these swab specimens to smear a slide for Nugent score determination at each time point. After drop-off, the smeared slides were Gram stained in the research laboratory and examined per the Nugent scoring protocol to determine if participants developed iBV over the course of the study. All vaginal specimens collected were stored at −80 °C; one of the three specimens collected at each time point was used for PNA-FISH methods. This study was approved by the University of Alabama at Birmingham Institutional Review Board (Protocol #IRB-300004547).

### Vaginal specimen selection

Women who developed iBV over the course of the study (iBV cases) were matched by age, race, and contraceptive method on a 1:1 basis to women maintaining an optimal vaginal microbiota (Nugent score 0–3) for the majority (≥85% of days) of the study (non-cases). Non-case specimens were matched to case specimens by day of menses. In order to test the efficacy of the newly developed *Prevotella* spp. probe, iBV case specimens with a higher abundance of *Prevotella* spp. present determined by 16S rRNA sequencing were chosen to compare to their respective matched non-case specimens (unpublished data). A total of 18 vaginal specimens from nine iBV cases and nine non-cases were selected to compare the *Prevotella* spp. probe and the *P. bivia* probe.

### Fluorescent imaging and quantification

Fixed and hybridized vaginal specimens from iBV cases and non-cases were imaged using the FITC filter on the NanoZoomer S60 Slide Scanner (Hamamatsu Corporation). Six fluorescent images were captured per specimen, in line with the Nugent scoring protocol used to diagnose participants with iBV. Following image capture, Fiji ImageJ 1.8.0 was used to quantify the bacteria present in the images. The signal-to-background contrast threshold was optimized by the user to detect the bacterial cells without background interference. Cells that were separate, but touching, were separated by the software using the watershed tool, in which the software predicts the separation between different cells. Bacterial cells were detected by their pixel intensity compared to the background and automatically quantified by the software.

### Statistical analysis

GraphPad Prism 10.0.2 was used for all statistical analyses. Statistical differences between the probes in iBV cases and non-cases were determined by the Mann–Whitney U test, since the bacterial counts were non-normally distributed. A *p*-value of ≤ 0.05 was considered statistically significant.

**Table 1:** Theoretical specificities and sensitivities of PNA probes for detecting *Prevotella* spp. based on 23S rRNA. Technical parameters of all probes designed *in silico*.

Sequence (5′→ 3′)	Length (bp)	%GC	ΔG	Tm (°C)	No. of *Prevotella* strains detected	No. of non- *Prevotella* strains detected	Total strains detected	No. of *Prevotella* strains RefNR database	No. of non- *Prevotella* strains RefNR database	Specificity (%)^a^	Sensitivity (%)^b^
AGGCTCGCTTTCACT	15	53.3	−18.03	72.99	165	324	489	183	95,027	99.7%	90.2%
TCTTGACCTATCGGT		46.7	−16.12	69.50	132	1	133			100.0%	72.1%
GGAGTTCGTCAGGTC		60	−18.66	81.50	153	1	154			100.0%	83.6%
CGCCCCCTTACTGCA		66.7	−20.04	79.42	146	1	147			100.0%	79.8%
TAACTTAGACAGAAT		26.7	−12.83	66.62	96	0	96			100.0%	52.5%
TCGAGTTAGCACAGC		53.3	−17.49	76.12	72	0	72			100.0%	39.3%
GGATGACCAAGGTCA		53.3	−17.78	84.14	135	2	137			100.0%	73.8%
AGACCTCAGACAGCC		60	−17.66	79.31	142	2	144			100.0%	77.6%
CGGCATGTCTGCCTC		66.7	−19.91	79.18	147	12	159			100.0%	80.3%

**Notes.**

a Specificity was calculated as [1-(nP/TnP)] ×100, where nP stands for the number of non-*Prevotella* strains that did not react with the probe and TnP for the total of non-*Prevotella* strains examined.

b Sensitivity was calculated as P/(TP)×100, where P stands for the number of *Prevotella* strains detected by the probe and TP for the total number of *Prevotella* strains existent in the database.

## Results

### Design and *in silico* analysis of the *Prevotella* spp. PNA probe

Alignment of the 16S and 23S rRNA sequences from the four vaginal *Prevotella* spp. selected (*P. bivia*, *P. amnii*, *P. disiens*, and *P. timonensis*) and closely related non-*Prevotella* spp. revealed several conserved regions unique to the target group. Candidate regions for the probe design were chosen based on the number of *Prevotella* strains detected, the position of mismatches in closely related sequences, GC content, melting temperature (T_m_), and Gibbs free energy (ΔG). A small number of 16S rRNA candidate probes were also identified but were not prioritized due to lower specificity and higher cross-reactivity with non-*Prevotella* sequences. For the 23S rRNA dataset, nine candidate probes were identified (Table 1). *In silico* evaluation was performed using the ARB-SILVA TestProbe tool (no mismatches allowed) against the large subunit (23S) rRNA database, which contained 183 *Prevotella* entries and 95,027 non-target sequences. Most probes showed 100% predicted specificity, with sensitivities ranging from 39.3% to 90.2%. GC content ranged from 26.7% to 66.7%, T_m_ from 66.62 °C to 84.14 °C, and ΔG from −12.83 to −20.04 kcal/mol. The probe AGGCTCGCTTTCACT displayed the highest sensitivity (90.2%) and near-perfect specificity (99.7%). Based on its high sensitivity, and favorable thermodynamic parameters, this 23S rRNA probe was selected for synthesis. The sequence exhibited a T_m_ of 72.99 °C and ΔG of −18.03 kcal/mol and was synthesized with an N-terminal conjugation to Alexa Fluor 488 *via* a double 8-amino-3,6-dioxaoctanoic acid linker for downstream application.

### Optimization of experimental conditions of FISH procedure

Several factors, such as pH and probe concentration, can affect the PNA-FISH procedure and influence the fluorescence signal of the probe (Rui et al., 2016). Fixation and permeabilization steps can also affect the results (Rocha, Almeida & Azevedo, 2018). Therefore, preliminary experiments were conducted to evaluate different hybridization times and temperatures to maximize probe signal. Optimization assays (Table 2) identified 56 °C and 60 min as the optimal conditions, which were subsequently applied to determine the analytical sensitivity and specificity of the probe.

**Table 2:** Optimization results of the hybridization of PNA *Prevotella* spp. probe with the strain *P. bivia* ATCC 29303 for the different temperatures and times tested.

Temperature (°C)	Time (min)	Hybridization results
52	60	+
	90	++
56	60	+++
	90	++
58	60	++
	90	++
60	60	+
	90	+
65	60	+
	90	+

**Notes.**

Hybridization results were evaluated qualitatively according to the classification: (-) Absence of hybridization; (+) Poor hybridization; (++) Moderate hybridization; (+++) Good hybridization.

### Determination of *Prevotella* spp. probe analytical sensitivity and specificity

After optimizing the hybridization conditions for the new probe, we evaluated its analytical performance. *In vitro* validation included assessment of probe sensitivity and specificity using a diverse panel of *Prevotella* strains and non-target BVAB. These assays aimed to confirm the ability of the probe to reliably detect different *Prevotella* spp. while minimizing cross-reactivity. For comparative purposes, and to establish a reference for subsequent analysis of vaginal samples, hybridizations was also performed using the previously developed *P. bivia*-specific probe. The new *Prevotella* spp. probe successfully hybridized with 27 out of the 28 strains, yielding an analytical sensitivity of 96.4%, 95% confidence interval (CI) of [81.7%–99.9%], while the *P. bivia* probe only had a 44% success binding to the *Prevotella* strains (Table 3).

**Table 3:** Optimization results of the hybridization of PNA *Prevotella* spp. probe with the strain *P. bivia* ATCC 29303 for the different temperatures and times tested. Results of hybridization of *Prevotella* spp. PNA probe and *P. bivia* probe with different strains of *Prevotella* species.

Strain	Reference	Hybridization results for *Prevotella* spp. probe	Hybridization results for *P. bivia* probe
*Prevotella amnii*	CCUG 53648	++	+
*Prevotella bivia*	ATCC 29303	+++	+++
*Prevotella bivia*	CCUG 33360	+++	+++
*Prevotella bivia*	CCUG 34046	++	++
*Prevotella bivia*	CCUG 44195	+++	+++
*Prevotella bivia*	CCUG 59496	++	+++
*Prevotella brunnea*	CCUG 72809	–	–
*Prevotella buccalis*	CCUG 44127	++	-*
*Segatella copri (Prevotella)*	CCUG 58058T	+++	–
*Prevotella corporis*	CCUG15404	+++	-*
*Prevotella dentalis*	CCUG48288	+++	+
*Prevotella denticola*	CCUG 29542T	+++	–
*Prevotella disiens*	CCUG 59491	+++	–
*Prevotella fusca*	CCUG 57946	+	+
*Prevotella histicola*	CCUG 55407	++	+
*Prevotella illustrans*	CCUG 72806	+++	–
*Prevotella imum*	CCUG 65911	+++	+
*Prevotella intermedia*	CCUG 31410	+++	–
*Prevotella jejuni*	CCUG 60371	+++	+
*Prevotella melaninogenica*	CCUG 65141	+++	-*
*Prevotella micans*	CCUG 56105	+++	+
*Prevotella multiformis*	CCUG 51937	+++	–
*Prevotella nigrescens*	CCUG 25289	++	–
*Prevotella pallens*	CCUG 39484	+	-*
*Prevotella scopos*	CCUG 57945	+++	-*
*Hoylesella timonensis (Prevotella)*	CCUG 59487	+++	–
*Prevotella veroralis*	CCUG 15422	+++	-*
*Prevotella vespertine*	CCUG 72808	+++	-*

**Notes.**

Hybridization results were evaluated qualitatively according to the classification: (-) Absence of hybridization; (+) Poor hybridization; (++) Moderate hybridization; (+++) Good hybridization.

Specificity was then evaluated using 38 non-*Prevotella* BVAB that are commonly associated with the vaginal microenvironment. No cross-hybridization was observed, resulting in a specificity of 100%, 95% CI of [90.8%–100.0%]. (Table 4). Figure 1 depicts an example of the hybridization comparisons with both probes, while Figs. S1 and S2 presents all bacteria tested.

**Table 4:** Determination of analytical specificity of *Prevotella* species probe. Results of hybridization of *Prevotella* spp. PNA probe with different species.

Species	Reference	Hybridization results
*Acinetobacter baumannii*	CCUG 59798	–
*Actinomyces neuii*	UM067	–
*Actinomyces urogenitalis*	CCUG 44038	-*
*Aerococcus christensenii*	CCUG 28826	–
*Bacillus firmus*	UM034	-^*^
*Bifidobacterium bifidum*	CCUG 59492	-^*^
*Brevibacterium ravenspurgense*	CCUG 42923	-^*^
*Corynebacterium tuscaniense*	UM137	-^*^
*Enterococcus faecalis*	UM035	-^*^
*Escherichia coli*	UM056	–
*Fannyhessea vaginae*	ATCC BAA-55	-^*^
*Gardnerella leopoldii*	UM034	-^*^
*Gardnerella piotii*	UM035	–
*Gardnerella swidsinskii*	UM094	–
*Gardnerella vaginalis*	ATCC 14018	-^*^
*Gemella haemolysans*	UM034	–
*Lactobacillus crispatus*	EX533959VCO6	–
*Lactobacillus gasseri*	ATCC 9857	-^*^
*Lactobacillus iners*	ATCC 55195	–
*Lactobacillus rhamnosus*	CECT 288	-^*^
*Lactobacillus vaginalis*	UM062	-^*^
*Megasphaera micronuciformis*	CCUG 45952T	–
*Mobiluncus curtisii*	ATCC 35241	–
*Mobiluncus mulieris*	ATCC 35239	–
*Mycoplasma hominis*	UM054	–
*Neisseria gonorrhoeae*	CCUG 13281	–
*Nosocomiicoccus ampullae*	UM121	-^*^
*Peptostreptococcus anaerobius*	ATCC 27337	-^*^
*Porphyromonas asaccharolytica*	CCUG 7834T	–
*Cutibacterium acnes*	UM034	-^*^
*Shigella* spp.	UM137	–
*Sneathia sanguinegens*	CCUG 66076	–
*Staphylococcus epidermidis*	UM066	–
*Staphylococcus haemolyticus*	UM066	–
*Staphylococcus hominis*	UM224	–
*Staphylococcus saprophyticus*	UM121	–
*Staphylococcus simulans*	UM059	-^*^
*Veillonella parvula*	CCUG 59474	–

**Notes.**

Hybridization results were evaluated qualitatively according to the classification: (-) Absence of hybridization; (+) Poor hybridization; (++) Moderate hybridization; (+++) Good hybridization

* These species showed some autofluorescence signal detected in the FITC filter.

### Detection of *Prevotella* species in vaginal swab specimens using the *Prevotella* spp. and *P. bivia* probes

**Figure 2:**
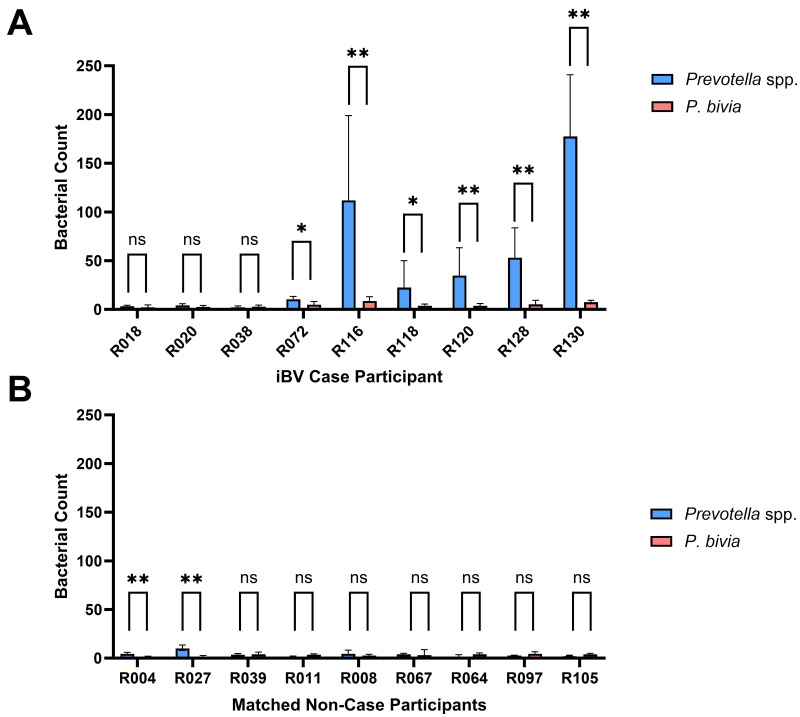
Quantification of *Prevotella.* species in vaginal specimens from iBV cases (A) and non-cases (B), with *Prevotella* spp. and *P. bivia* probes. PNA-FISH quantification was performed using a Slide Scanner. For each vaginal specimen, five representative fields were imaged, and the average bacterial counts were determined following the Nugent scoring method. Results are expressed as mean values with error bars representing standard deviation.

**Figure 3:**
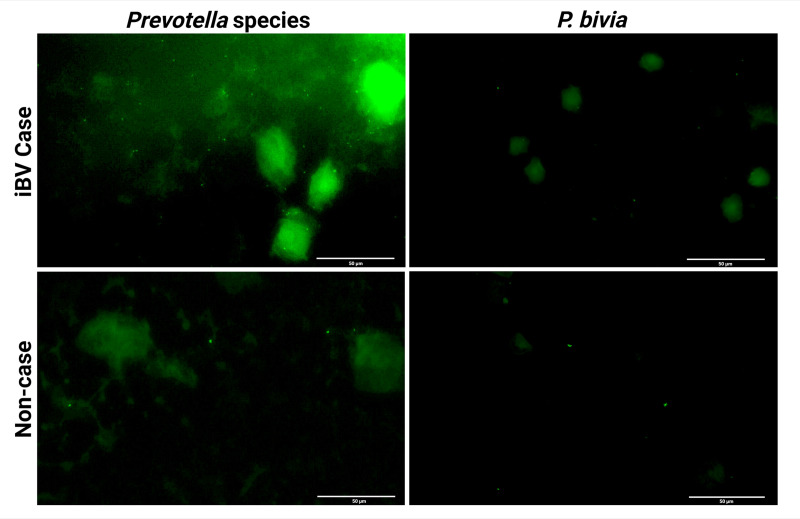
Example of a fluorescence microscopy images obtained from vaginal swab specimens of iBV cases and non-cases with *Prevotella* spp. or *P. bivia* probes. The scale bars represent 50 µm. Other examples are presented in Fig. S3.

Following *in vitro* validation, we applied both the *Prevotella* spp. probe and the *P. bivia*-specific probe to vaginal swab specimens in order to compare their performance in a clinical context. This analysis was designed to determine whether the newly developed probe could improve the detection of *Prevotella* spp. *ex vivo*, particularly in cases where 16S sequencing suggested high *Prevotella* abundance (data unpublished), but low *P. bivia* probe signal was observed (George et al., 2025). The results shown in Fig. 2 indicate that the *Prevotella* spp. probe consistently detected a higher bacterial count in vaginal swab specimens when compared to the *P. bivia*-specific probe. Curiously, in 3/9 (33.3%) iBV cases where *Prevotella* spp. were expected, only minimal signal was detected with either probe, indicating a very low abundance of these species. Interestingly, within non-case samples, *Prevotella* spp. were significantly lower than within iBV cases and, in 2/9 (22.2%) of vaginal specimens, we detected significantly higher *Prevotella* spp. counts than *P. bivia* counts. The vaginal swab specimens PNA analysis is demonstrated in Fig. 3.

## Discussion

The diagnosis of BV relies on classical methods, such as the Amsel criteria and the Nugent score (Nugent, Krohn & Hillier, 1991; Amsel et al., 1983) that evaluate the vaginal pH, whiff test, presence of clue cells, and presence of a homogeneous grey-white vaginal discharge (Amself criteria) and the bacterial morphologies present on a vaginal Gram stain (Nugent score), respectively. While the evaluation of the Amsel criteria does not provide any information regarding the pathogenic agents of the infection, the Nugent score only evaluates for the morphologies of some BVAV present in the vaginal smear (*Lactobacillus* spp., *Gardnerella* spp., and *Mobiluncus* spp.), missing the detection/identification of other important species present in BV cases. Our findings advance BV diagnostics by introducing a genus-level *Prevotella* PNA-FISH probe that improves detection where species-specific tools underperform. Compared with the *P. bivia* probe, the *Prevotella* spp. probe identified greater bacterial counts in most BV-positive specimens (6/9), indicating that multiple *Prevotella* spp., not only *P. bivia*, are relevant *in vivo*. This complements other diagnostic tests that lack the detection of other key BVAB, such as *Prevotella*. Furthermore, by retaining spatial context, PNA-FISH can visualize genus-level *Prevotella* spp. within biofilm architecture along with other key BVAB (*Gardnerella* and *Fannyhessea*).

PNA probes have emerged as a promising alternative, offering high specificity and stability due to their synthetic nature (Prudent & Raoult, 2019). PNA probes can bind to specific DNA or RNA sequences, enabling their use in FISH assays. FISH allows for the direct visualization of target bacteria in clinical samples, such as in vaginal swab samples, without the need for cultivation, providing both qualitative and spatial data regarding microbial communities (Frickmann et al., 2017). The application of PNA probes, particularly in BV pathogenesis research, has been demonstrated in previous studies, particularly with regards to detecting vaginal bacteria such as *Lactobacillus* spp. (Machado et al., 2013), *Gardnerella* spp. (Machado et al., 2016)*, F. vaginae* (Hardy et al., 2015) and *P. bivia* (Sousa et al., 2023). These studies underscore the utility of PNA probes in enhancing diagnostic accuracy and understanding microbial interactions in BV.

Herein, we designed and validated a novel PNA probe targeting vaginal *Prevotella* spp. to test our evolving hypothesis that other *Prevotella* spp. beyond *P. bivia* might contribute to the pathogenesis of iBV. This hypothesis emerged from unexpected observations in our recently published work (George et al., 2025), where *P. bivia* detection remained low and comparable between iBV cases and non-cases, despite prior evidence implicating this species in iBV (Muzny et al., 2018; Gilbert et al., 2019) Given the well-documented limitations of 16S rRNA gene sequencing in resolving members of the *Prevotella* genus (Chen et al., 2015), we proposed that a broader detection approach could reveal the presence of other clinically relevant *Prevotella* spp. that might be undetected using species-specific tools. The development of a wider-targeted probe, followed by its analytical validation and application to clinical vaginal specimens, allowed us to explore this possibility.

As expected, our *in vitro* data showed that the *Prevotella* spp. probe displays a markedly higher sensitivity (96%) for detecting diverse *Prevotella* spp. compared to the *P. bivia*-specific probe, which only detected 44% of the tested strains. Of note, these values should be interpreted with caution, as sensitivity and specificity assessments are influenced by the number and phylogenetic diversity of strains included in the analysis. Nevertheless, this result is consistent with the broader taxonomic range of the *Prevotella* spp. probe. Despite its broader detection range, the *Prevotella* spp. probe did not hybridize with the *P. brunnea* strain used in this study. This does not represent a significant limitation in the context of vaginal microbiota research, as this species is rarely, if ever, reported in vaginal samples or associated with BV in the literature (Tett et al., 2021).

It is also important to acknowledge that *P. copri* (Blanco-Míguez et al., 2023) and *P. timonensis* (Oren & Göker, 2023) have recently been proposed as new species, following reclassification efforts based on whole-genome comparisons and average nucleotide identity (ANI) thresholds (Hitch et al., 2022). These changes reflect the growing refinement of bacterial taxonomy and highlight the limitations of 16S rRNA-based classification in resolving closely related species (Vale, Tanoeiro & Marques, 2022). Despite their reclassification, both species remain phylogenetically close to the *Prevotella* genus and belong to the same family (*Prevotellaceae*), sharing highly similar rRNA gene regions. Given that our *Prevotella* spp. probe was designed to detect the genus based on 23S rRNA sequences currently available, the detection of *P. copri* and *P. timonensis* is expected, and consistent with the probe’s intended target. Nonetheless, if a more conservative approach is taken, and we exclude these two species from the genus, the calculated sensitivity and specificity would be slightly adjusted to 96% and 95%, respectively. This emphasizes the importance of accounting for ongoing taxonomic revisions when interpreting molecular probe-based detection data.

When testing both probes in clinical vaginal samples, the *Prevotella* spp. probe revealed significantly higher detection (*p* < 0.05) levels in the majority of BV positive specimens compared to the *P. bivia* probe. However, in 3 cases, a low signal was detected, despite the detection of *Prevotella* spp. by 16S sequencing (unpublished data). This discrepancy could be a result of technical limitations. On one hand, the taxonomic resolution of 16S rRNA sequencing is often insufficient to differentiate among closely related species and can sometimes result in misclassification at the genus level due to shared sequence similarity (Janda & Abbott, 2007). On the other hand, the very low FISH signal could be due to a low bacterial load (since FISH does not rely in amplification strategies), RNA degradation, or fixation-related artifacts that affect probe hybridization efficiency (Almeida & Azevedo, 2021). Interestingly, in 2/9 (22.2%) non-BV case specimens, the *Prevotella* spp. probe also detected significantly higher cell counts (*p* < 0.05) than the *P. bivia* probe, albeit at lower levels than those found in BV-positive specimens.

FISH remains an attractive technique for microbiological diagnostics due to its rapid turnaround time, relatively low operational cost, and the ability to directly visualize target microorganisms *in situ*, which facilitates bacterial identification in clinical samples. However, several disadvantages limit its routine use. The main challenge lies in image analysis, which requires trained personnel and critical interpretation, since no standardized protocols exist for signal quantification or fluorescence evaluation, leading to variability between operators and sample types. Additionally, while the *Prevotella* spp. probe enhances visualization of genus-level diversity, FISH inherently has lower sensitivity than sequencing-based approaches for detecting low-abundance species (Frickmann et al., 2017). Because FISH depends on rRNA copy number and cell integrity, bacteria present in very low abundance or in a metabolically inactive state may fall below the microscopic threshold of detection, leading to underestimation of community diversity (Prudent & Raoult, 2019). In our *in vitro* assays, the use of freshly cultured bacteria grown for 48 h ensured high viability and consequently abundant ribosomes, supporting strong and reliable probe binding. For clinical vaginal specimens, ribosomal content could not be standardized due to physiological variation in vivo; however, validated collection and handling procedures were implemented to preserve cellular and RNA integrity, including storage at 4 °C immediately after sampling and subsequent freezing at −80 °C. Although ribosomal concentration was not quantitatively measured, the clear, specific, and reproducible hybridization patterns observed across both *in vitro* and clinical samples indicate ribosomal stability was sufficient to enable robust detection under the conditions of this study. Still, while FISH provides high specificity and structural insight into microbial localization within biofilms, it should be interpreted as complementary rather than substitutive to sequencing approaches.

## Conclusions

Although the number of direct vaginal specimens analysed in this study was limited, it is important to emphasize that the objective of this study was not to estimate the prevalence of *P. bivia* or other *Prevotella* spp. in the vaginal microbiota, but rather to validate the specificity of the newly designed PNA-FISH probe. For this methodological purpose, the selected sample set was sufficient to consistently demonstrate that samples previously classified as containing only *P. bivia* by 16S rRNA sequencing actually contained a mixed *Prevotella* population, highlighting the discriminatory capability of the probe. Building on this, while previous research has highlighted *P. bivia* as a possible early bacterial colonizer during iBV (Muzny et al., 2018; Gilbert et al., 2019), our findings suggest a broader contribution of other *Prevotella* species to BV-associated microbial communities, which has also recently been demonstrated elsewhere (Dillard et al., 2025). These results support the hypothesis that multiple *Prevotella* spp., not only *P. bivia*, may contribute to the pathogenesis of iBV. The broader detection achieved with our *Prevotella* spp. probe highlights its potential as an informative tool for studying the vaginal microbiota.

## Supplemental Information

10.7717/peerj.20902/supp-1 Supplemental Information 1 Raw data from the experiments Reporting each group of experiments: Probe optimization; In vitro sensititvy, In vitro specificity, In vivo.

10.7717/peerj.20902/supp-2 Supplemental Information 2 Isolation source of the *Prevotella spp.* used in this study, as obtained in the CCUG collection website

10.7717/peerj.20902/supp-3 Supplemental Information 3 Supplementary Figures

## Additional Information and Declarations

### Data Availability

The following information was supplied regarding data availability: The raw data is available in the Supplemental File.
