# Supplementary material for: Evidence from a broad-range PNA probe links several Prevotella species to bacterial vaginosis
Source: PeerJ. 2026 Mar 26;14:e20902. doi: 10.7717/peerj.20902 (PMC13033284; doi:10.7717/peerj.20902)
Supplement: Supplemental Information 2 [file peerj-14-20902-s002.docx]

**Supplementary Table 1:** Isolation source of the *Prevotella spp.* used in this study, as obtained in the CCUG collection website.

| Strain | Reference | Isolation Source |
| --- | --- | --- |
| *Prevotella amnii* | CCUG 53648 | Human amniotic fluid, turbid and ill smelling |
| *Prevotella bivia* | ATCC 29303 | Endometrium |
| *Prevotella bivia* | CCUG 33360 | Human |
| *Prevotella bivia* | CCUG 34046 | Human vagina |
| *Prevotella bivia* | CCUG 44195 | Human amniotic membranes |
| *Prevotella bivia* | CCUG 59496 | Human vagina |
| *Prevotella brunnea* | CCUG 72809 | Human clinical sample |
| *Prevotella buccalis* | CCUG 44127 | Human vagina, fornix, vaginosis |
| *Prevotella copri#* | CCUG 58058T | Human feces, healthy person |
| *Prevotella corporis* | CCUG15404 | Cervical swab |
| *Prevotella dentalis* | CCUG48288 | Human dental root canal |
| *Prevotella denticola* | CCUG 29542T | Dental plaque |
| *Prevotella disiens* | CCUG 59491 | Human vagina |
| *Prevotella fusca* | CCUG 57946 | Human oral cavity, periodontic subgingival plaque |
| *Prevotella histicola* | CCUG 55407 | Human oral tissue, squamous cell carcinoma |
| *Prevotella illustrans* | CCUG 72806 | Human clinical sample |
| *Prevotella imum* | CCUG 65911 | Human periodontal abscess |
| *Prevotella intermedia* | CCUG 31410 | Human blood |
| *Prevotella jejuni* | CCUG 60371 | Jejunal biopsy of celiac disease patient |
| *Prevotella melaninogenica* | CCUG 65141 | Human wound |
| *Prevotella micans* | CCUG 56105 | Human oral necrotic pulp |
| *Prevotella multiformis* | CCUG 51937 | Human subgingival plaque |
| *Prevotella nigrescens* | CCUG 25289 | Human periodontal abscess |
| *Prevotella pallens* | CCUG 39484 | Human saliva |
| *Prevotella scopos* | CCUG 57945 | Human oral cavity, dental implant |
| *Prevotella timonensis##* | CCUG 59487 | Human vagina |
| *Prevotella veroralis* | CCUG 15422 | Human oral cavity |
| *Prevotella vespertine* | CCUG 72808 | Human clinical sample |

# recently reclassified as *Segatella copri* (doi:10.1016/j.chom.2023.09.013)

## recently reclassified *as Hoylesella timonensis* (doi: 10.1099/ijsem.0.005709)
