# Supplementary material for: Evidence from a broad-range PNA probe links several Prevotella species to bacterial vaginosis
Source: PeerJ. 2026 Mar 26;14:e20902. doi: 10.7717/peerj.20902 (PMC13033284; doi:10.7717/peerj.20902)

Supplementary Figure 1: The following images present the fluorescence microscopy results of *Prevotella* spp. probe hybridization with all the tested species for sensitivity and specificity. The images were obtained using DAPI filter and FITC filter. Magnification of 400x and scale bars represent 20 µm.

Acinetobacter baumannii CCUG 59798

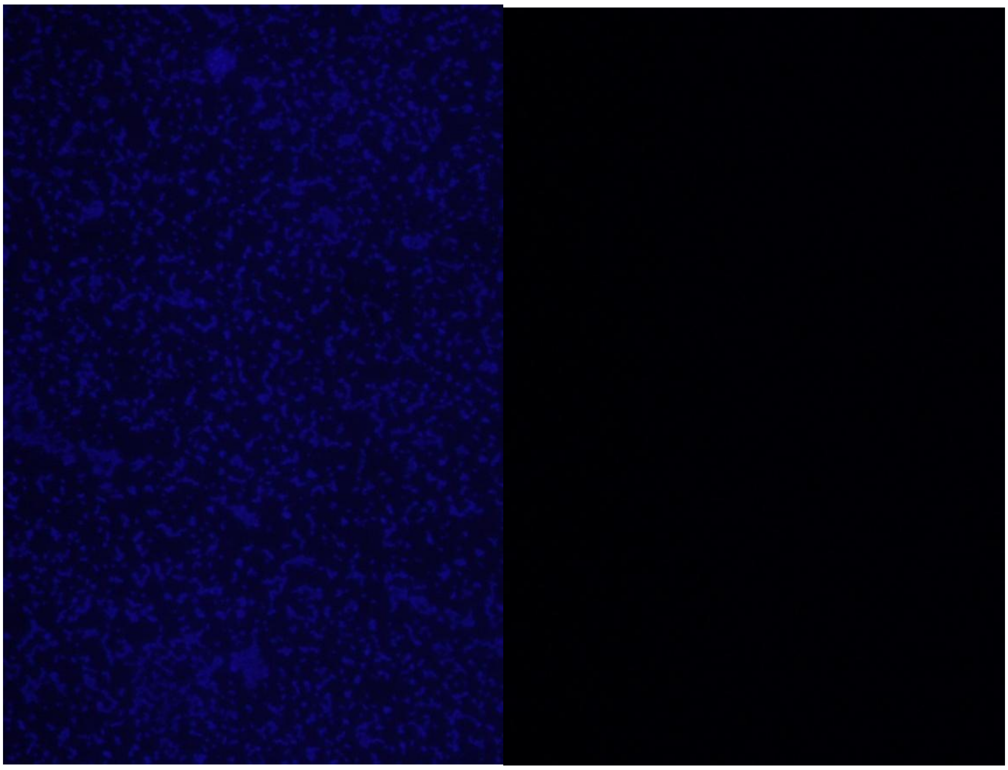

Actinomyces neuii UM067

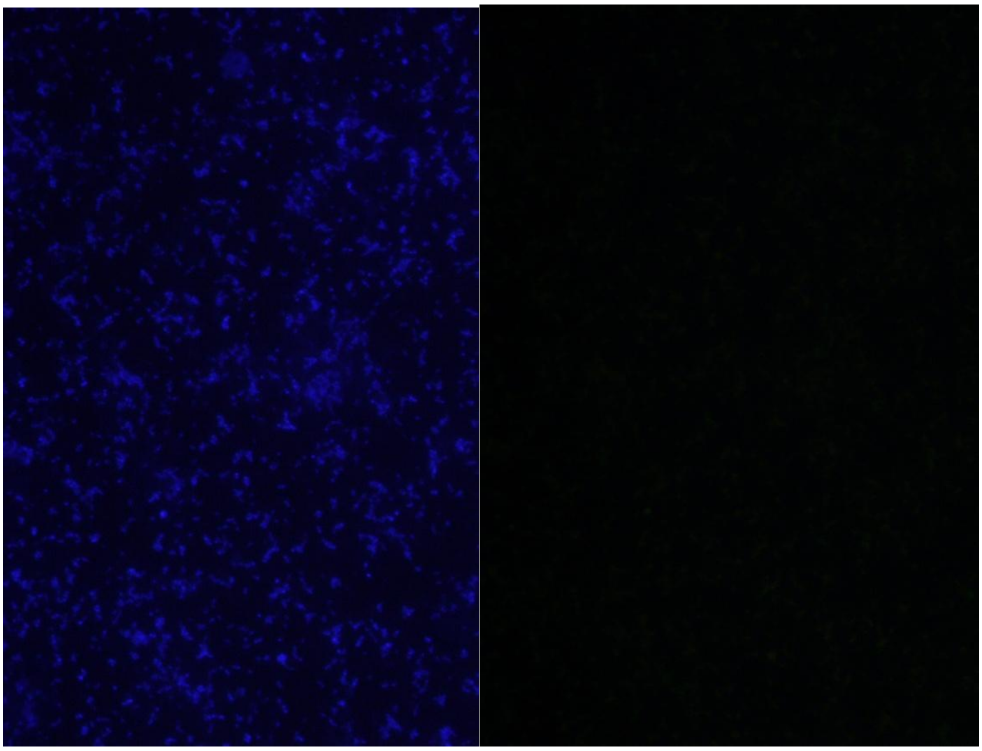

Actinomyces urogenitalis   CCUG 44038

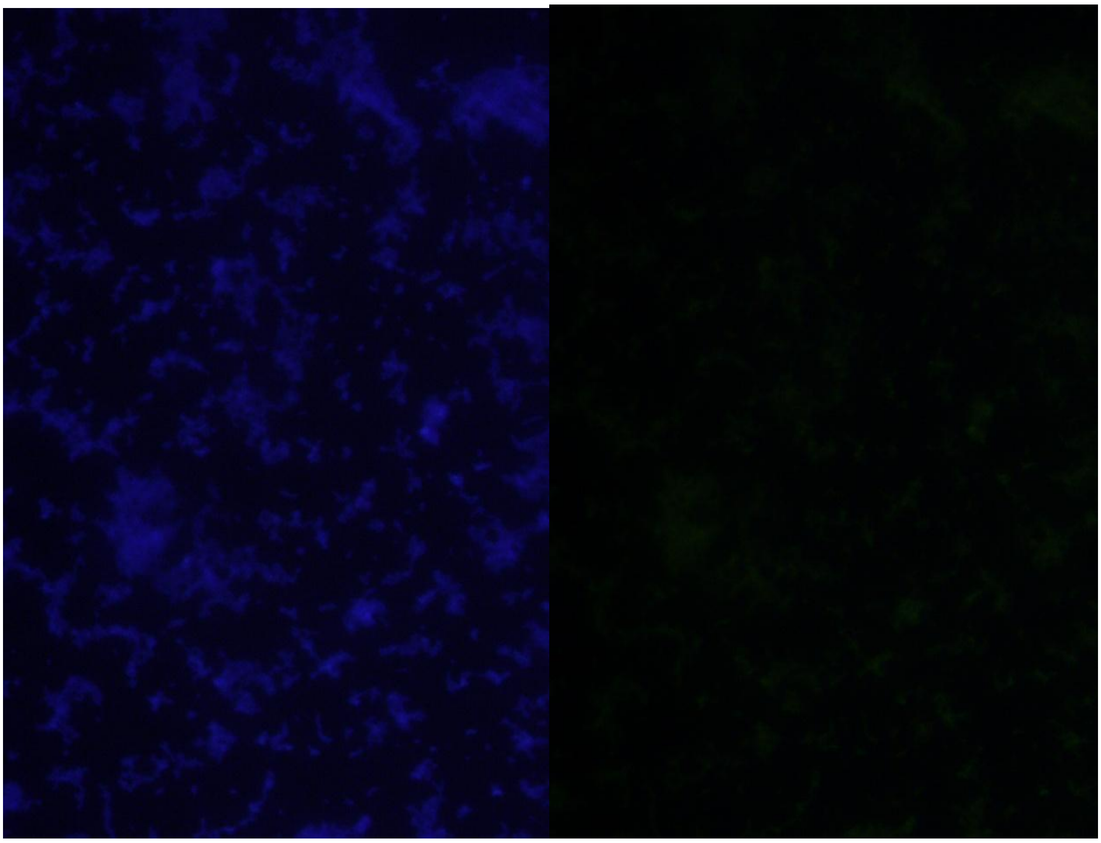

Aerococcus christensenii   CCUG 28826

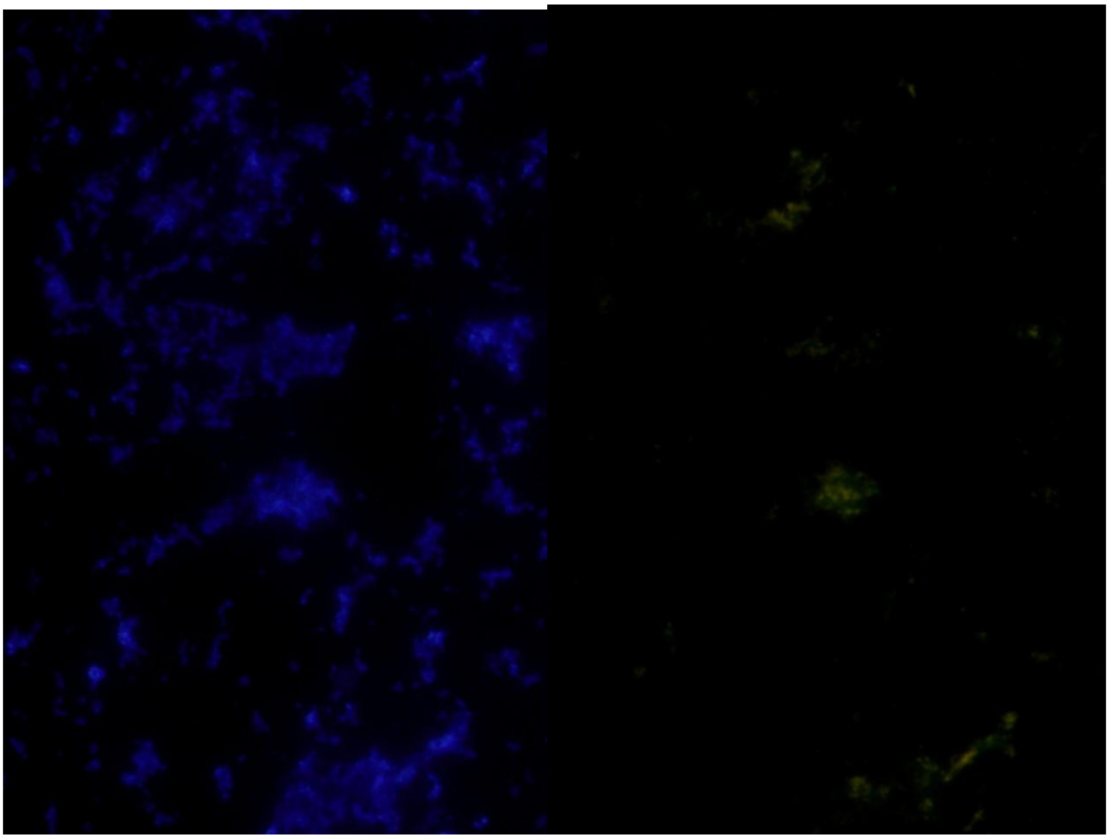

Bacillus firmus UM034

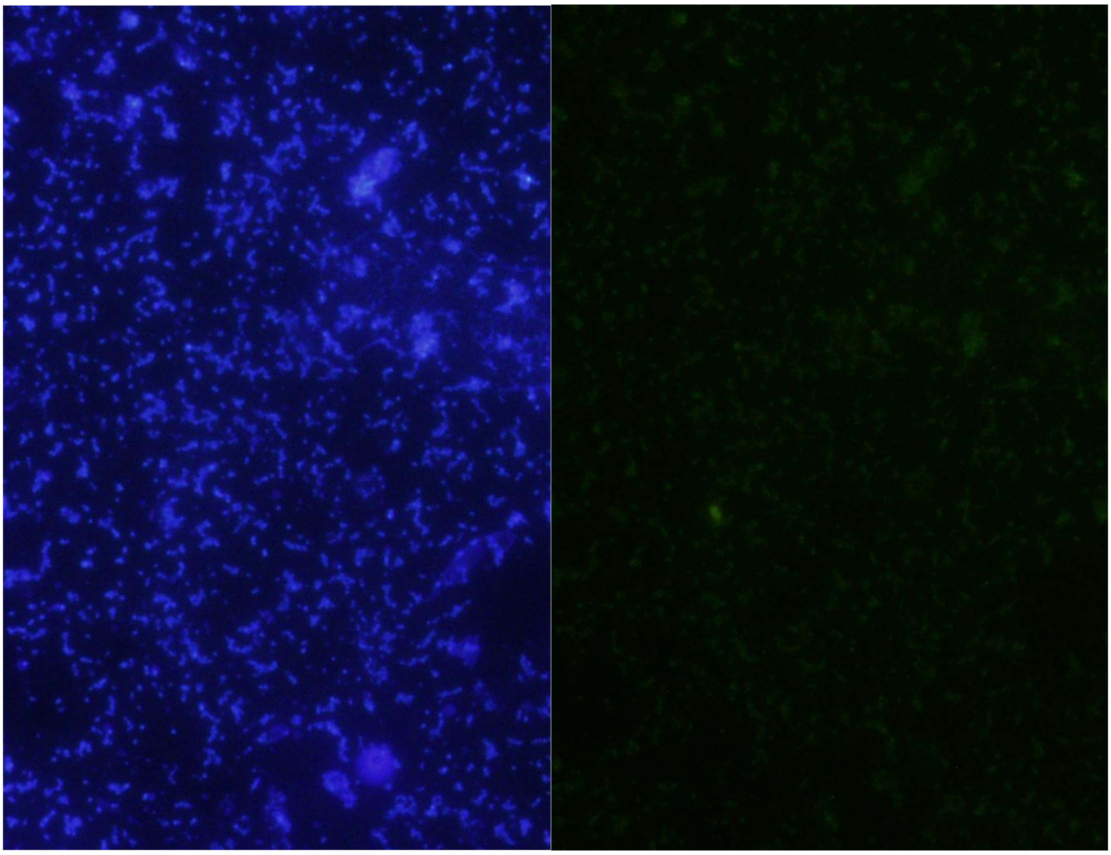

Bifidobacterium bifidum CCUG 59492

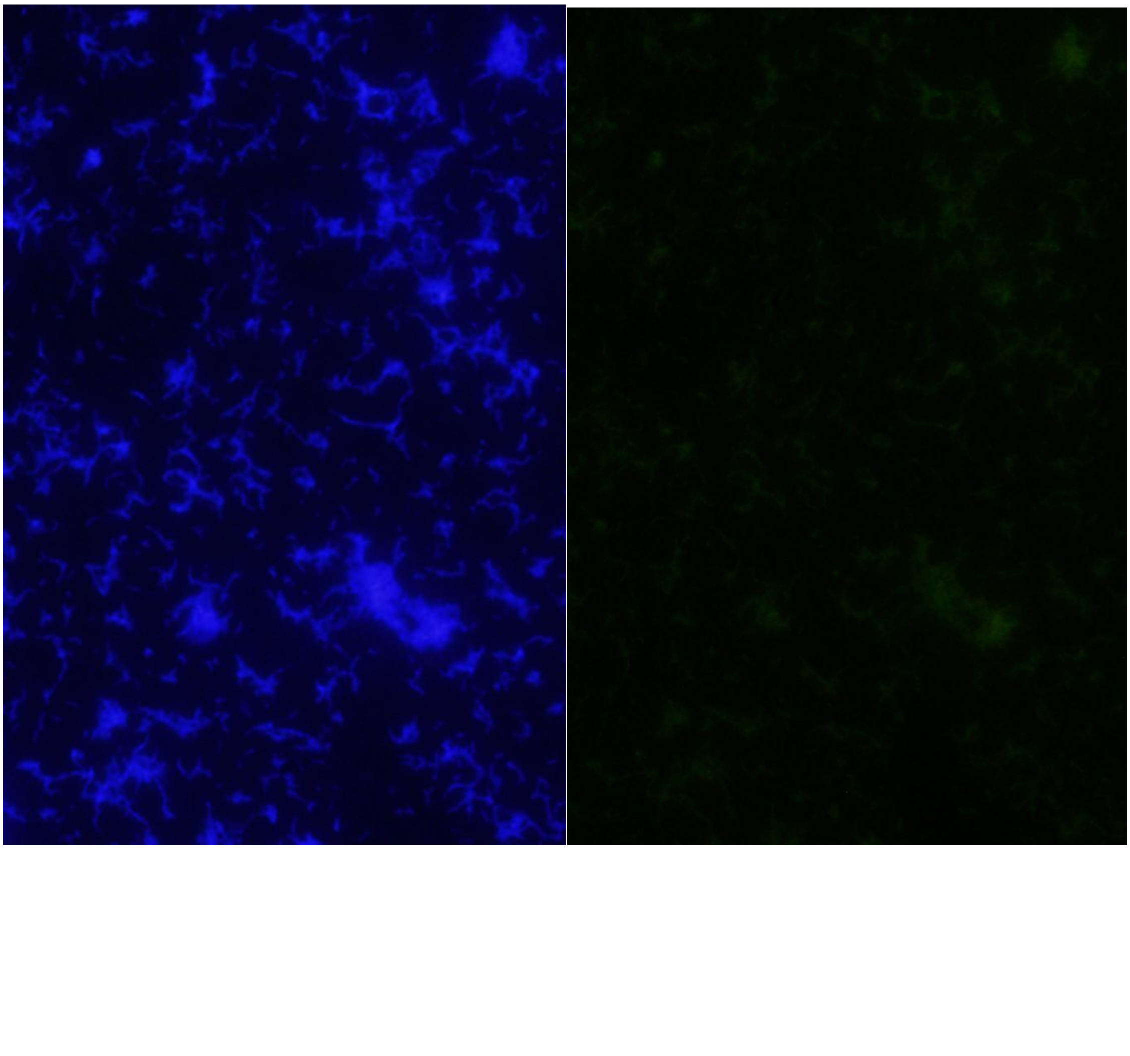

Brevibacterium ravenspurgense CCUG 42923

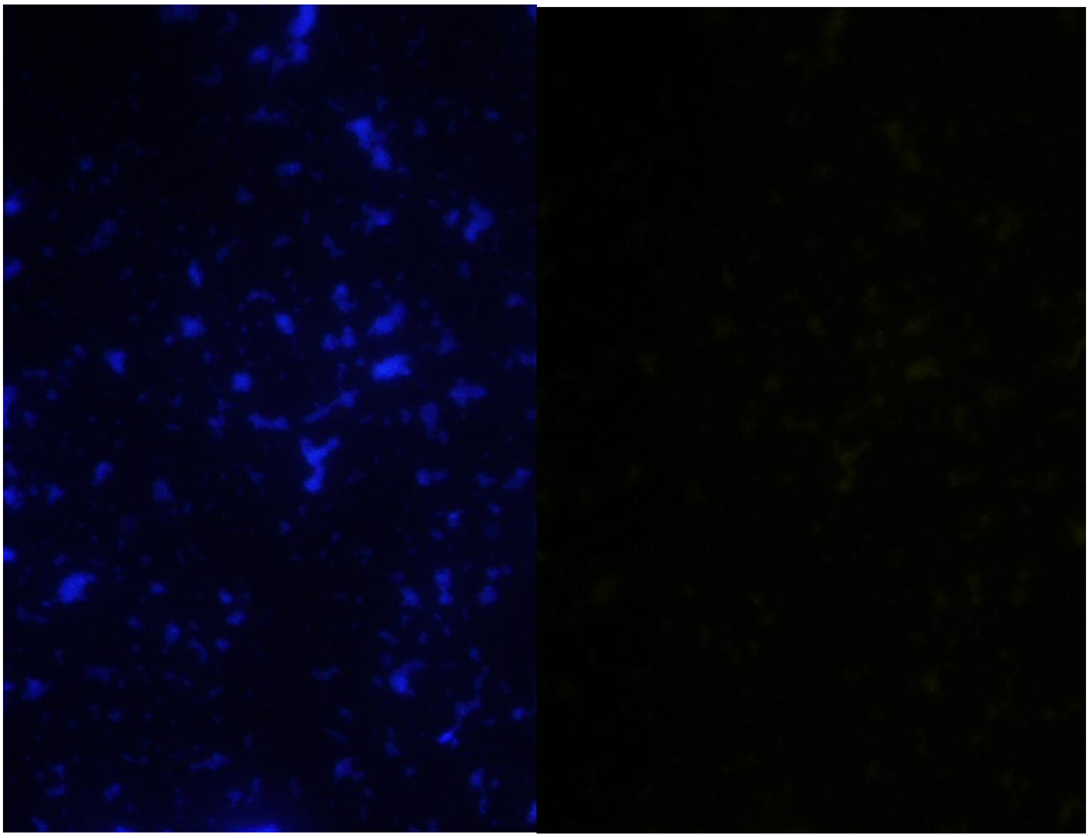

Corynebacterium tuscaniense UM137

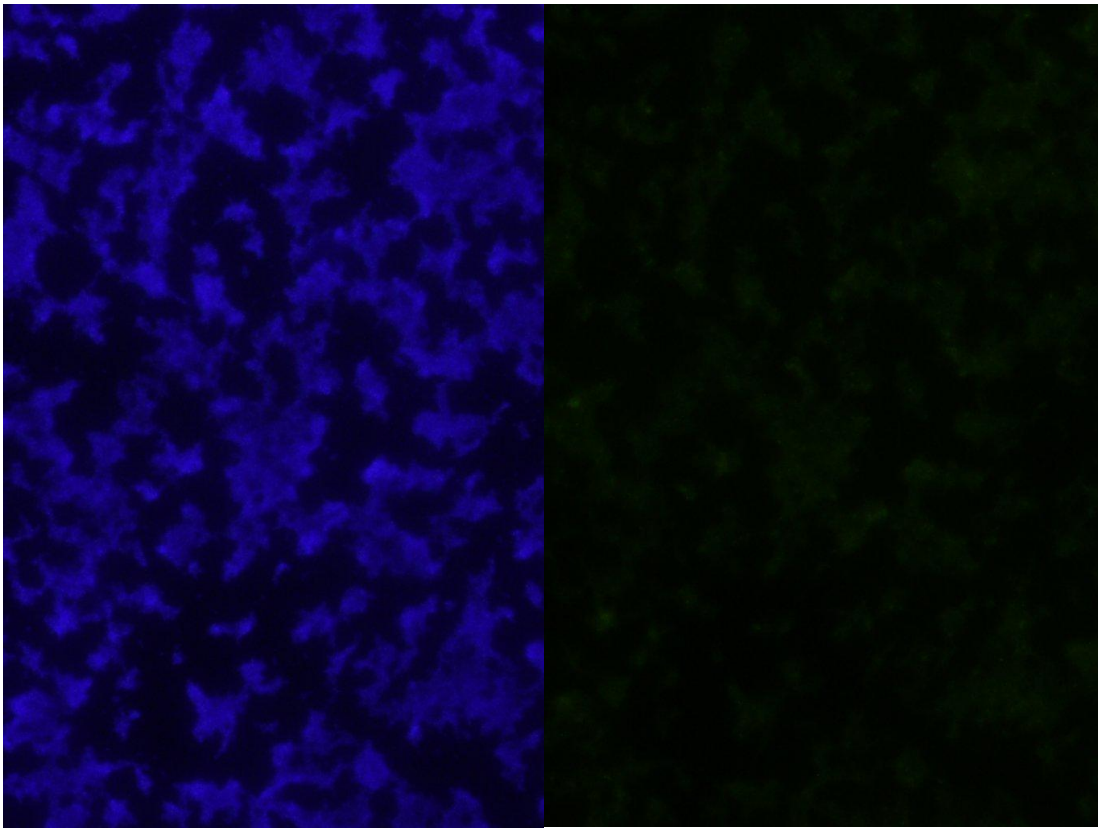

Enterococcus faecalis UM035

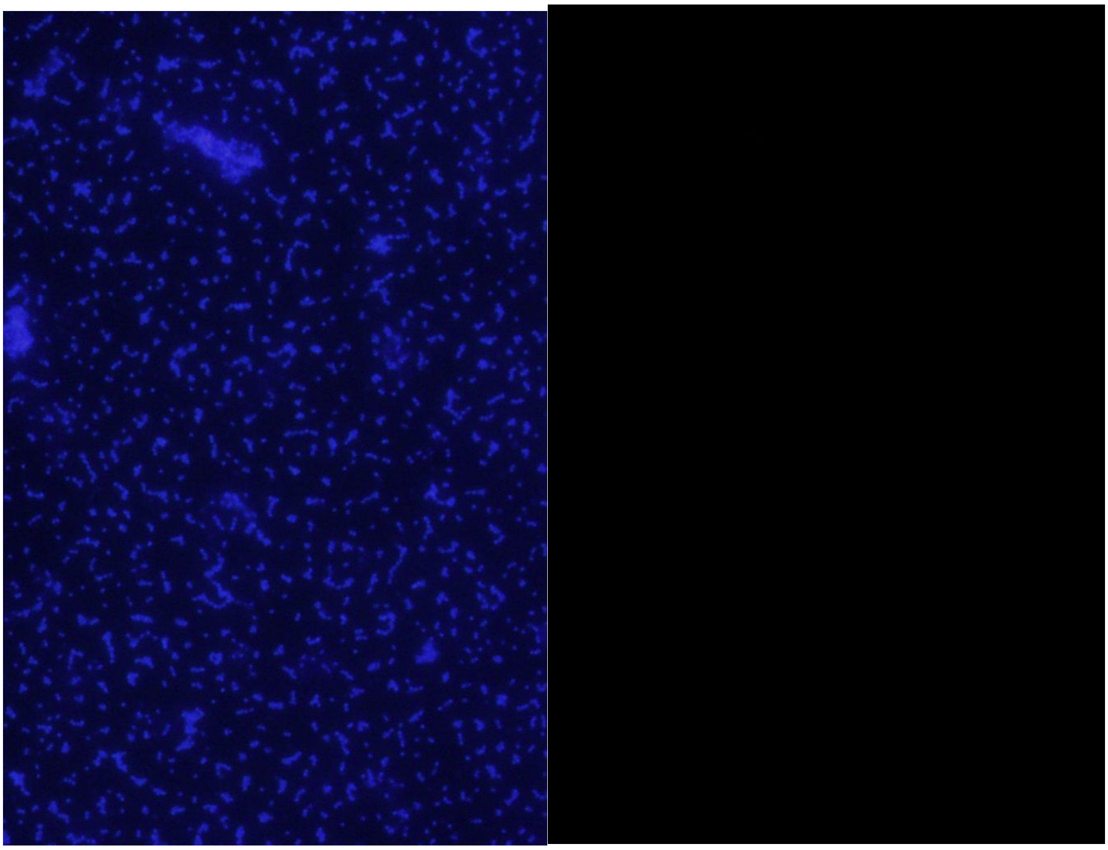

Escherichia coli UM056

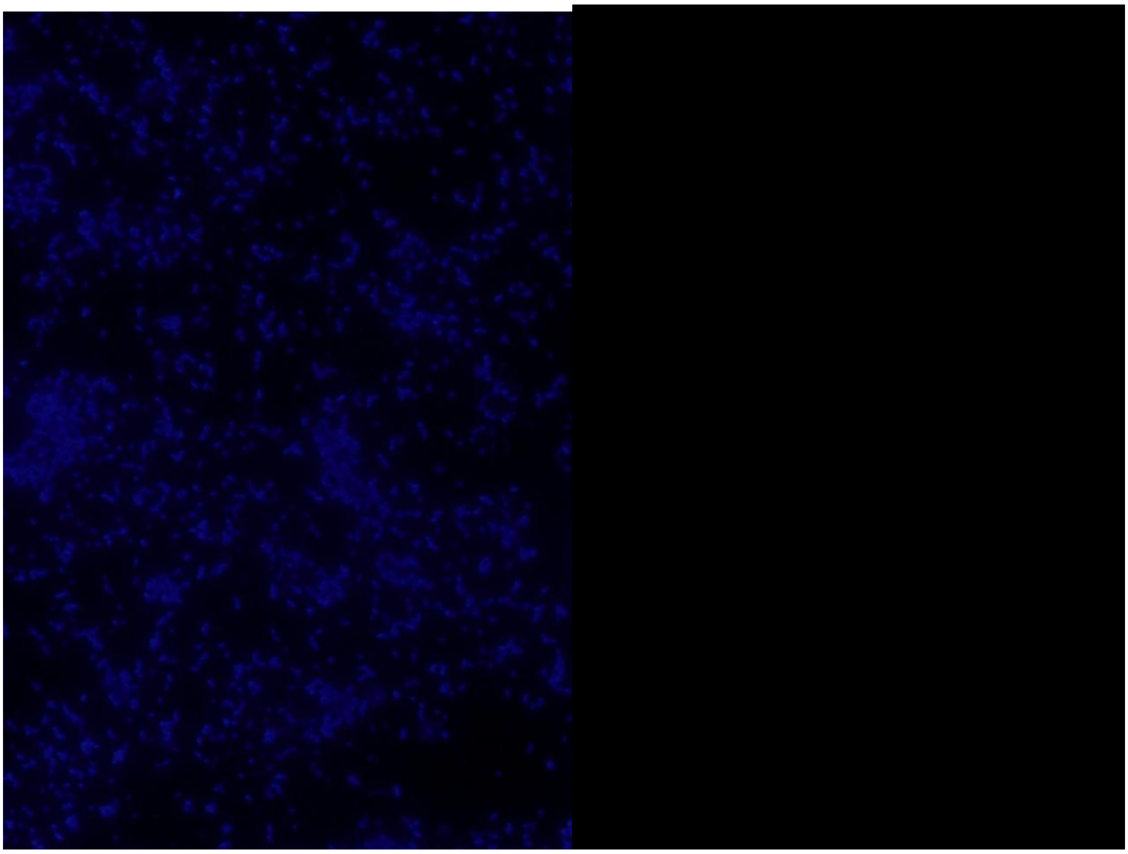

Fannyhessea vaginae ATCC BAA-55

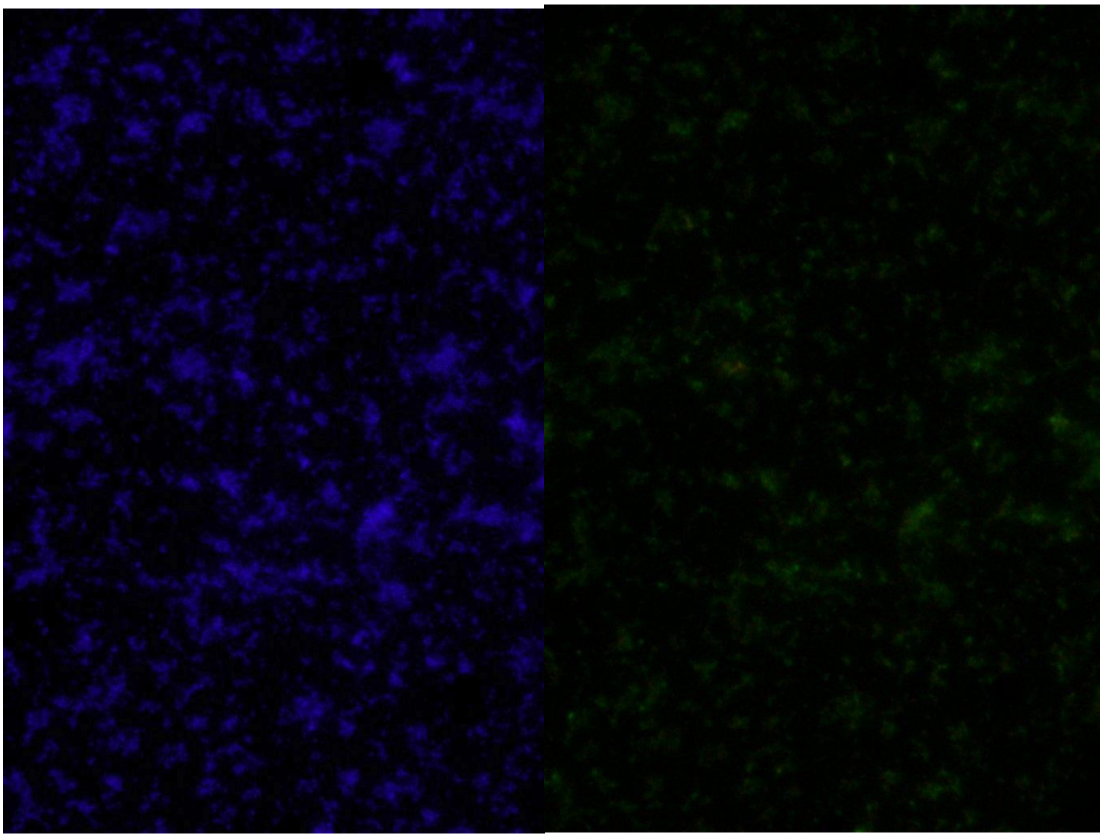

Gardnerella leopoldii UM034

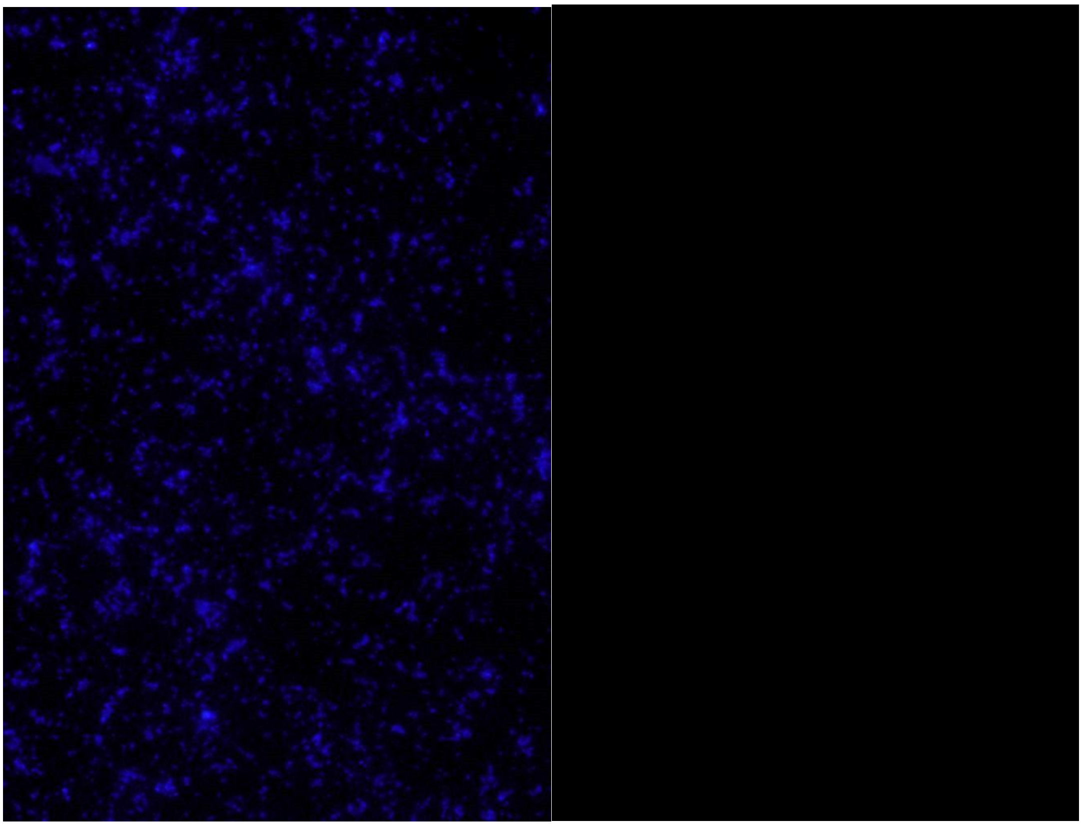

Gardnerella piotii UM035

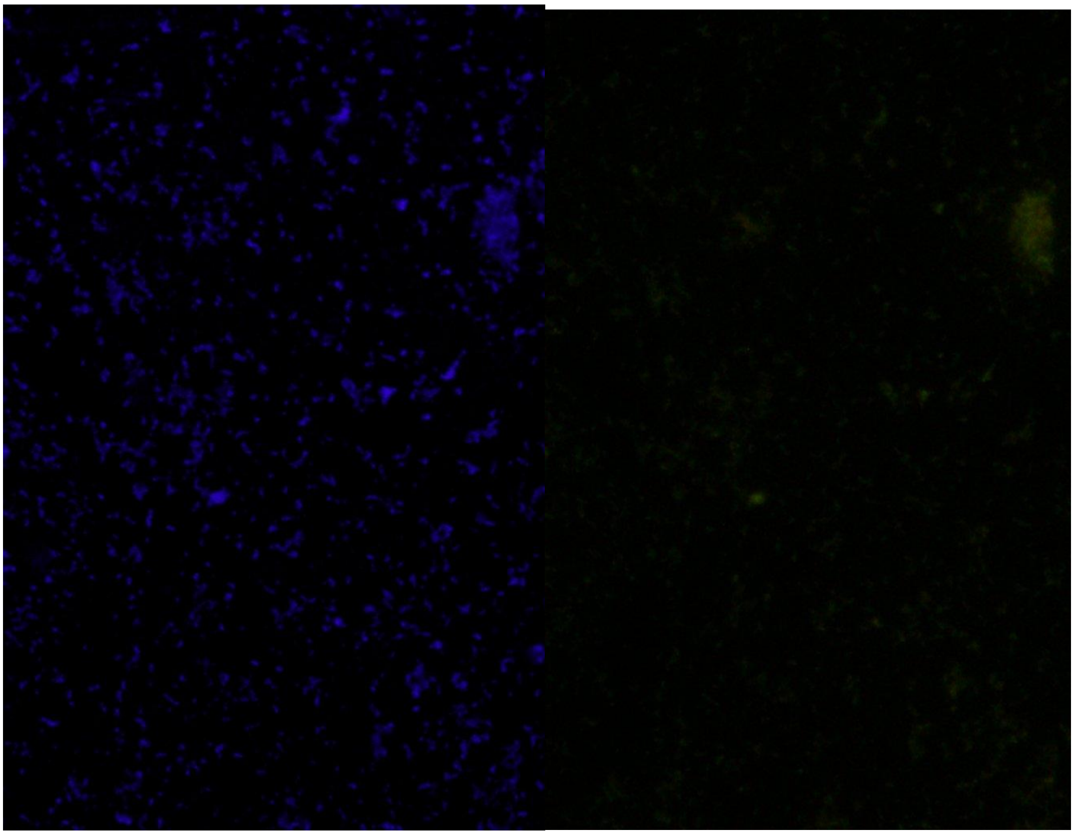

Gardnerella swidsinskii UM094

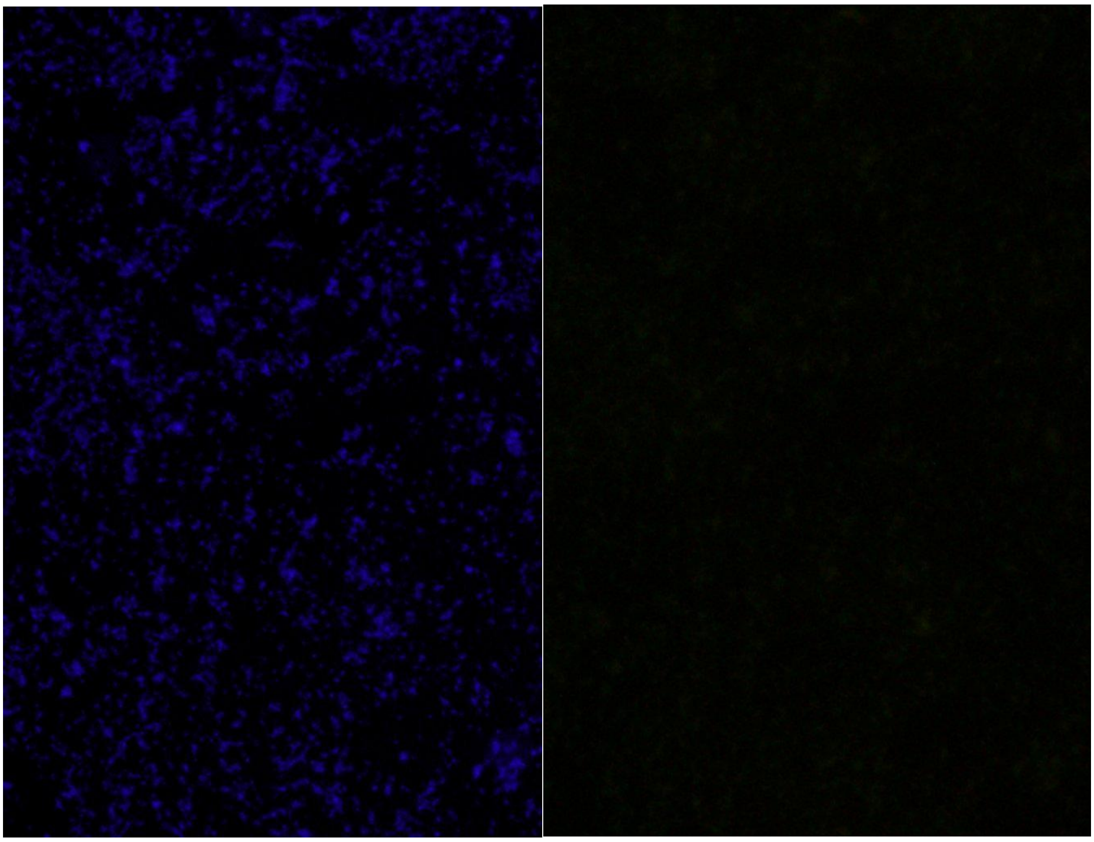

Gardnerella vaginalis ATCC 14018

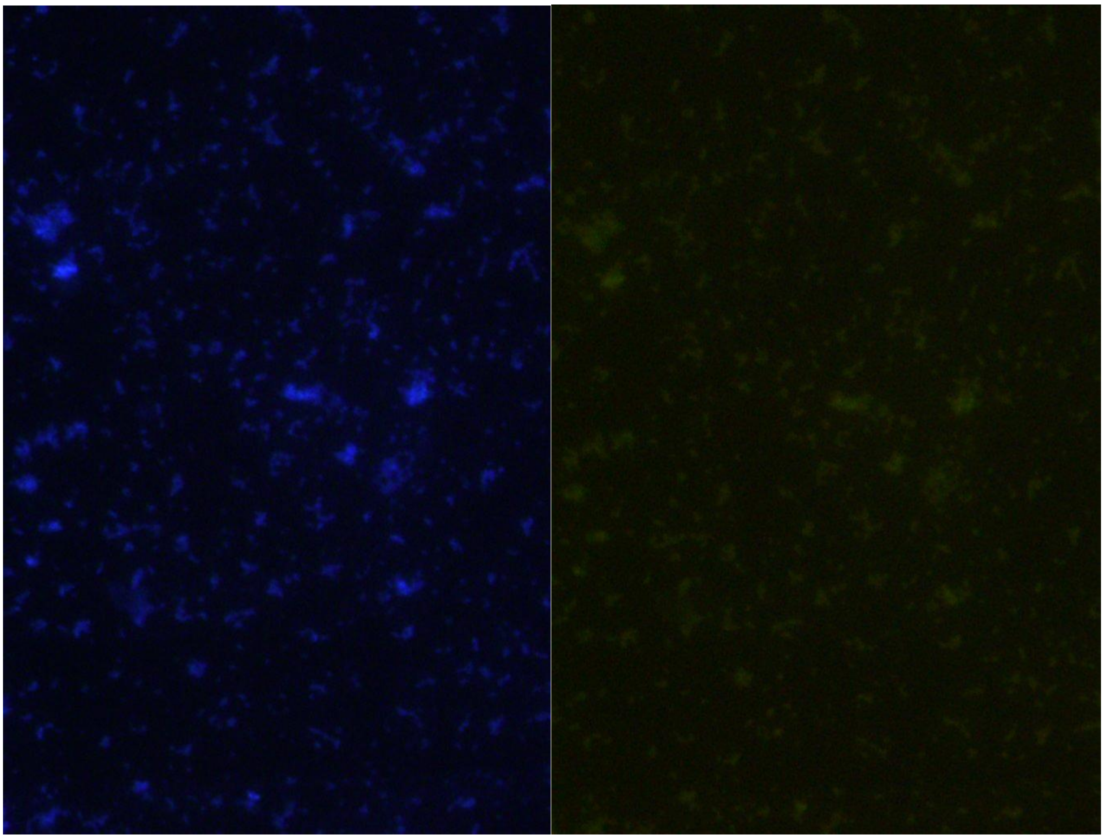

Gemella haemolysans UM034

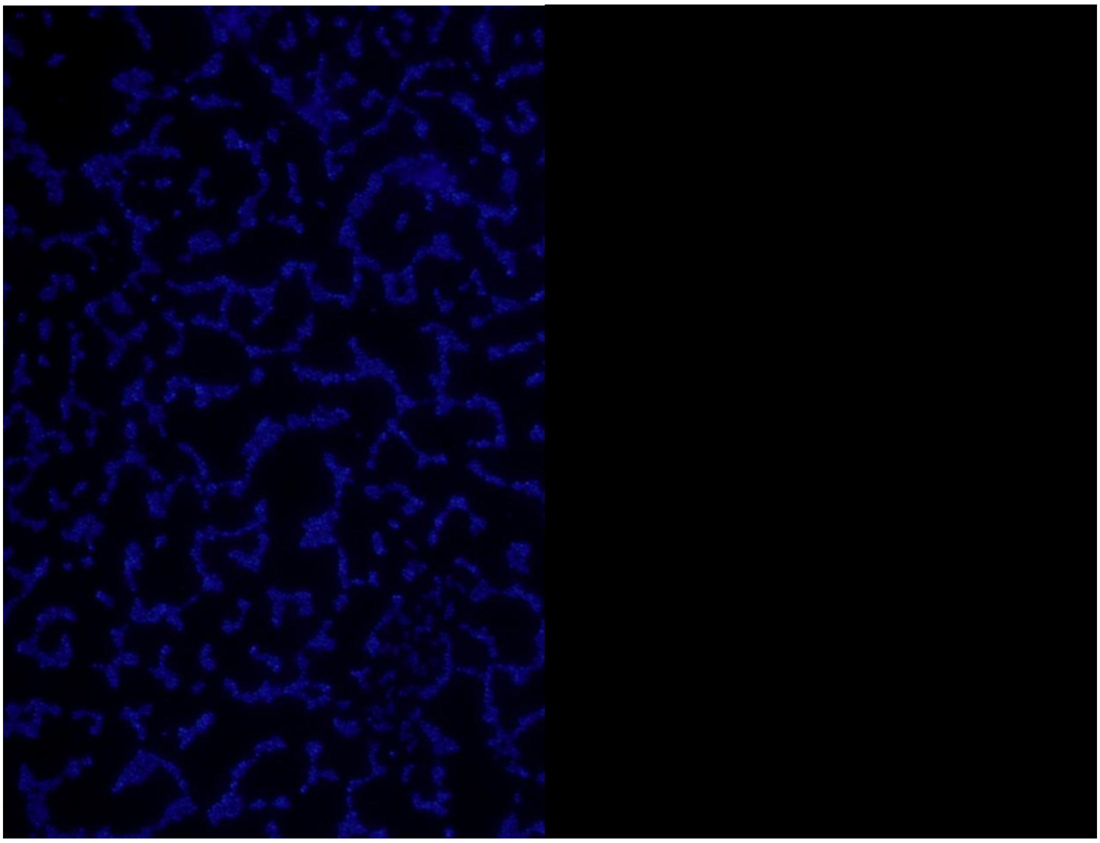

Lactobacillus crispatus EX533959VCO6

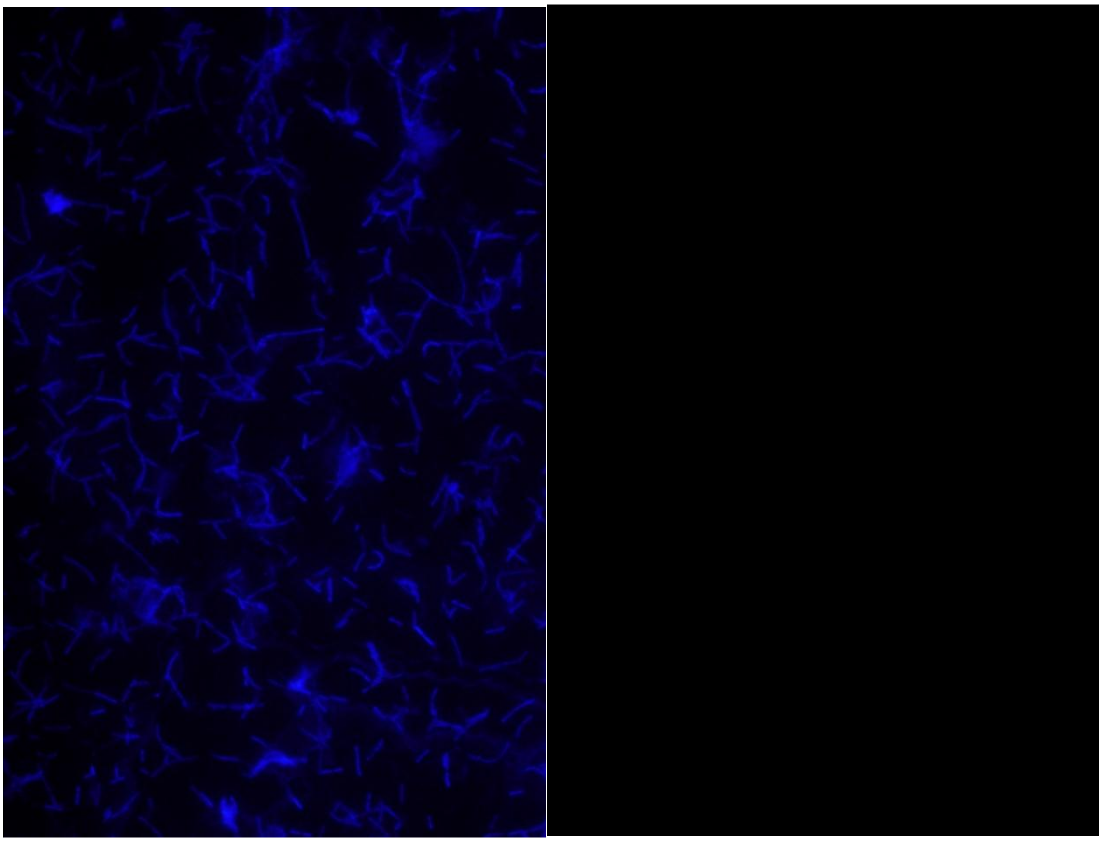

Lactobacillus gasseri ATCC 9857

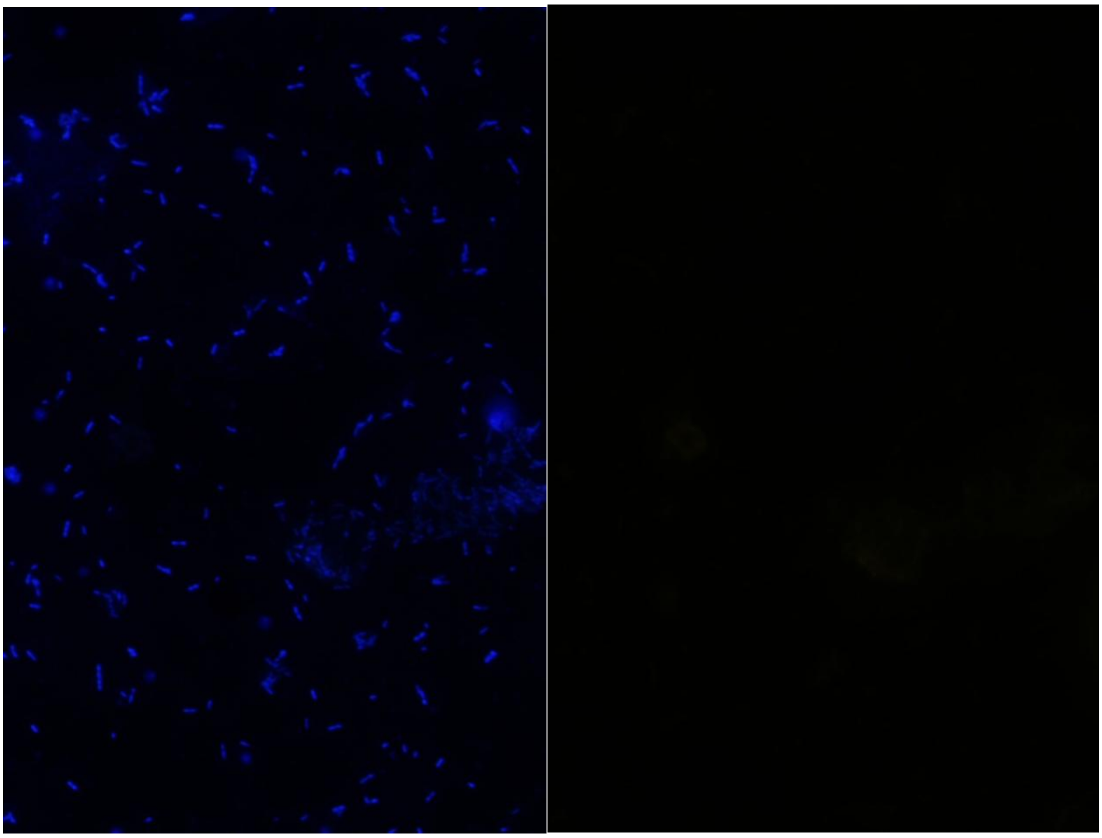

Lactobacillus iners ATCC 55195

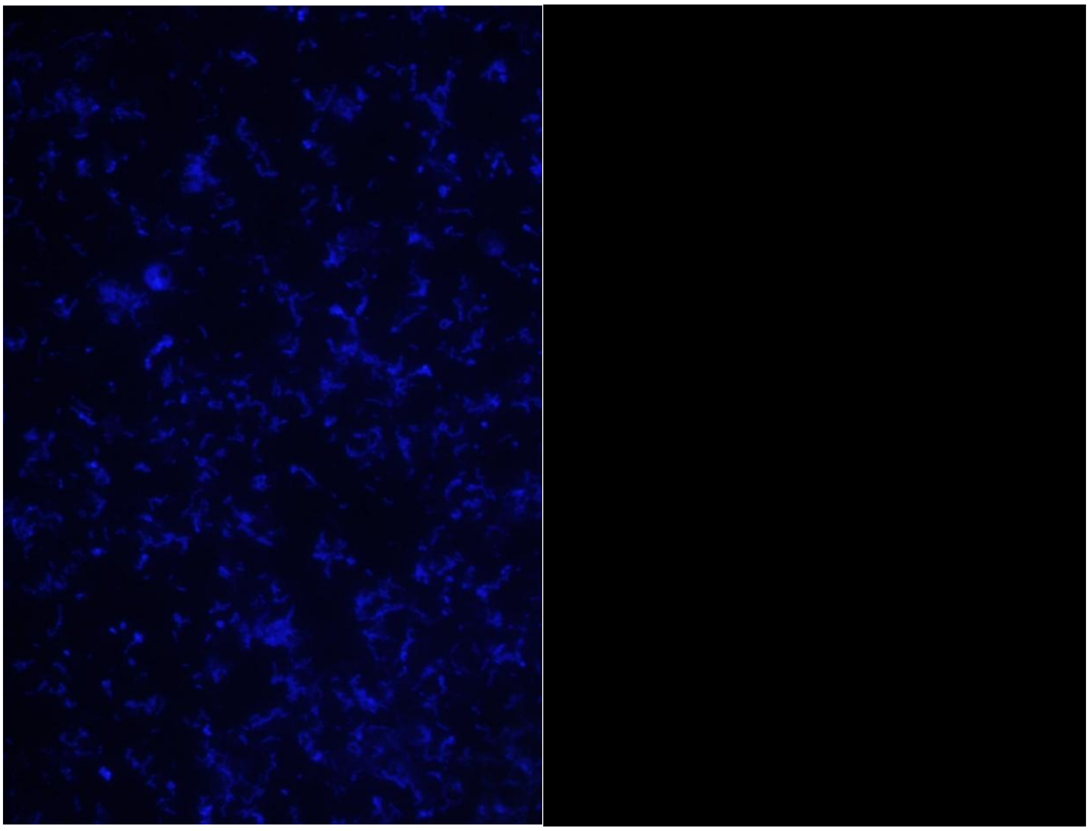

Lactobacillus rhamnosus CECT 288

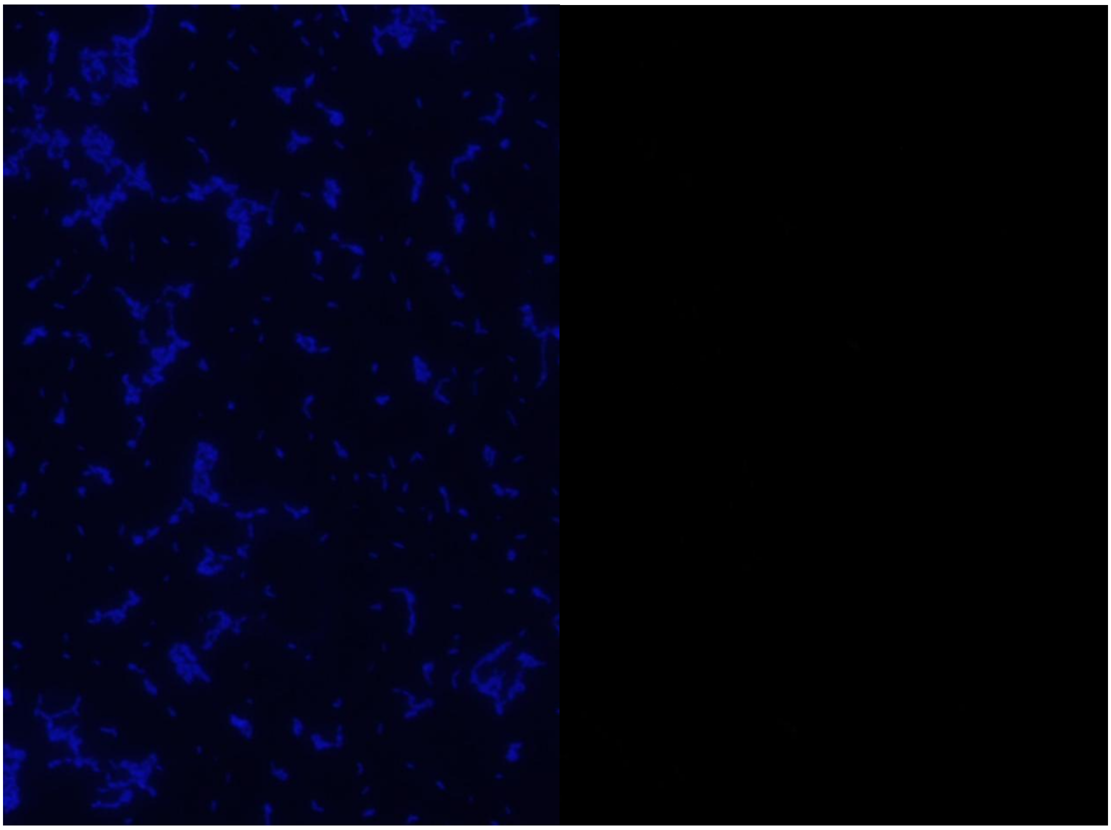

Lactobacillus vaginalis UM062

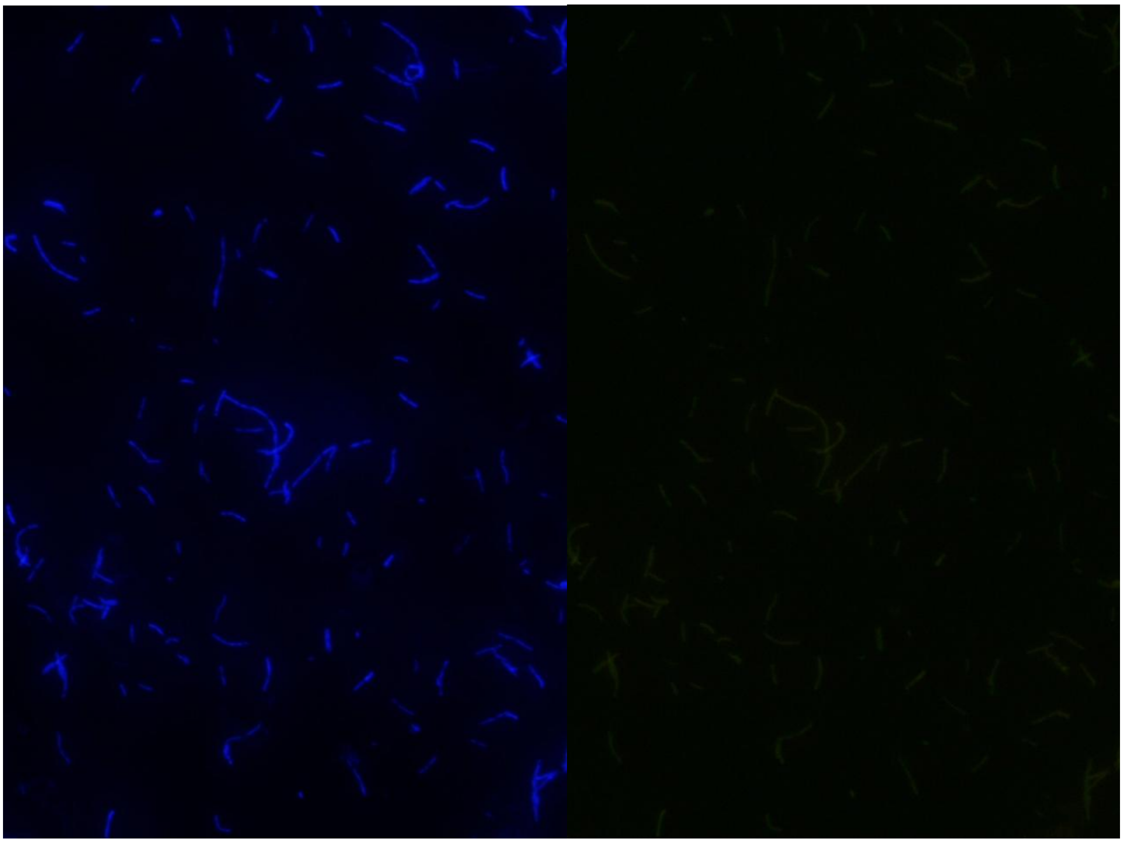

Megasphaera micronuciformis CCUG 45952T

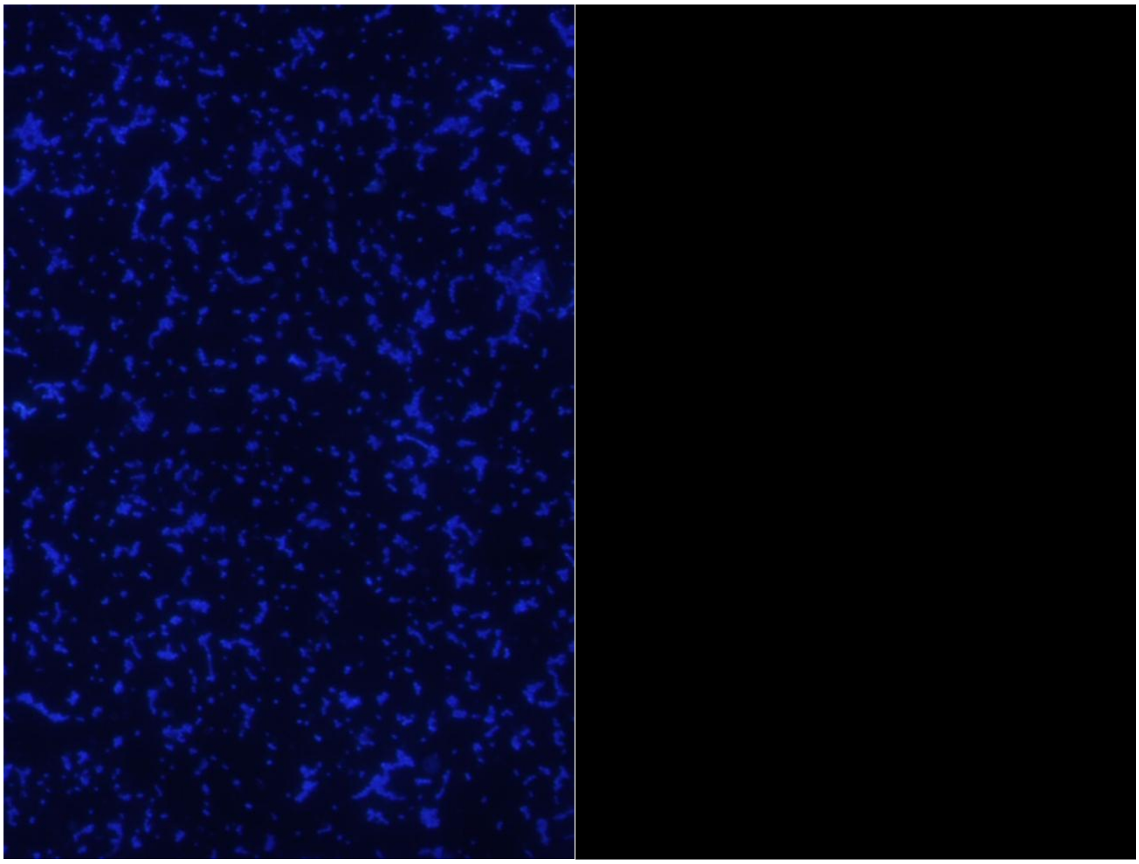

Mobiluncus curtisii ATCC 35241

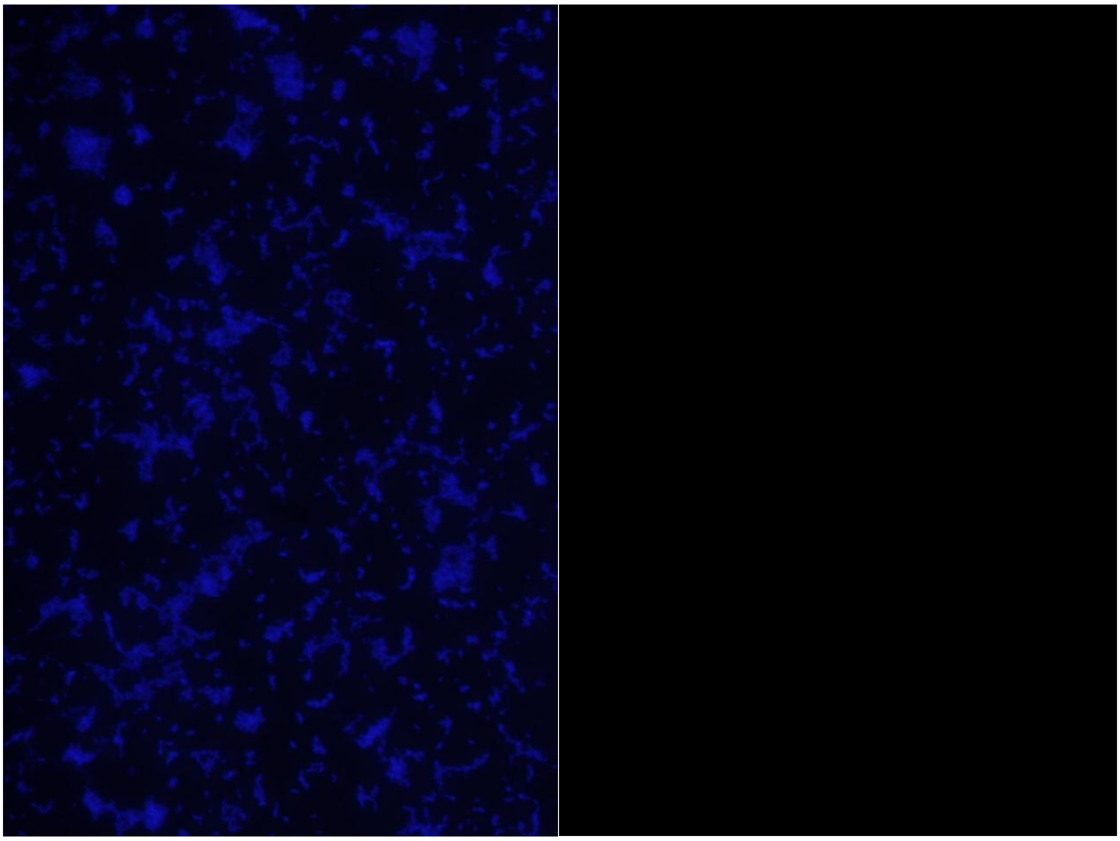

Mobiluncus mulieris ATCC 35239

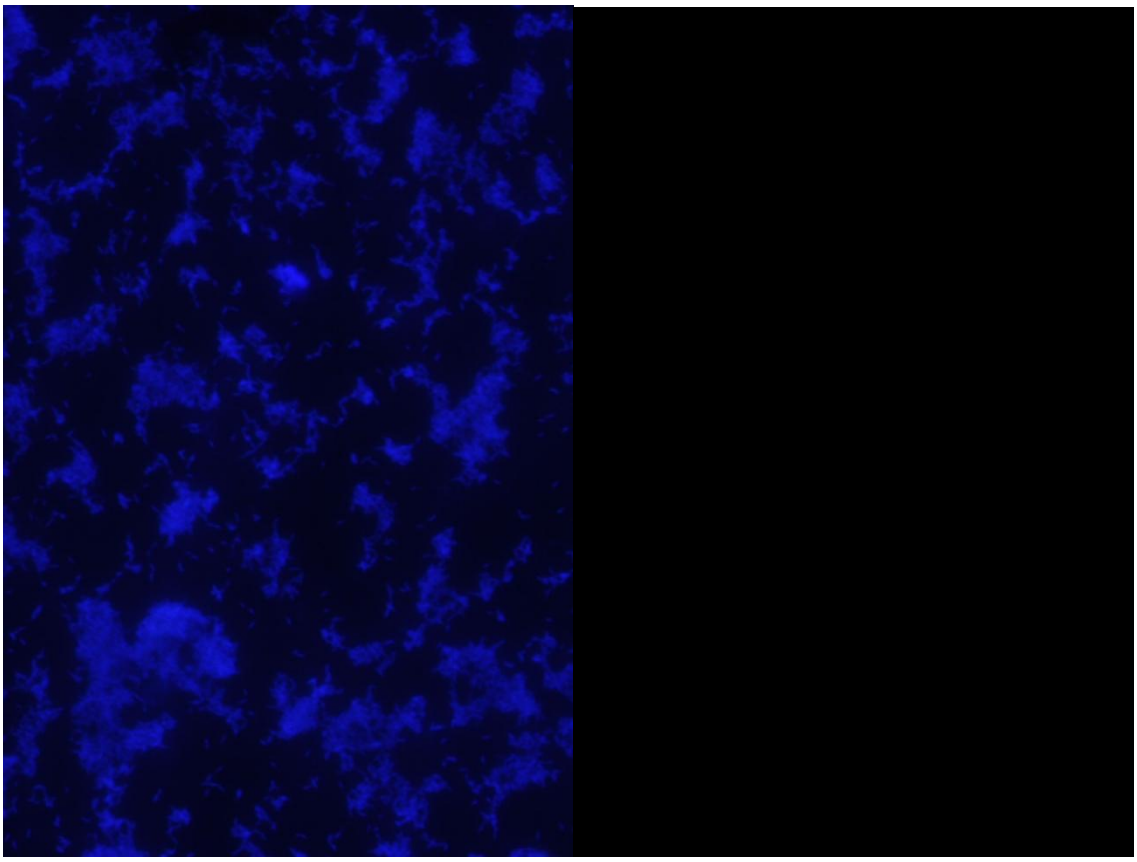

Mycoplasma hominis UM054

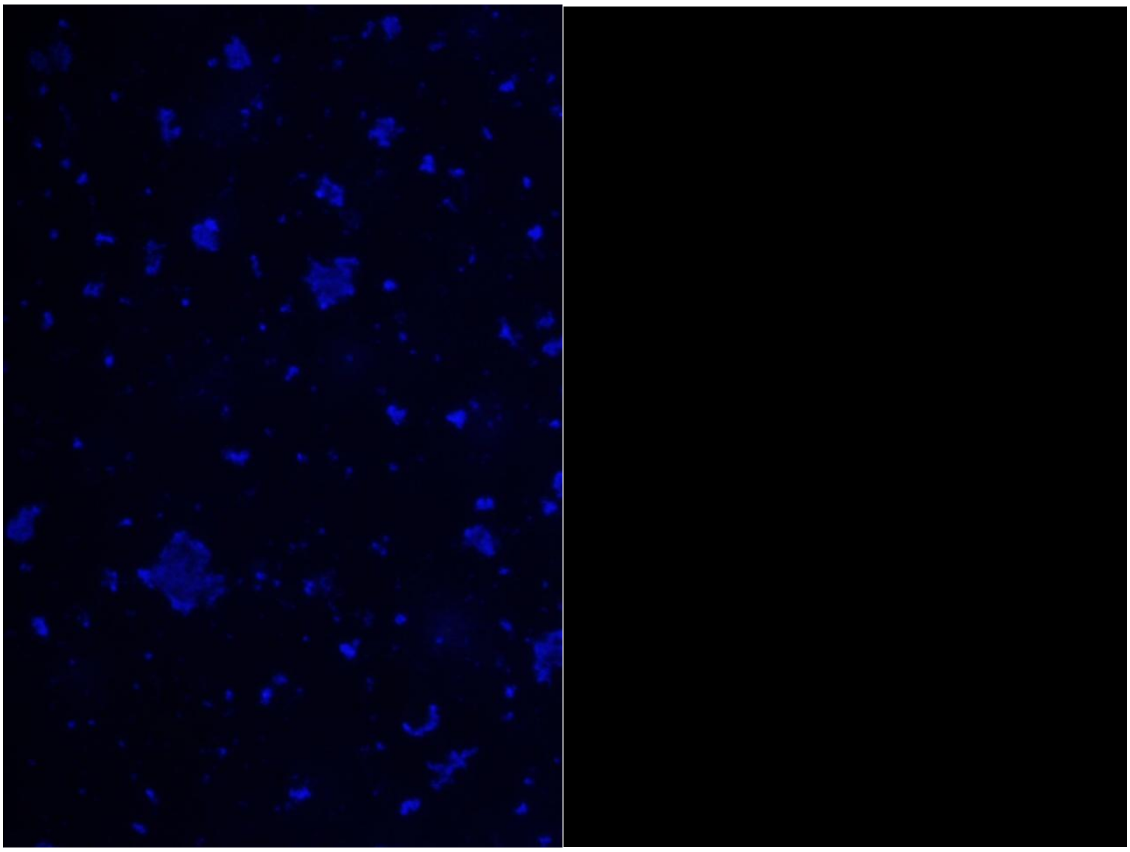

Neisseria gonorrhoeae CCUG 13281

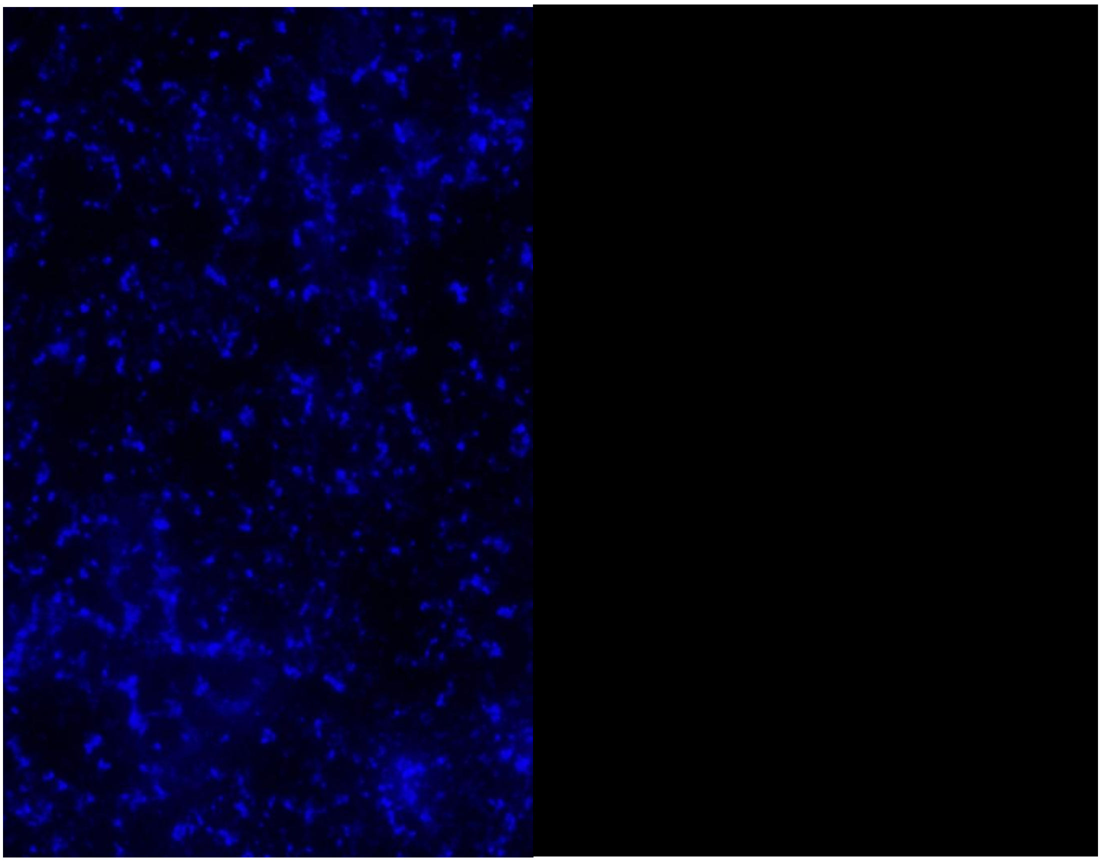

Nosocomiicoccus ampullae UM121

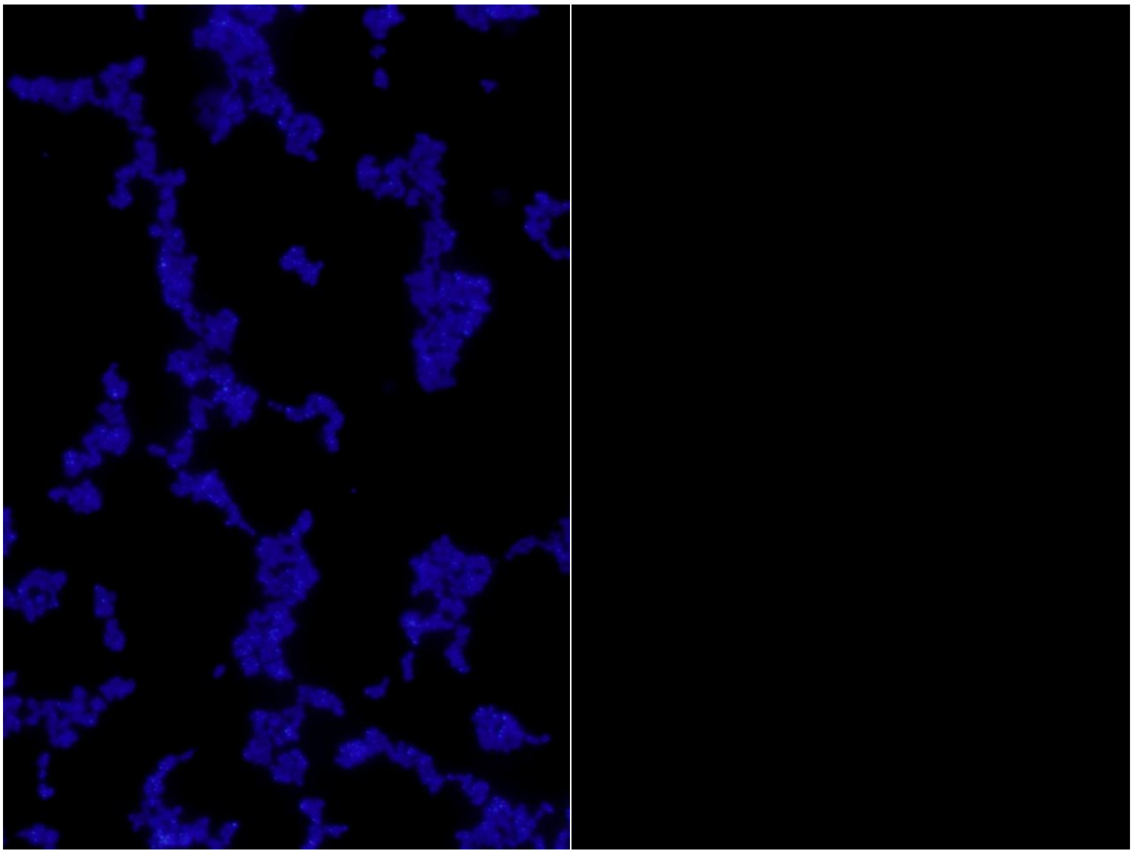

Peptostreptococcus anaerobius ATCC 27337

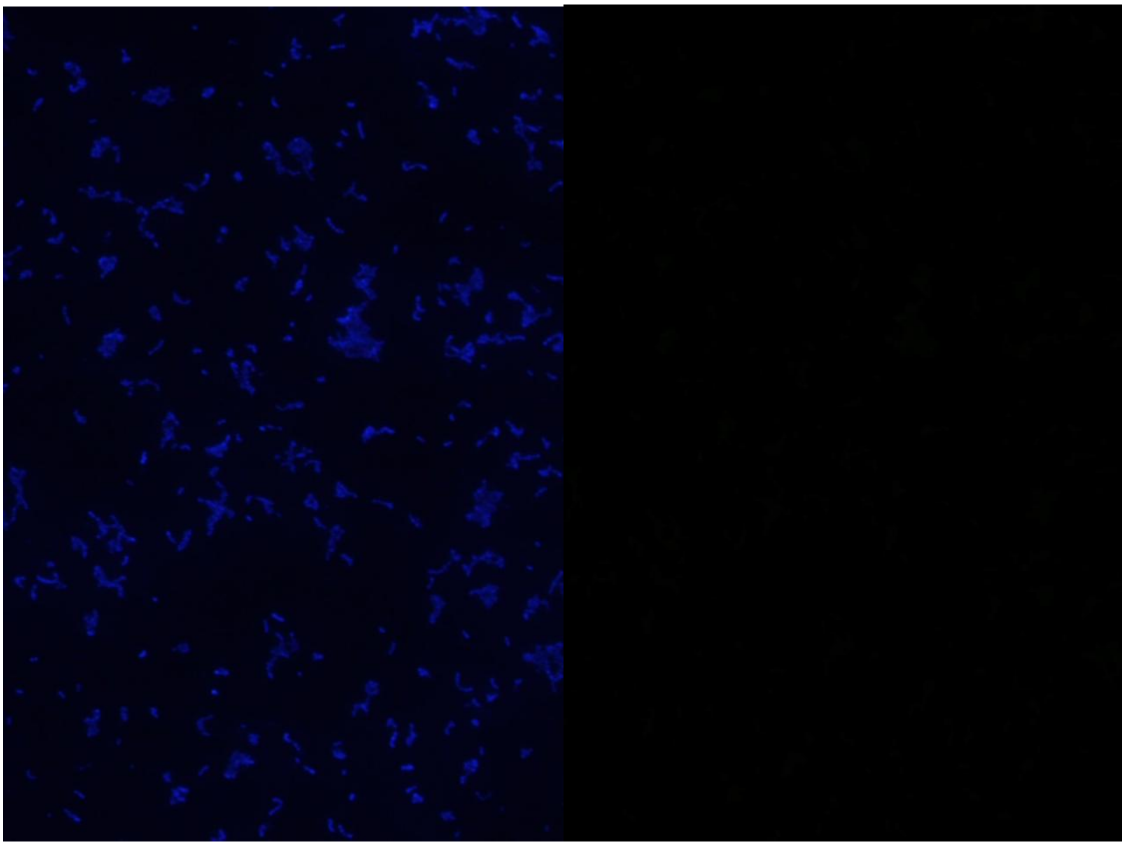

Porphyromonas asaccharolytica CCUG 7834T

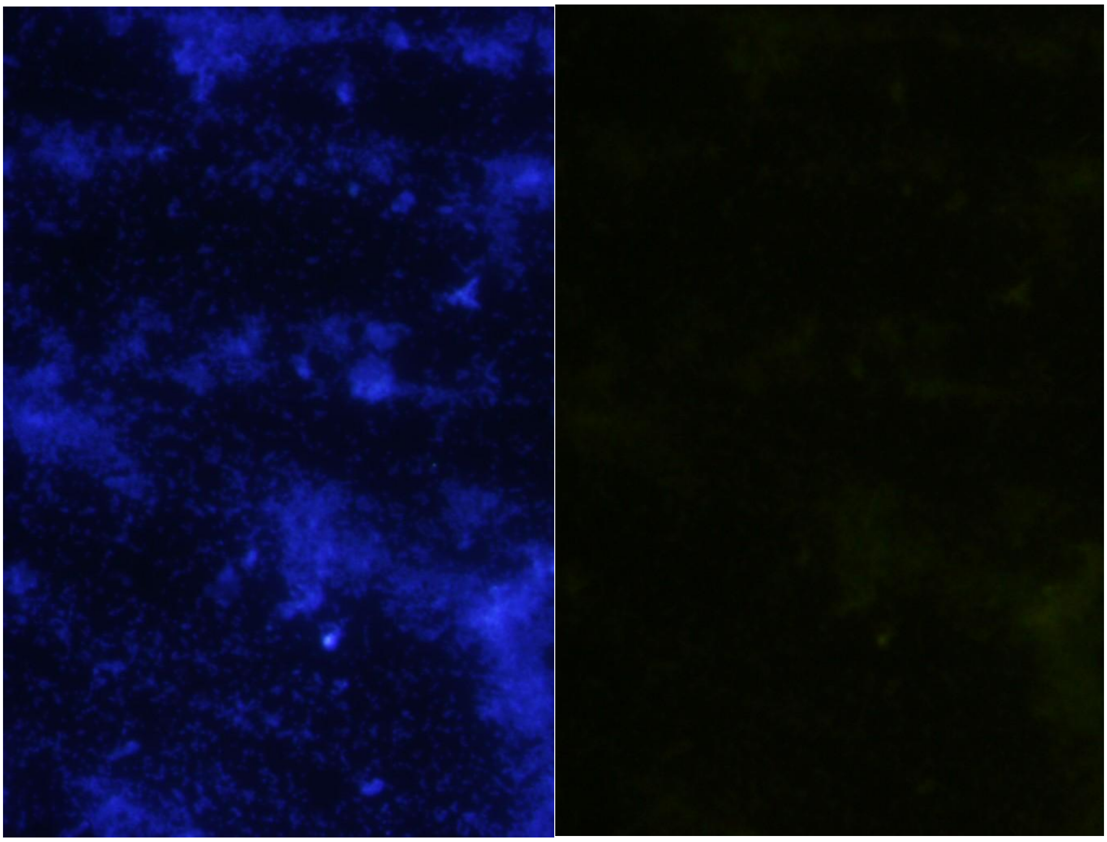

Prevotella amnii CCUG 53648

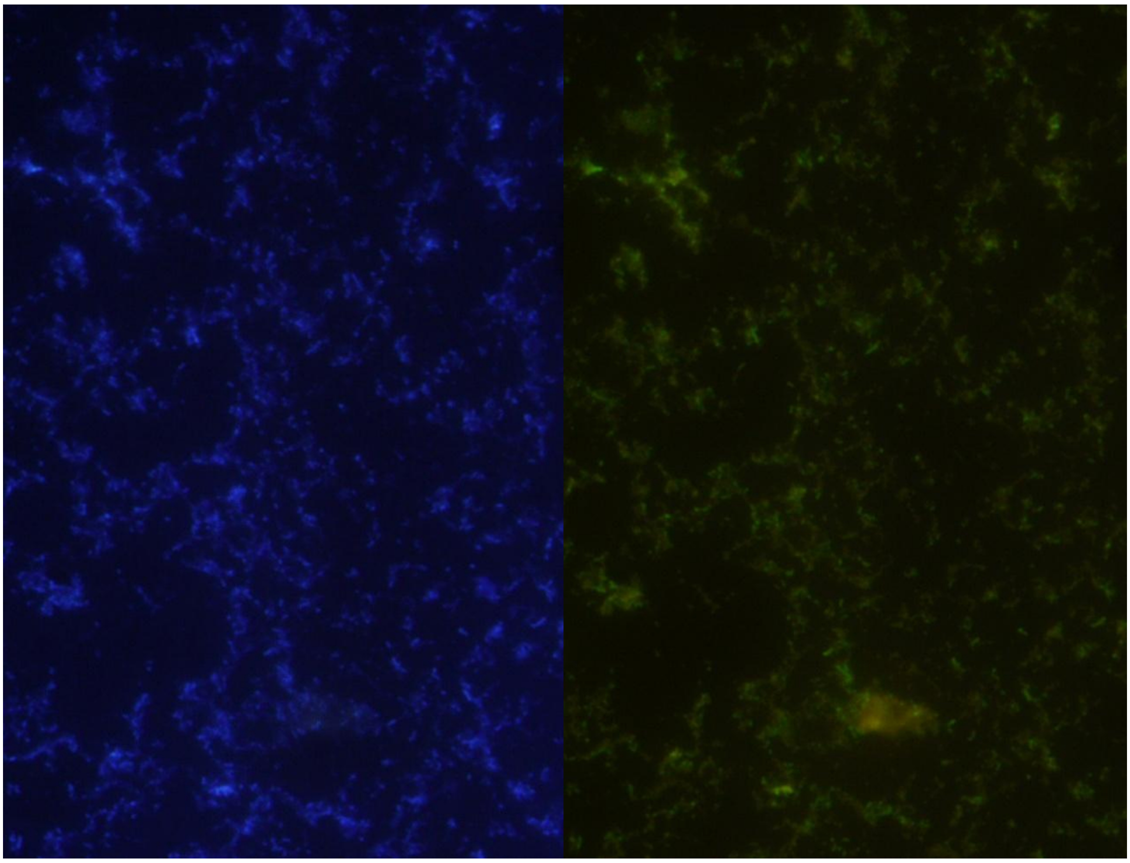

Prevotella bivia ATCC 29303

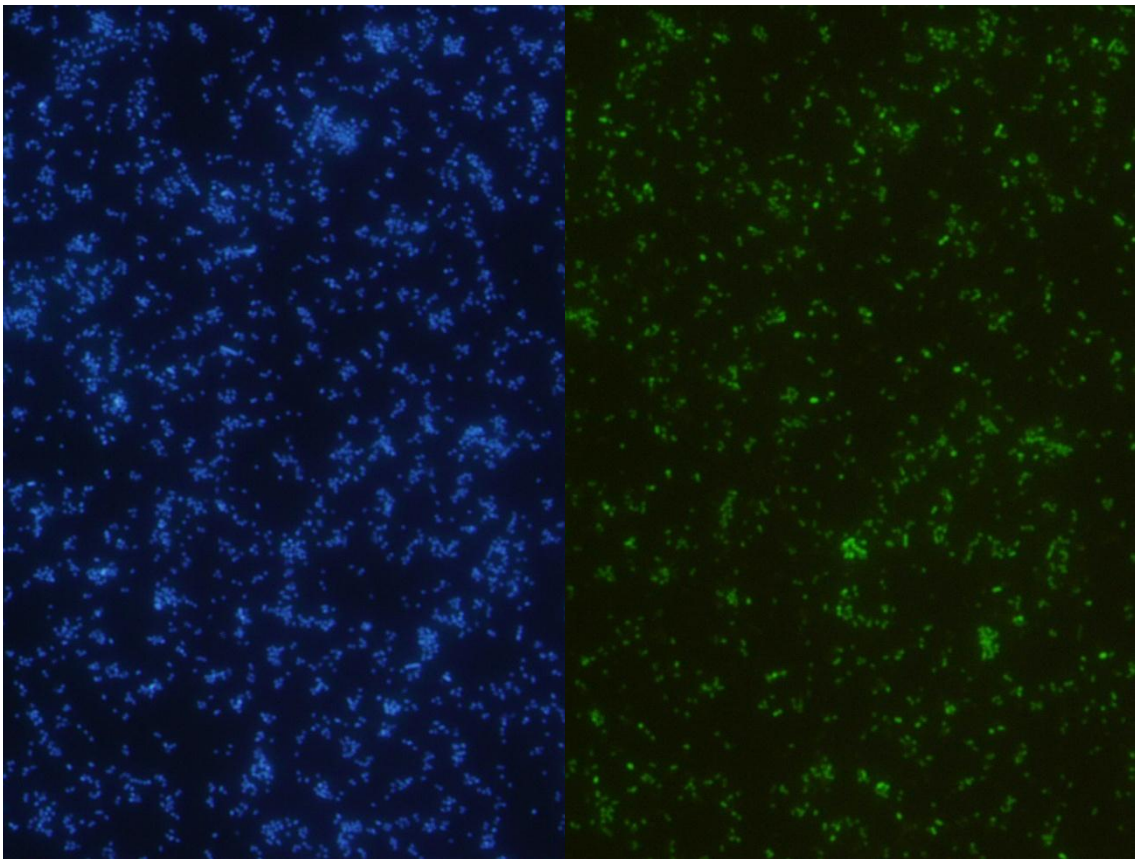

Prevotella bivia CCUG 33360

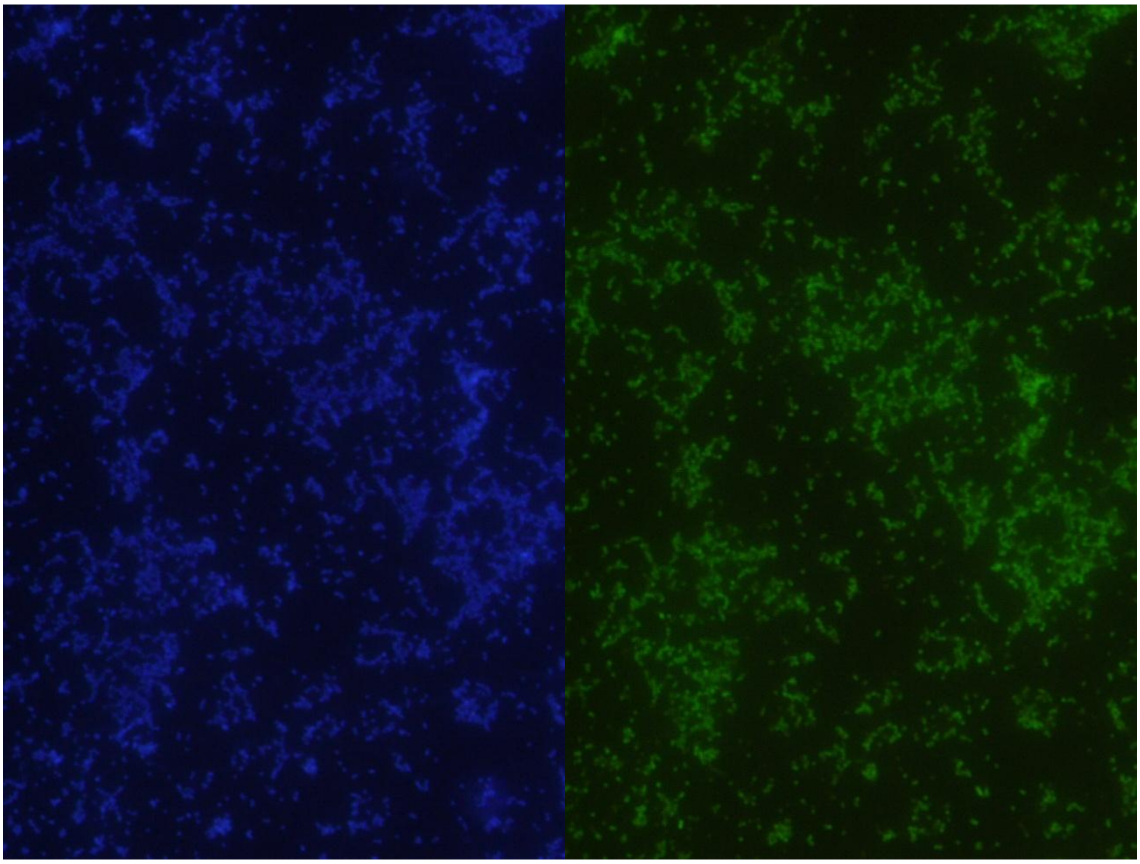

Prevotella bivia CCUG 34046

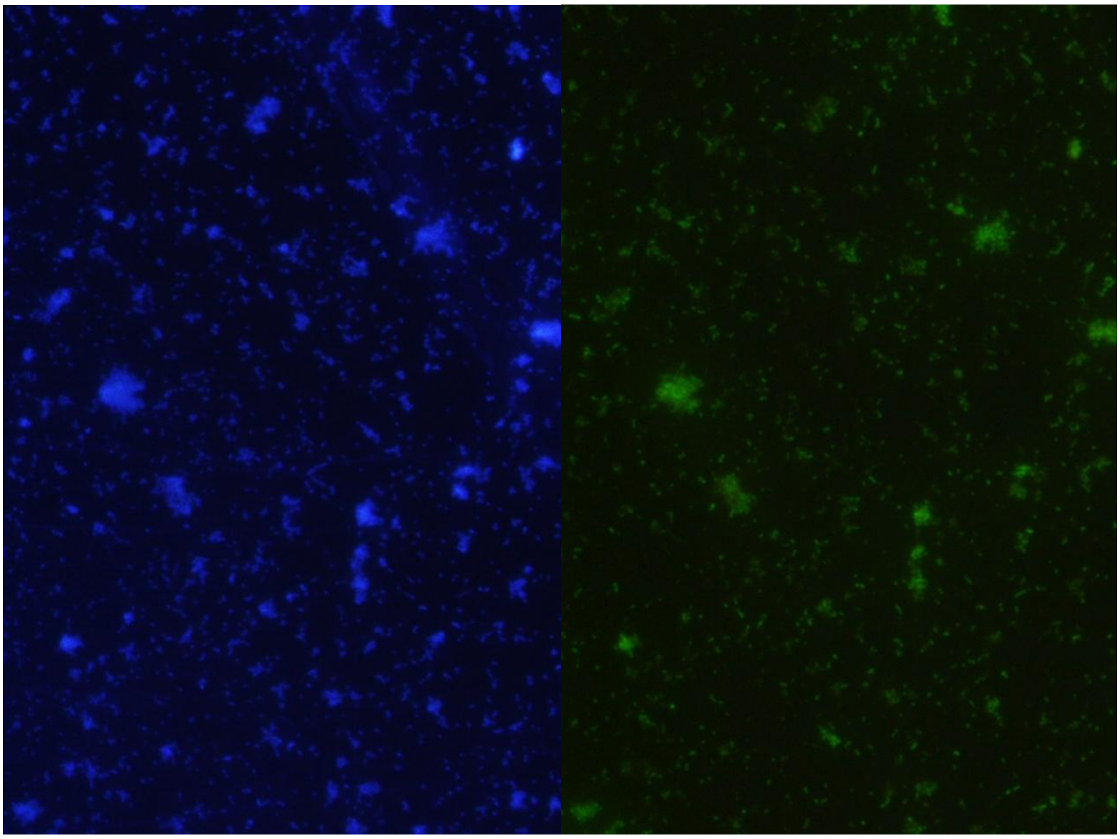

Prevotella bivia CCUG 44195

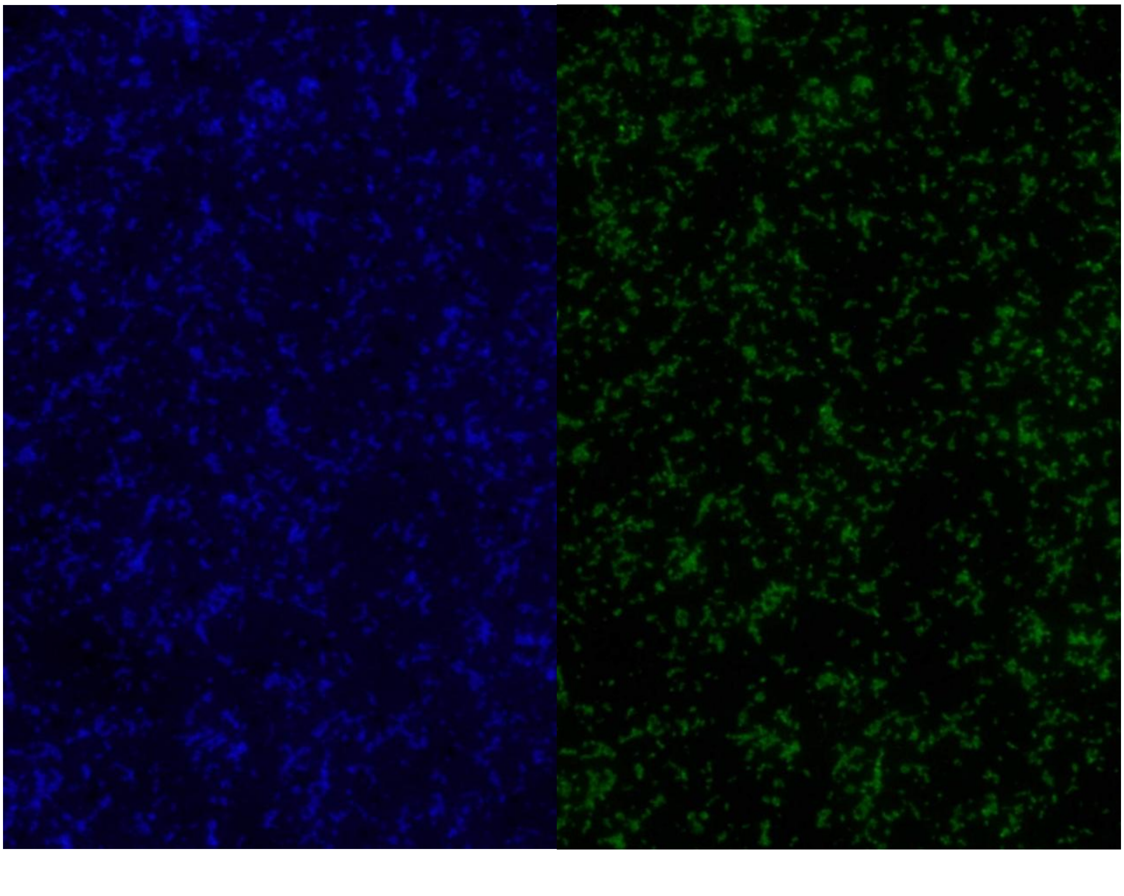

Prevotella bivia   CCUG 59496

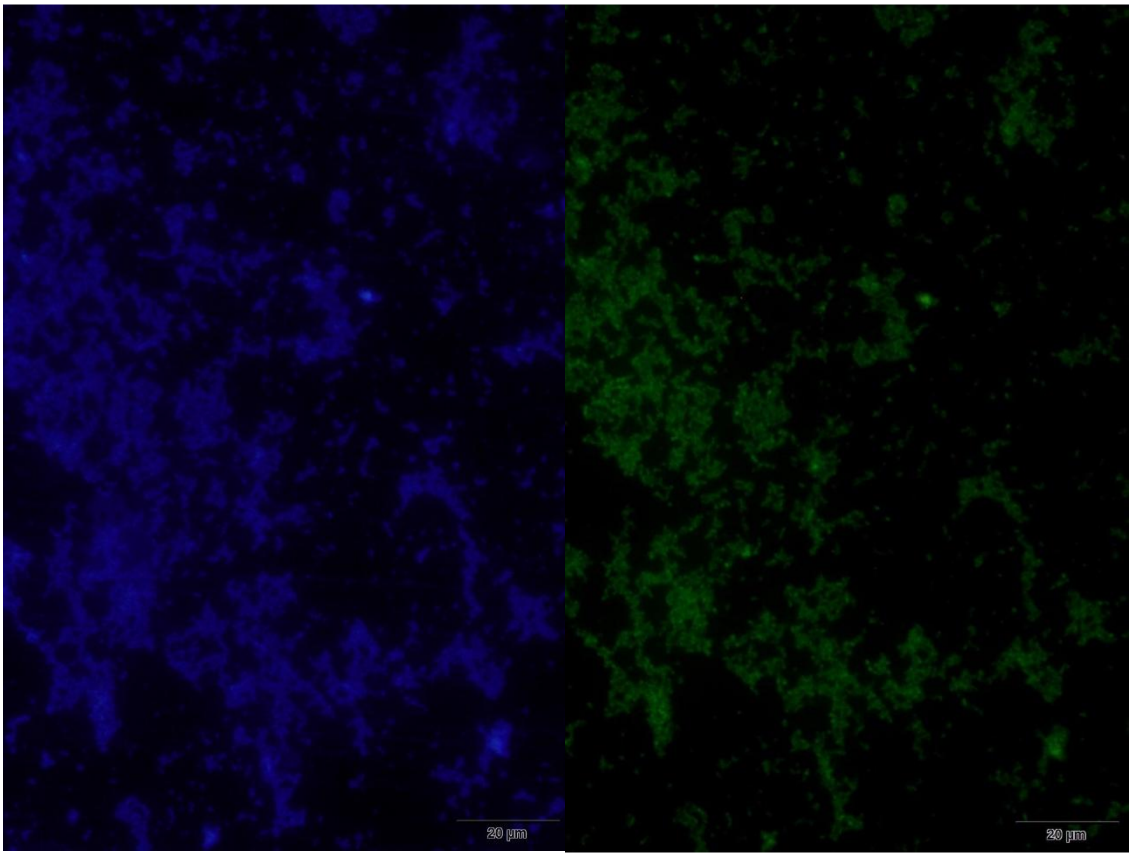

Prevotella brunnea   CCUG 72809

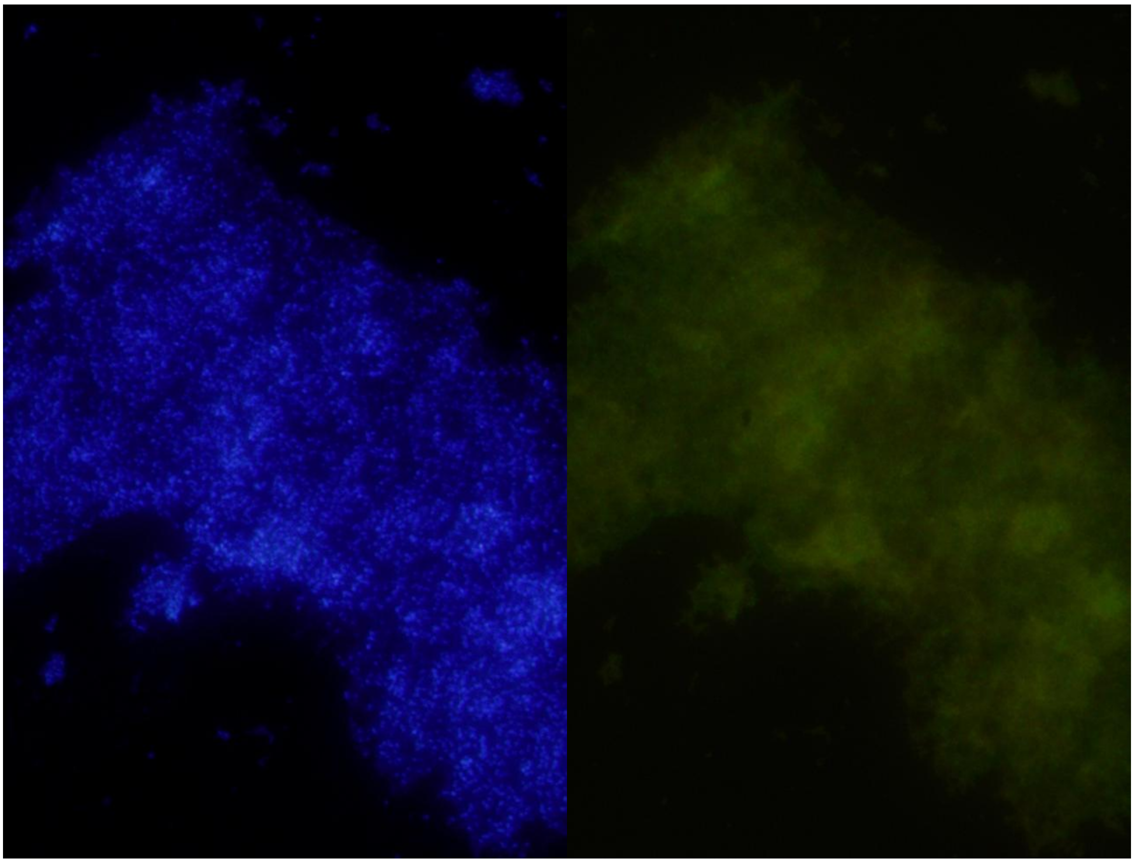

Prevotella buccalis   CCUG 44127

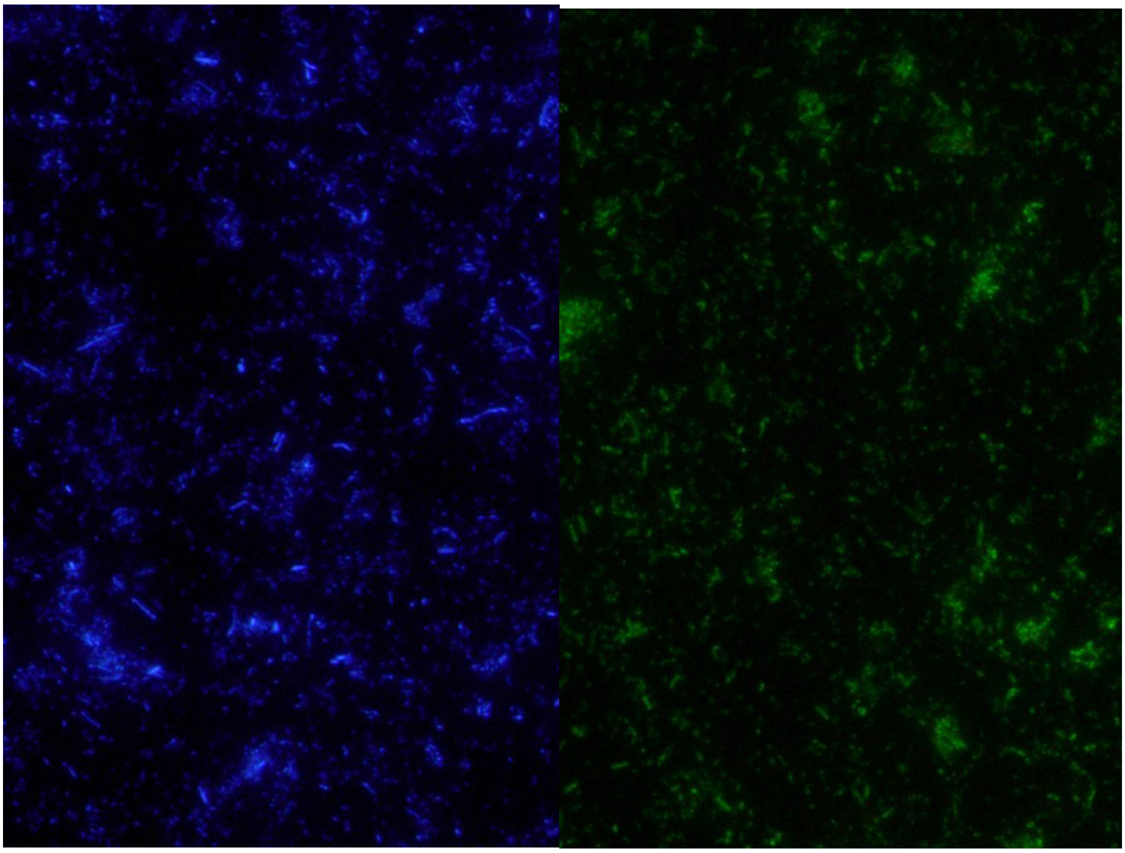

Prevotella copri   CCUG 58058T

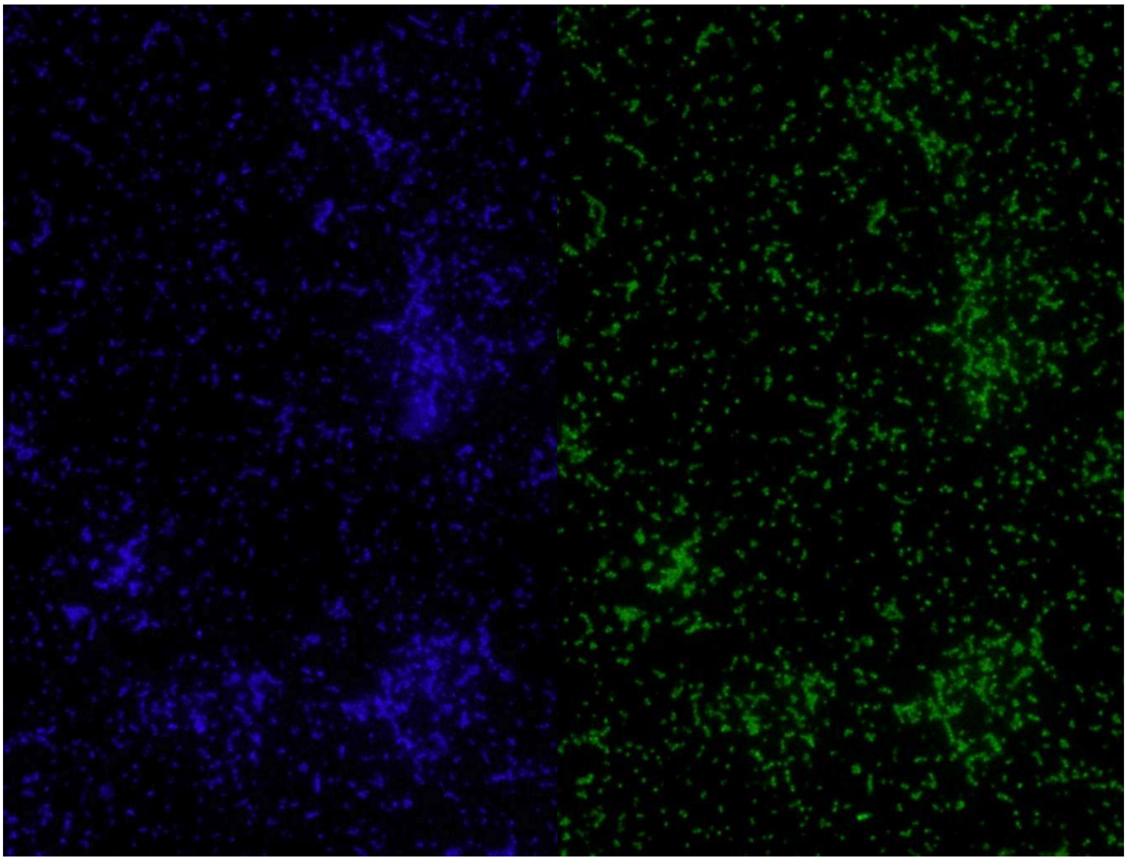

Prevotella corporis CCUG15404

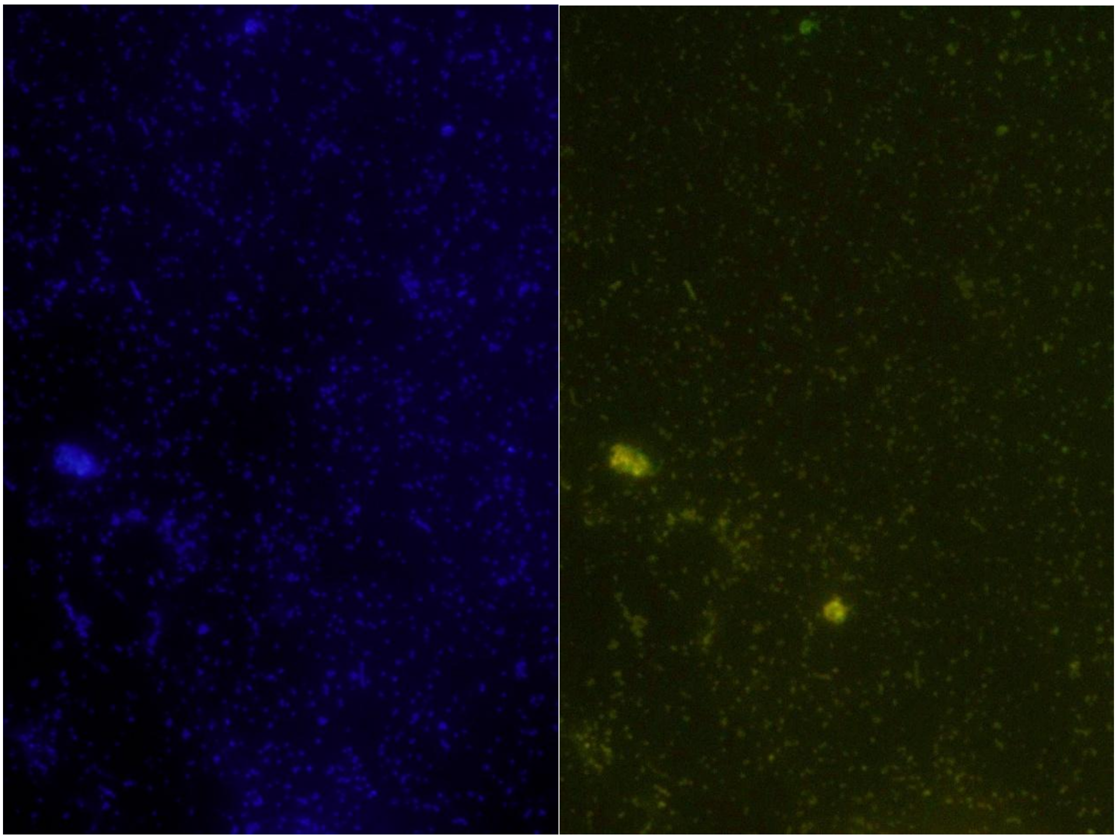

Prevotella dentalis CCUG48288

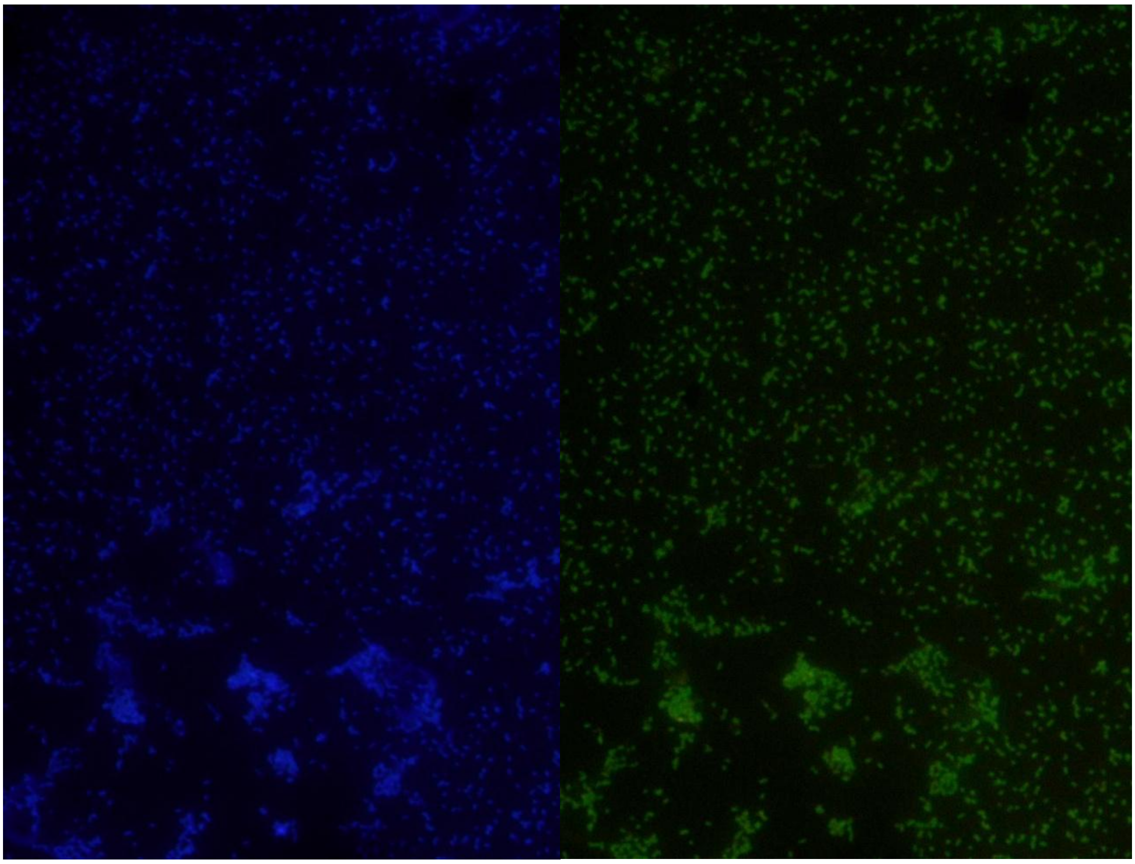

Prevotella denticola CCUG 29542T

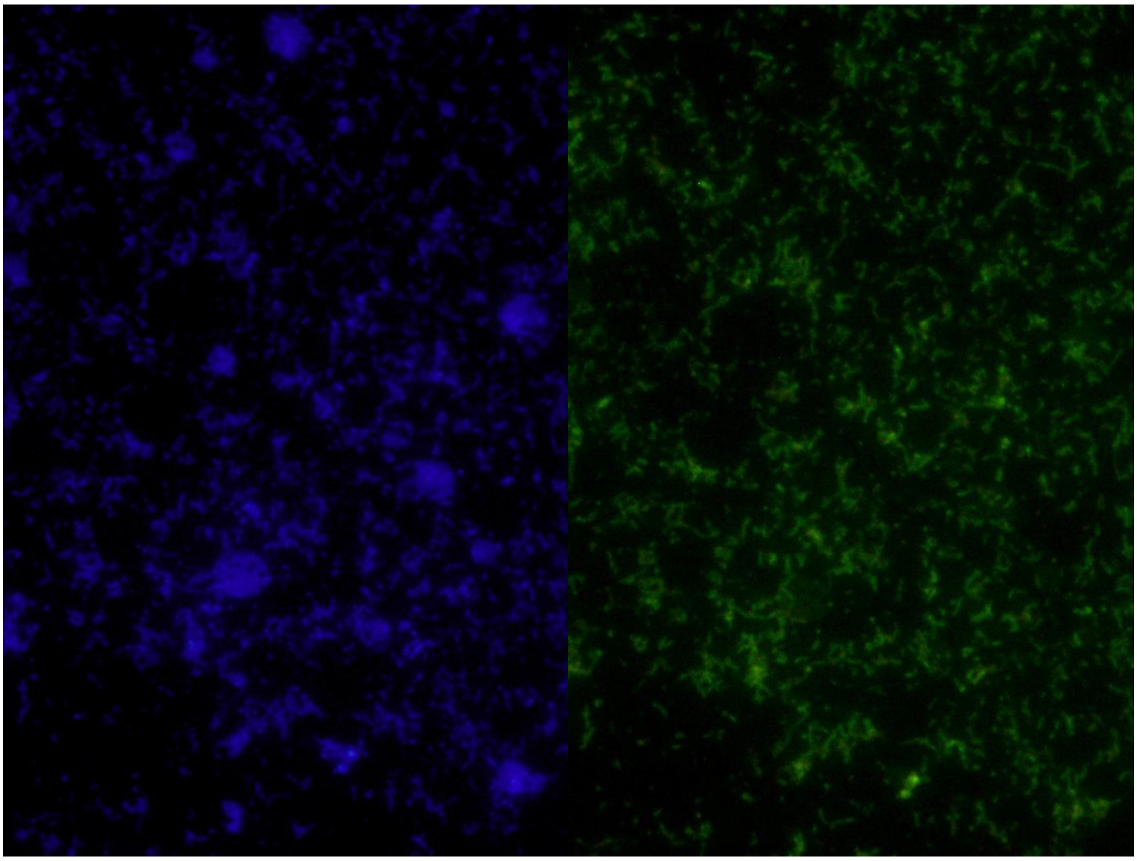

Prevotella disiens CCUG 59491

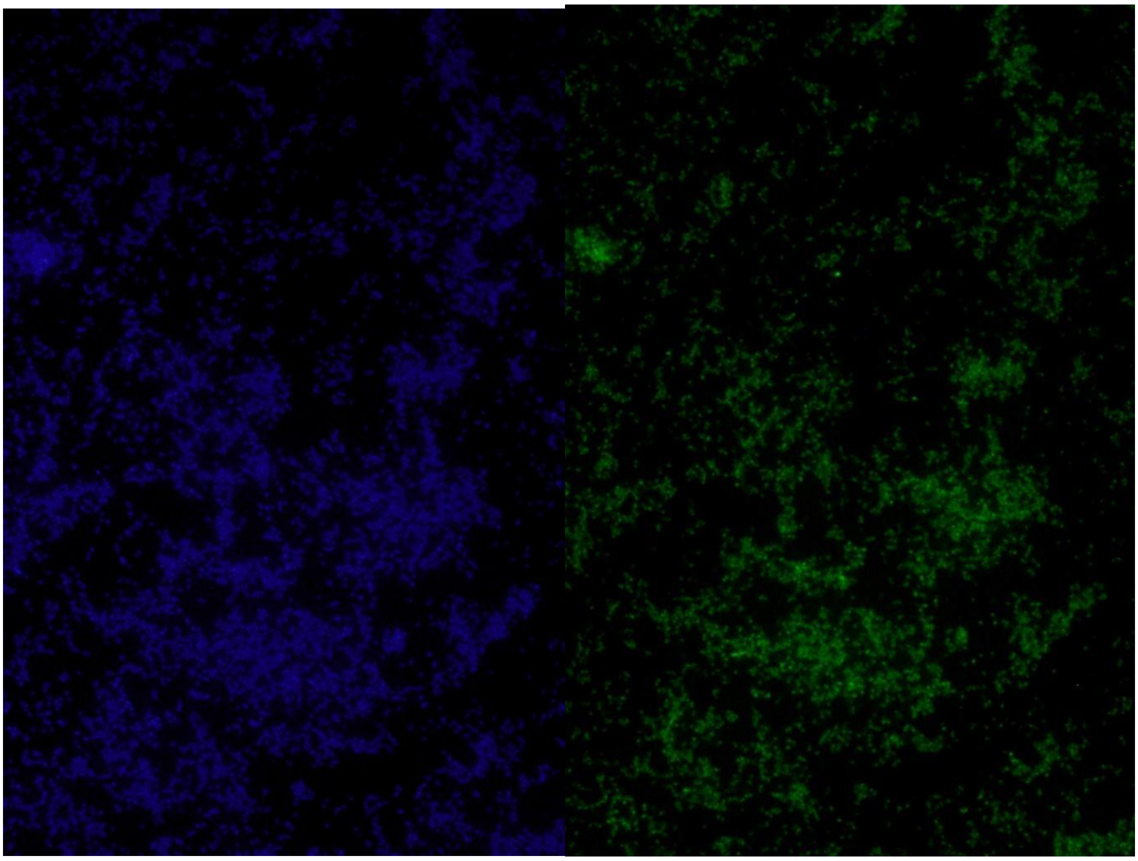

Prevotella fusca CCUG 57946

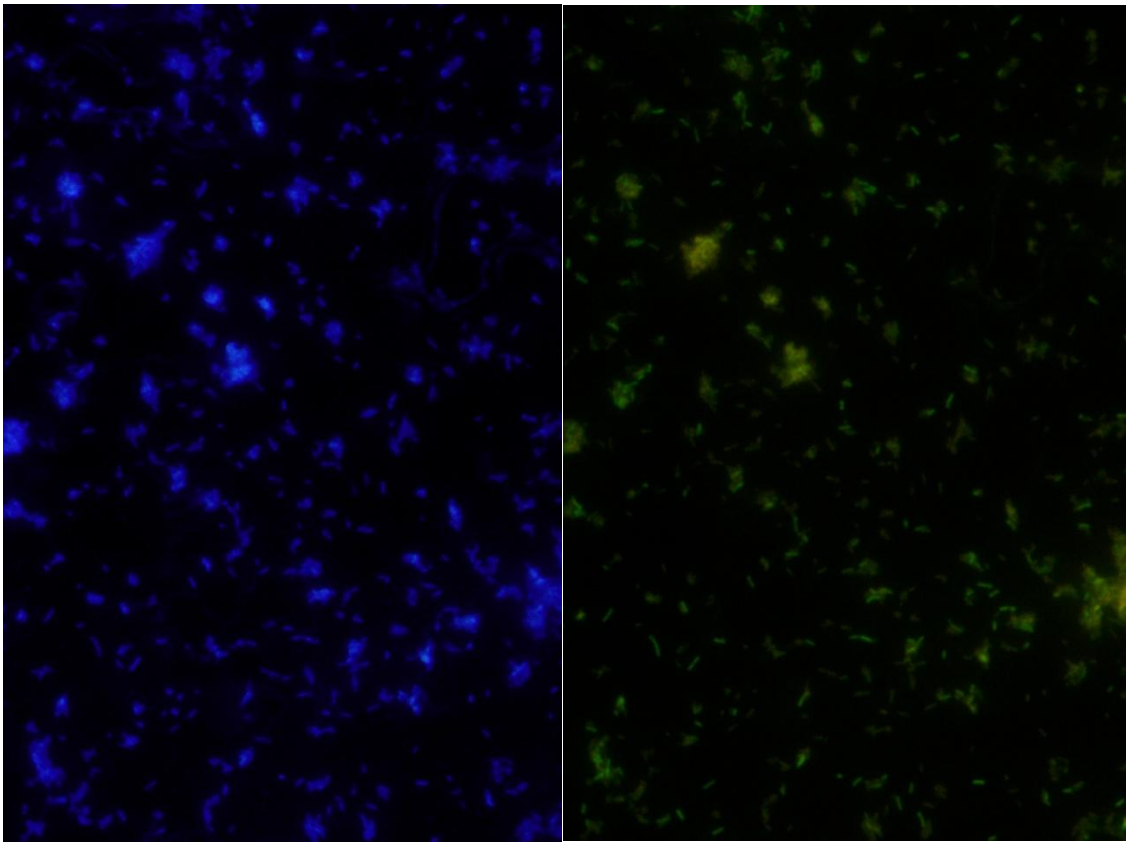

Prevotella histicola CCUG 55407

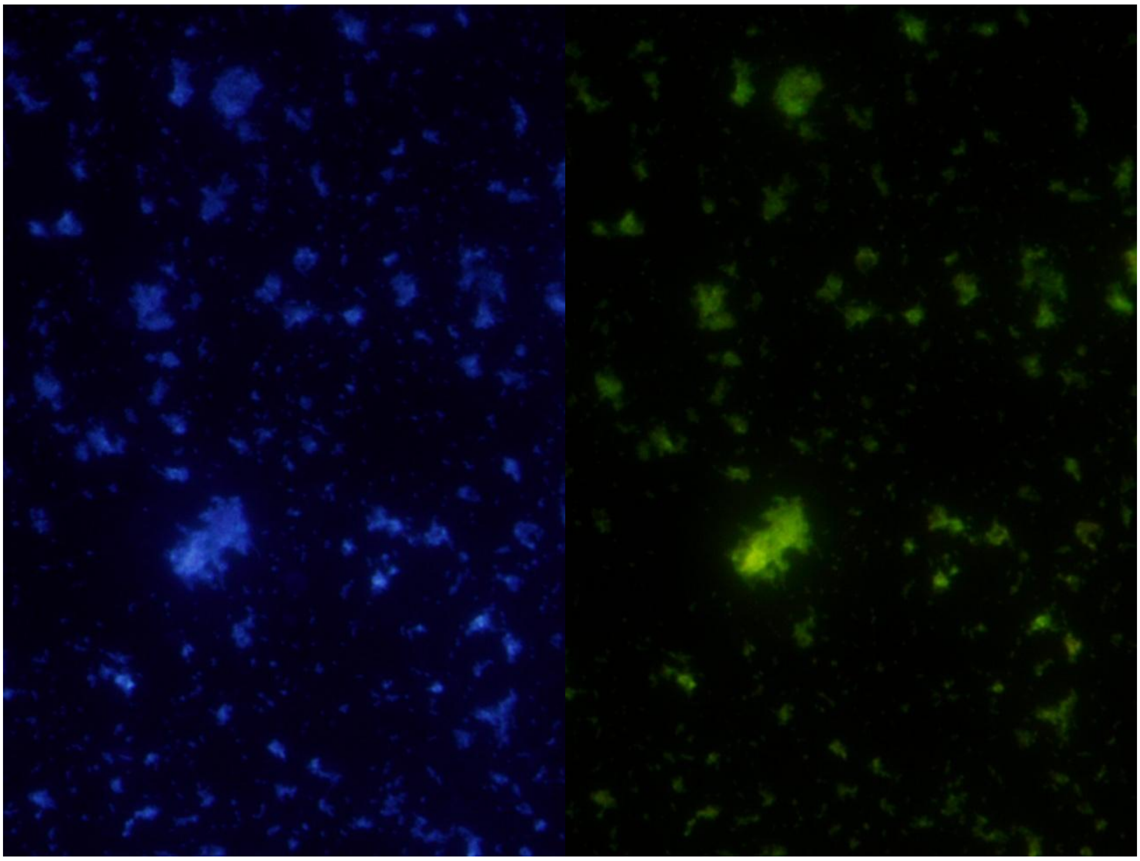

Prevotella illustrans CCUG 72806

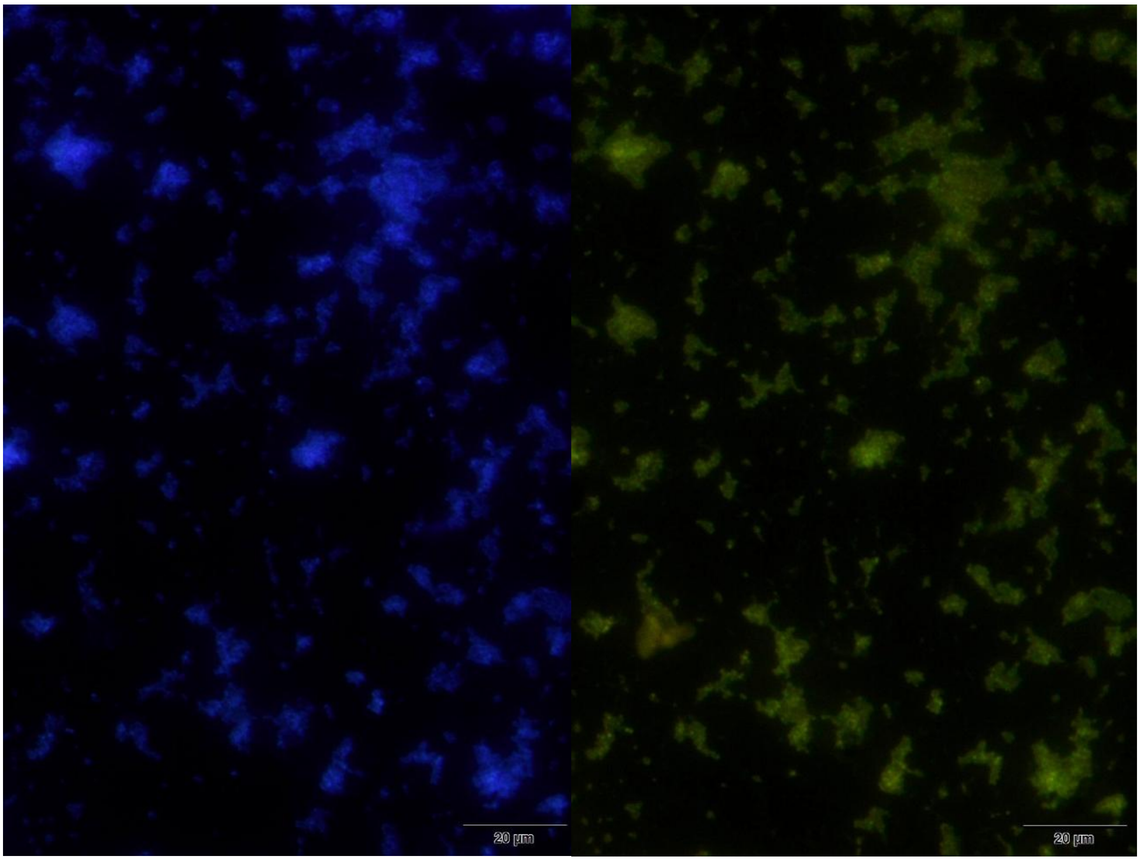

Prevotella imum CCUG 65911

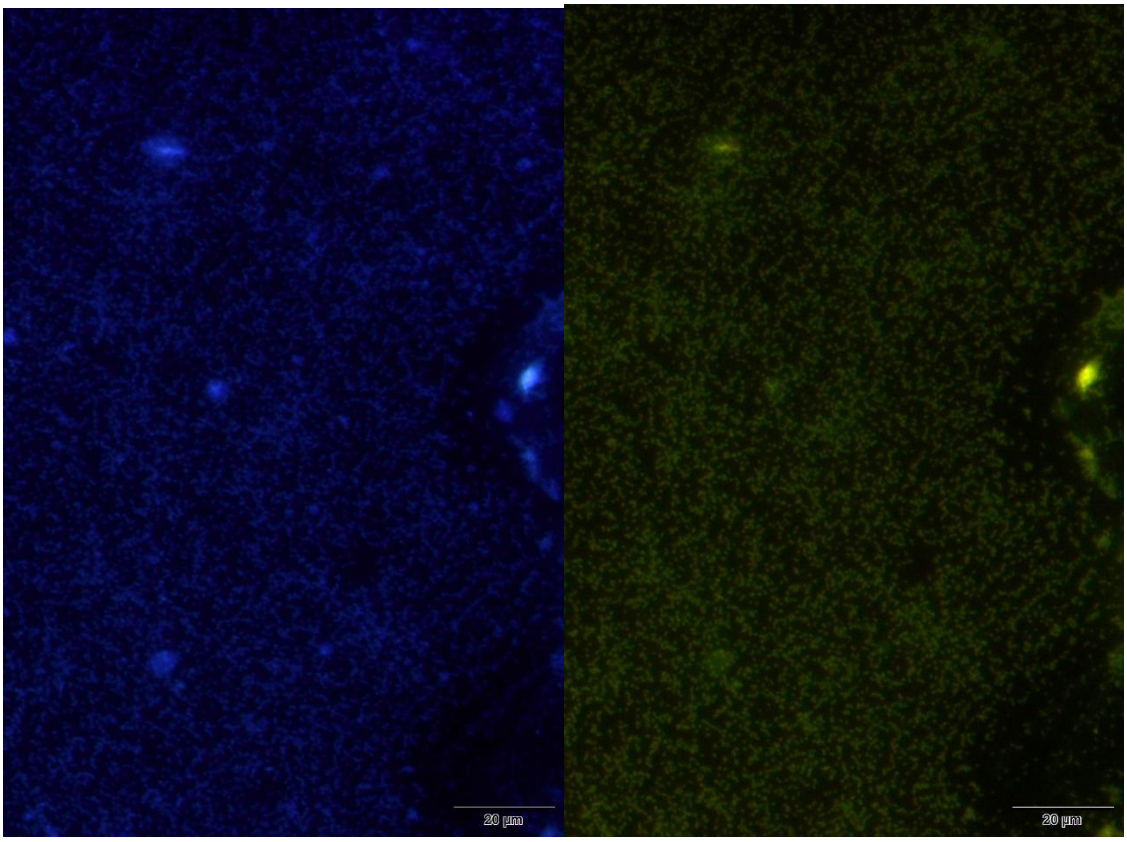

Prevotella intermedia CCUG 31410

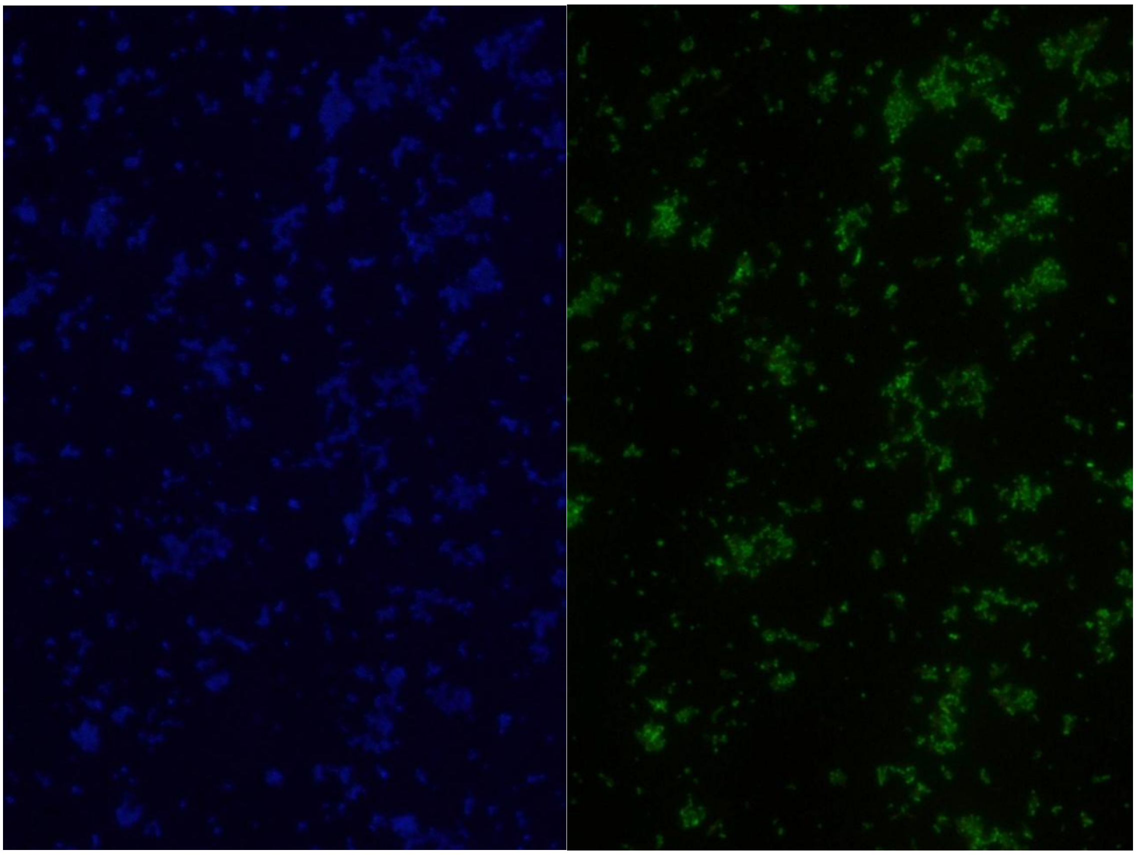

Prevotella jejuni CCUG 60371

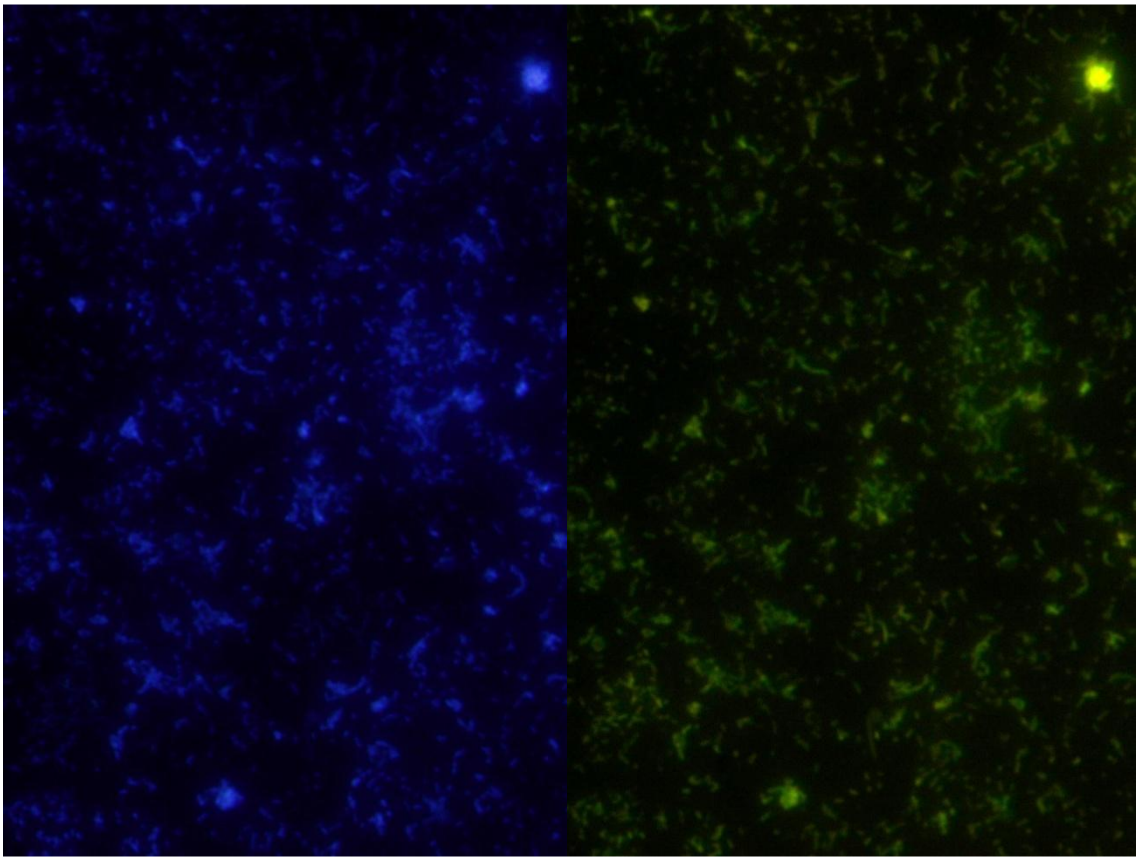

Prevotella melaninogenica CCUG 65141

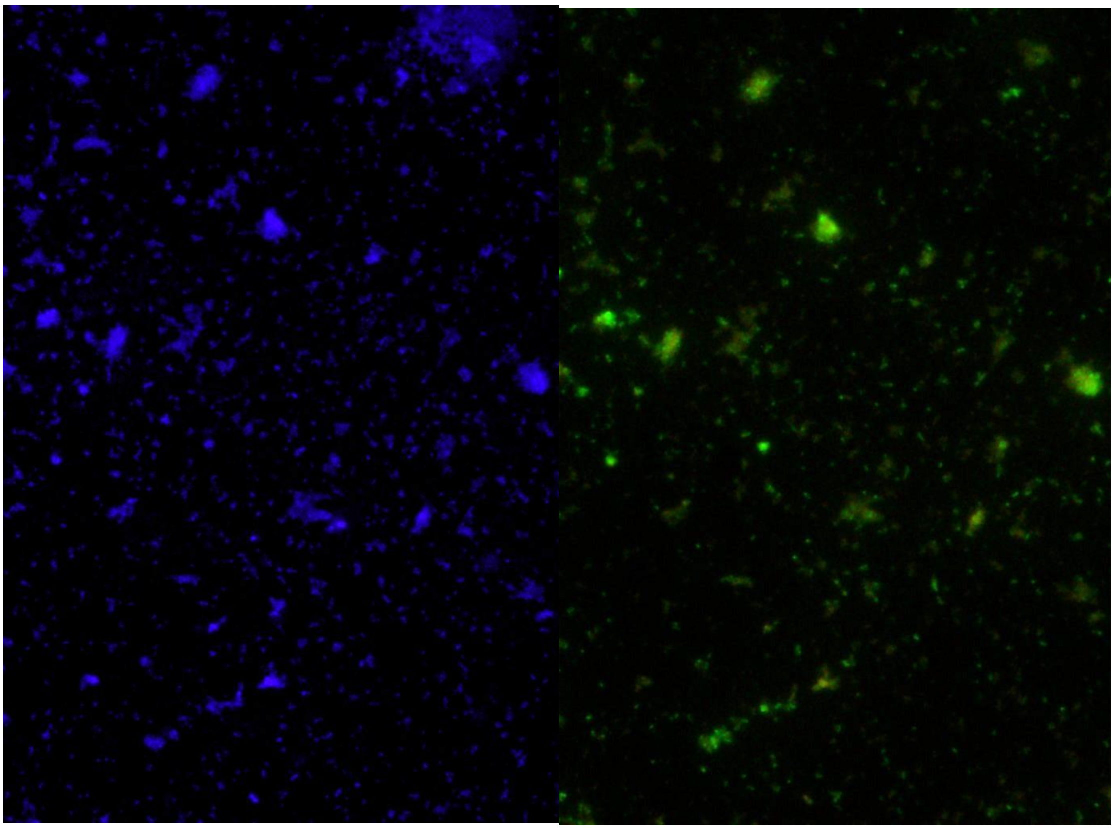

Prevotella micans CCUG 56105

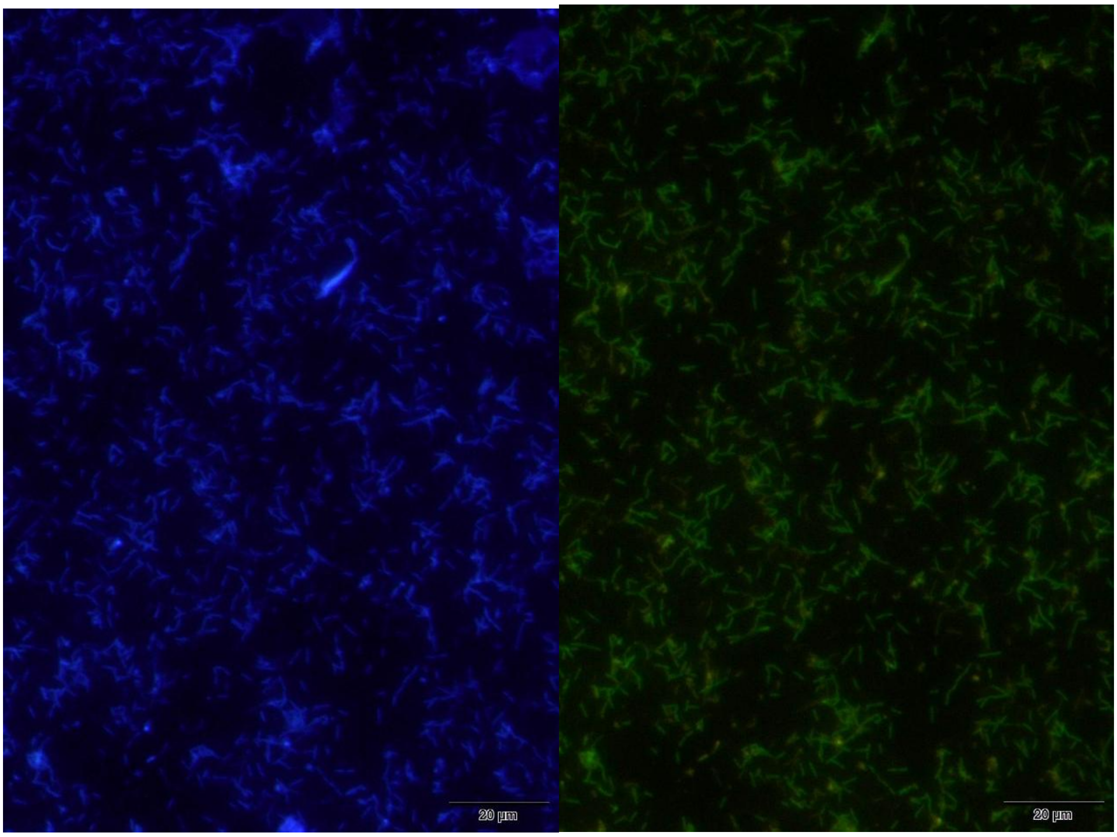

Prevotella multiformis CCUG 51937

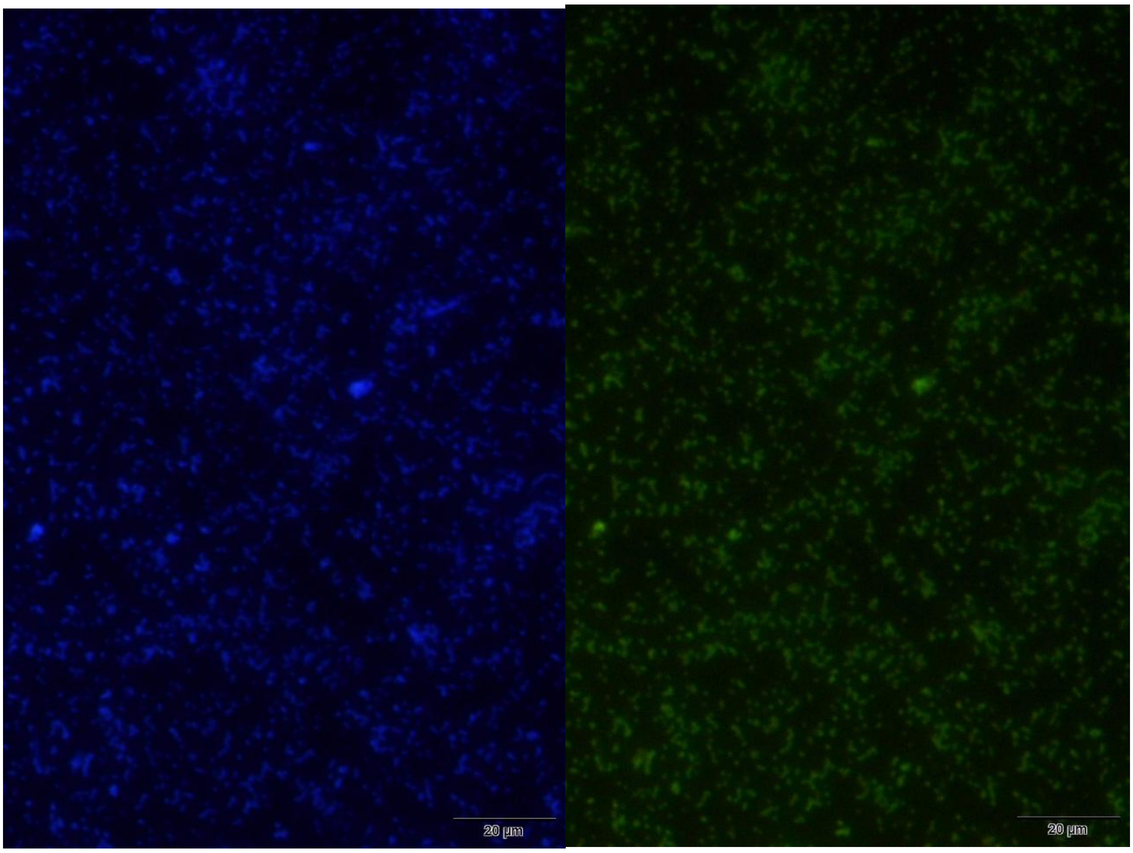

Prevotella nigrescens CCUG 25289

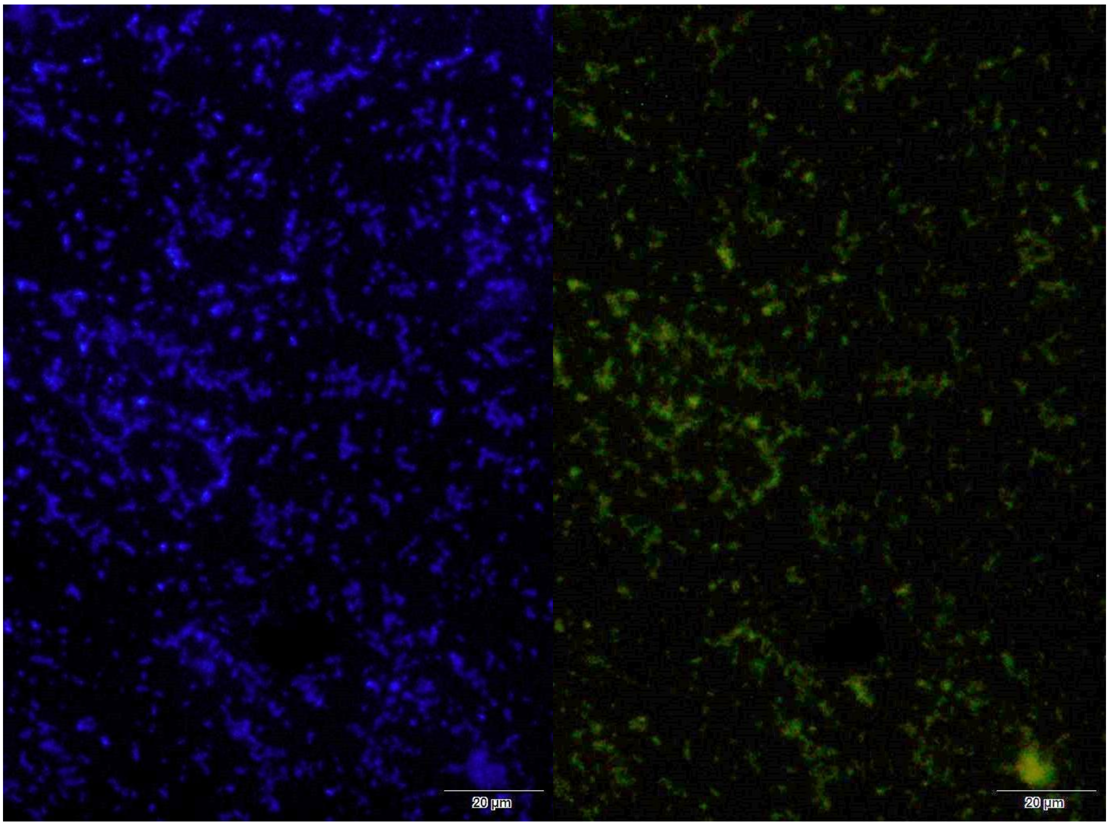

Prevotella pallens CCUG 39484

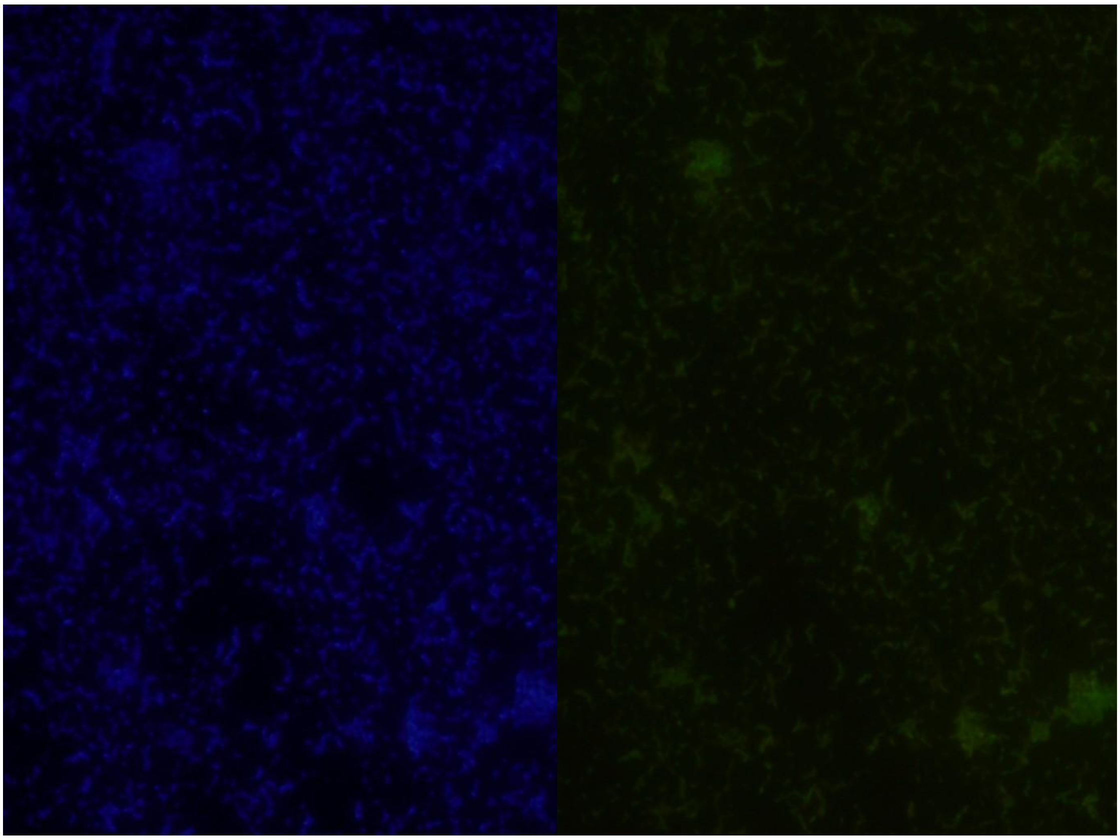

Prevotella scopos CCUG 57945

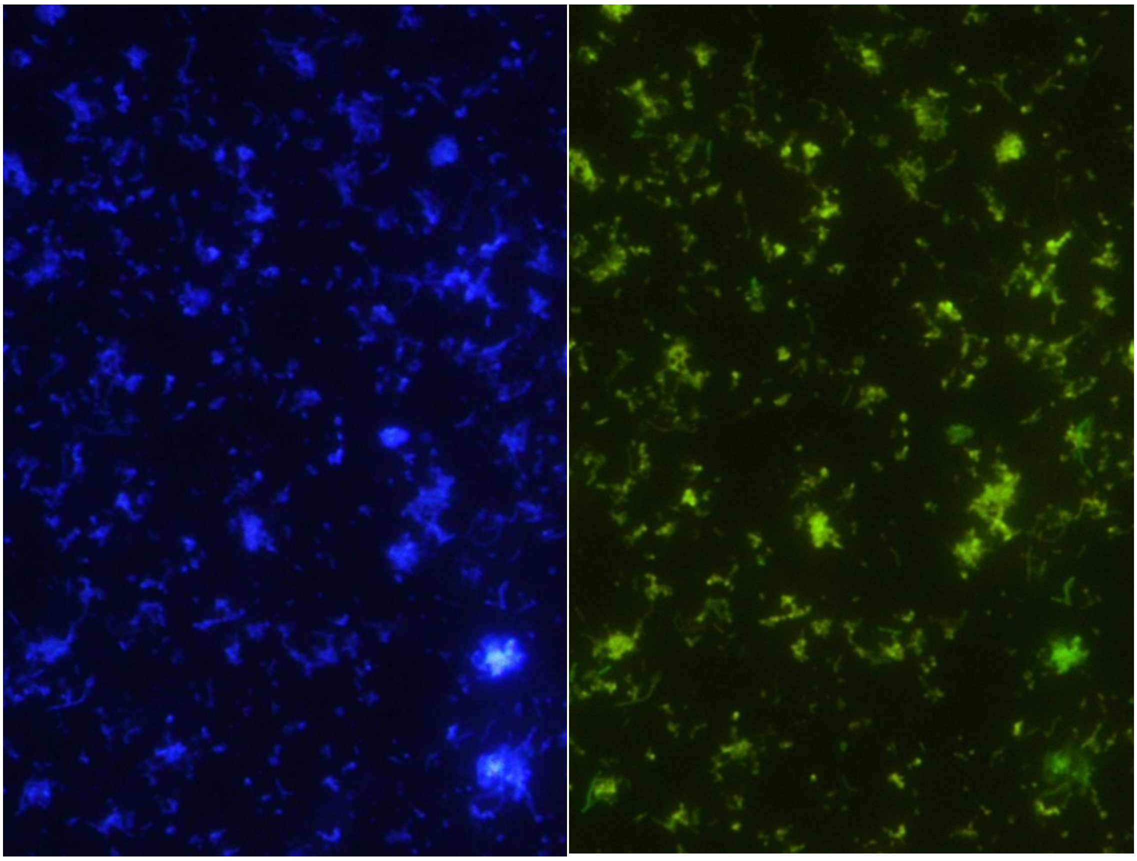

Prevotella timonensis CCUG 59487

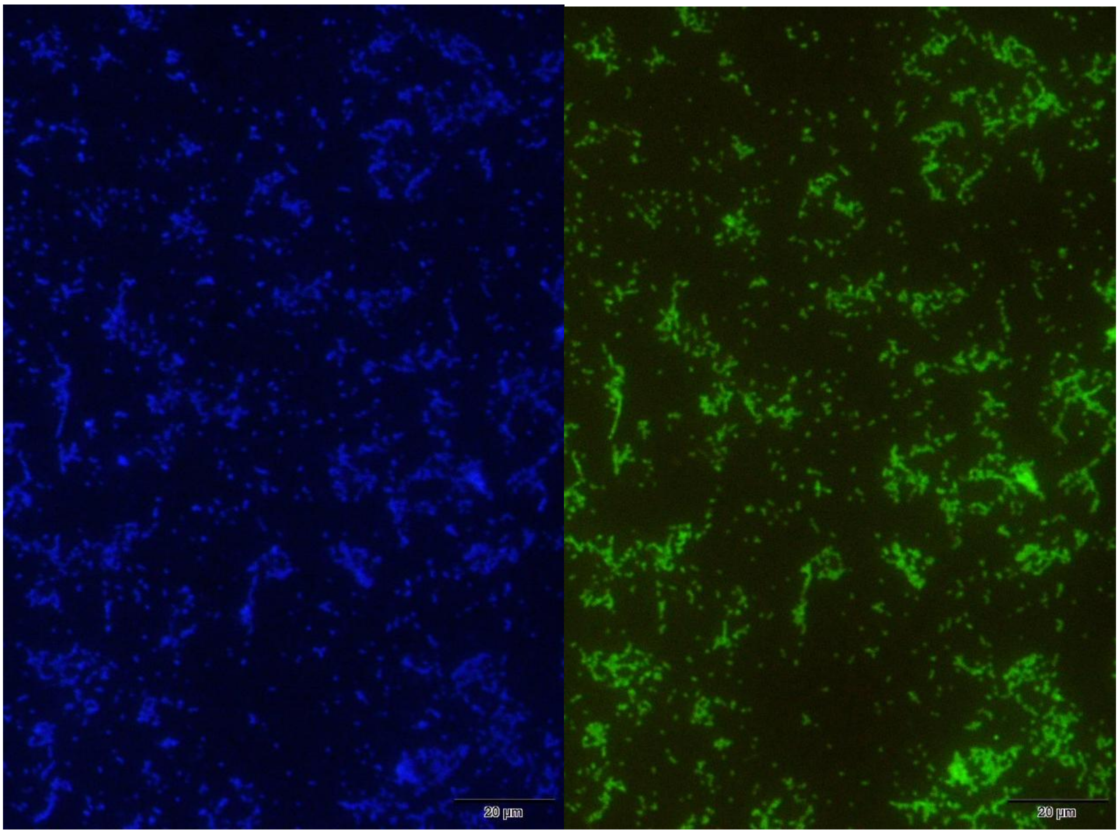

Prevotella veroralis CCUG 15422

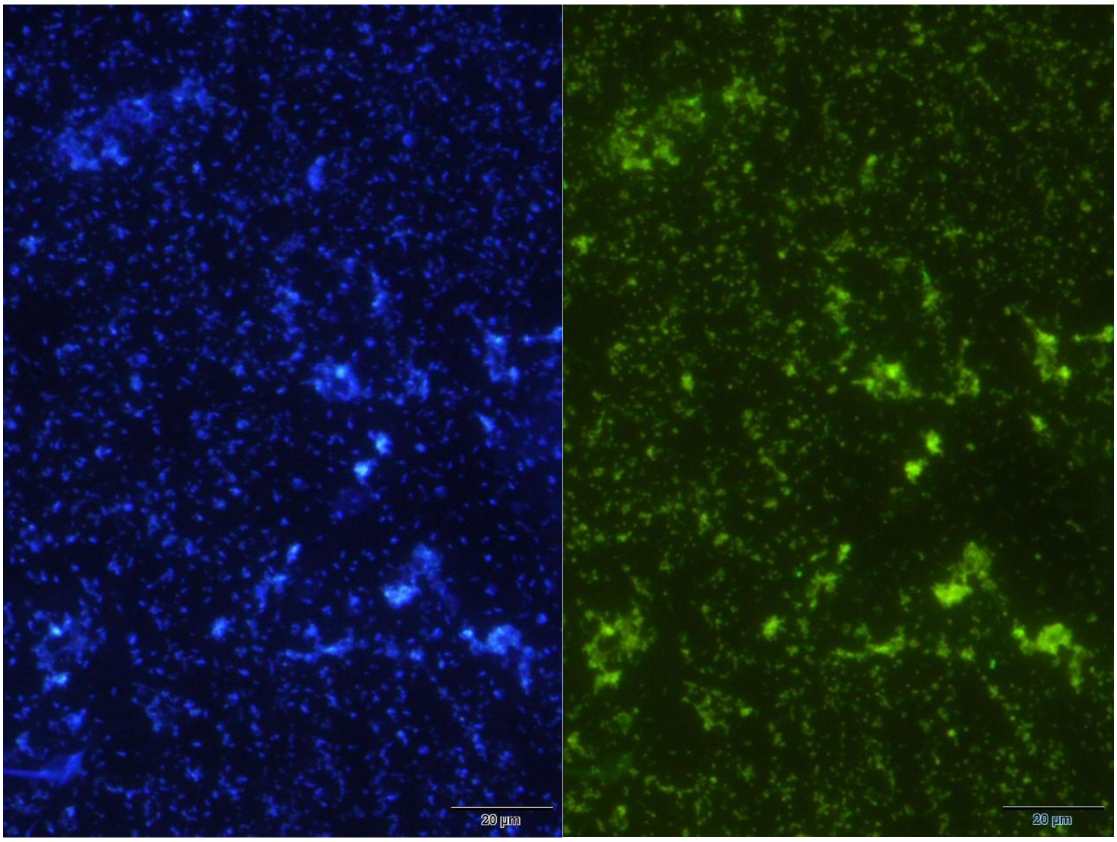

Prevotella vespertine CCUG 72808

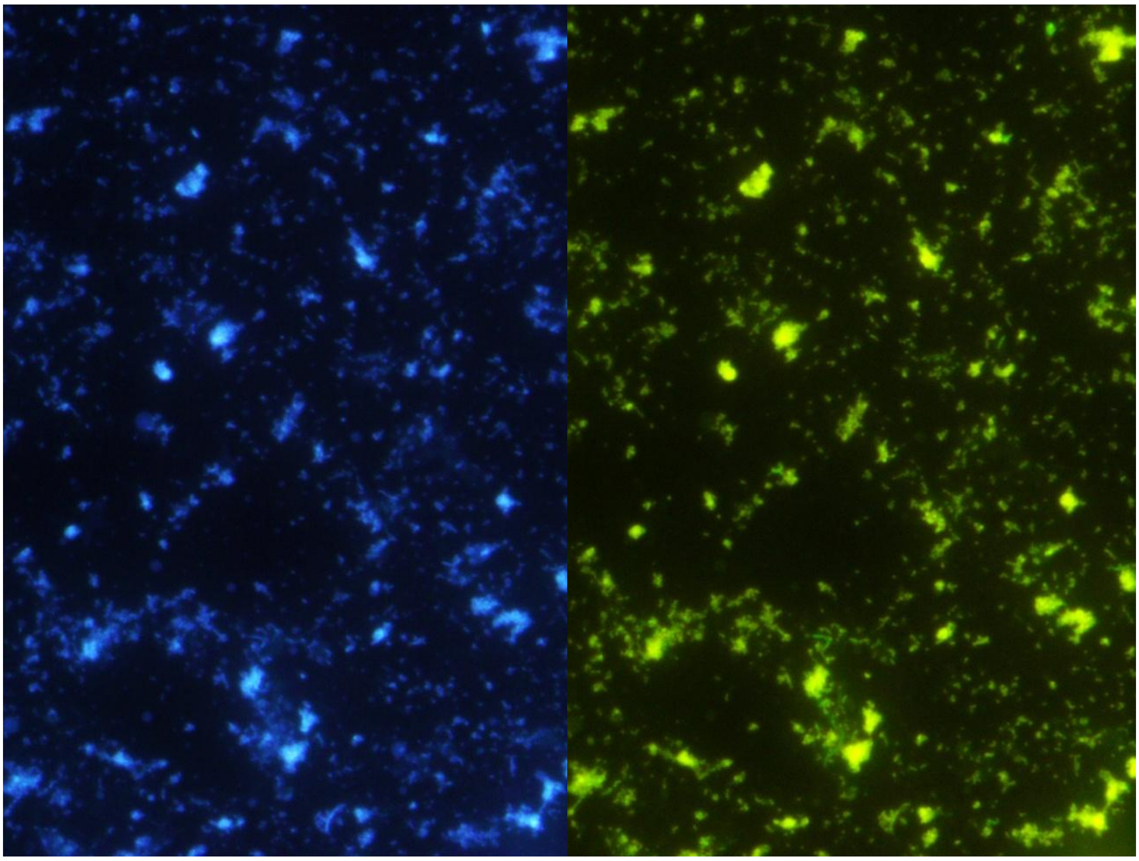

Propionibacterium acnes UM034

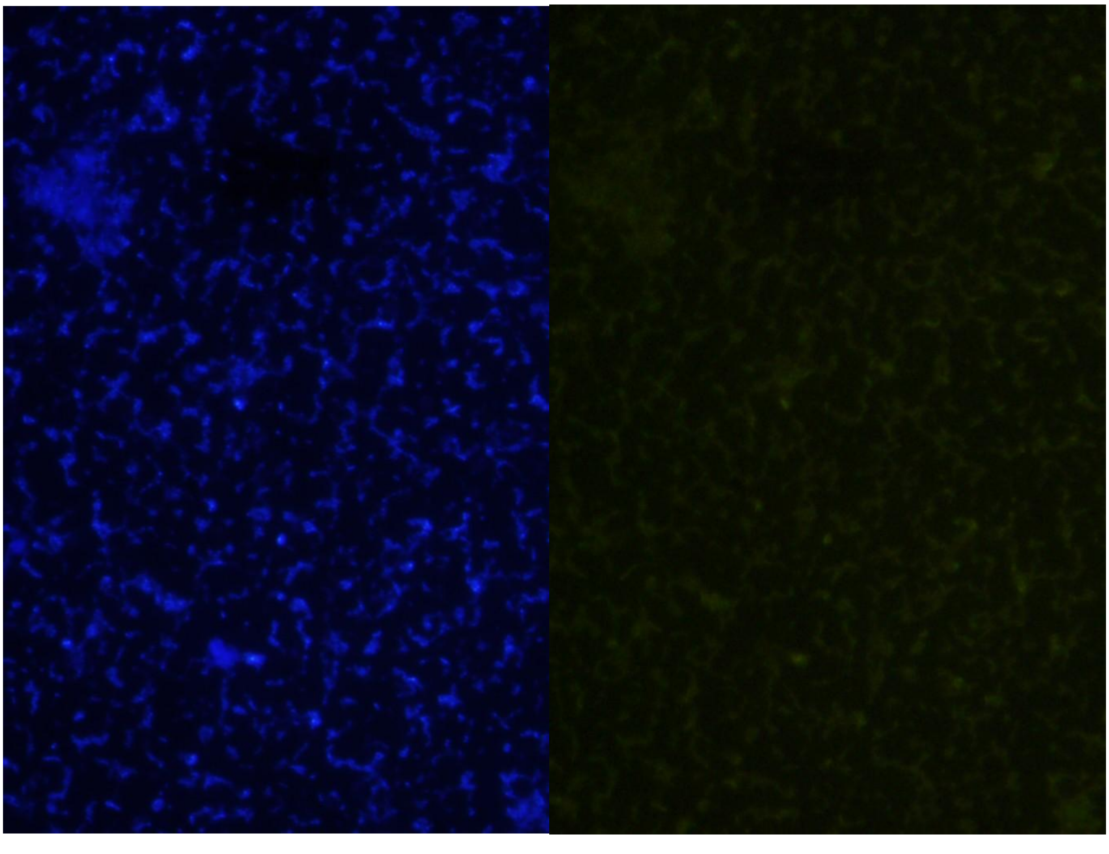

Shigella spp. UM137

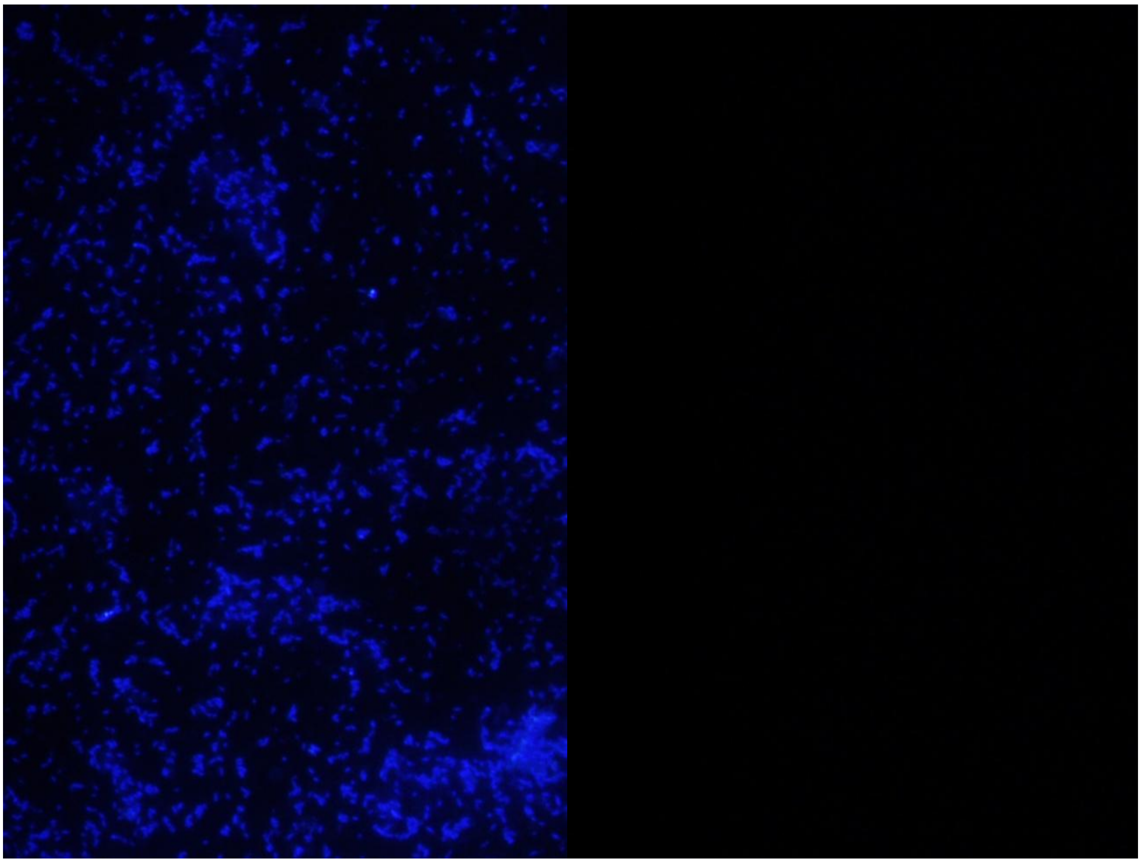

Sneathia sanguinegens CCUG 66076

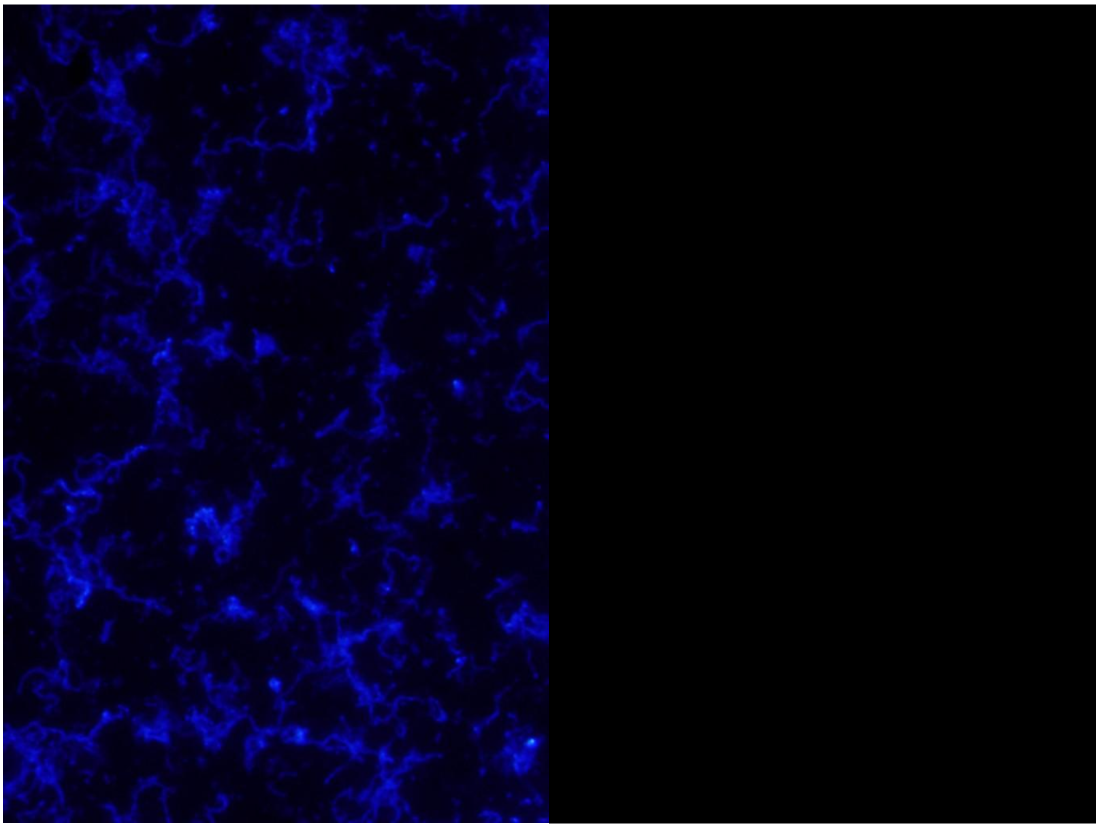

Staphylococcus epidermidis UM066

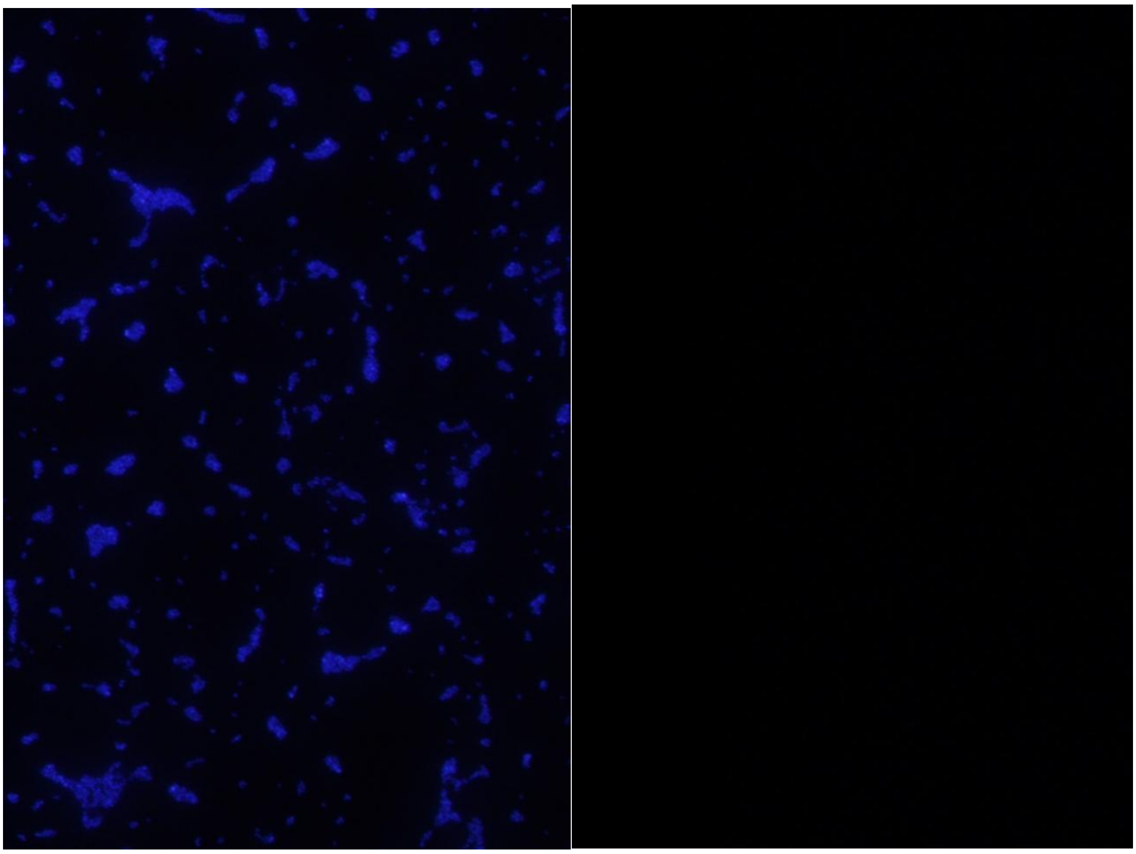

Staphylococcus haemolyticus UM066

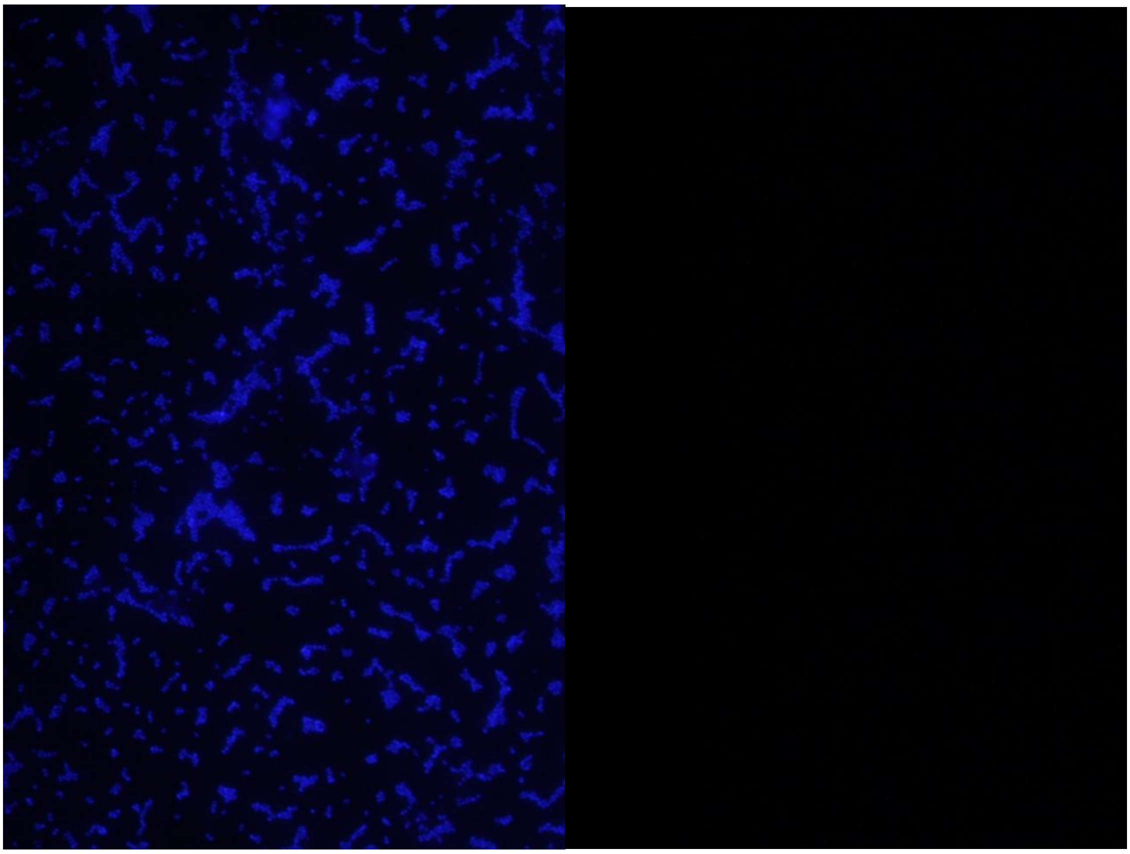

Staphylococcus hominis UM224

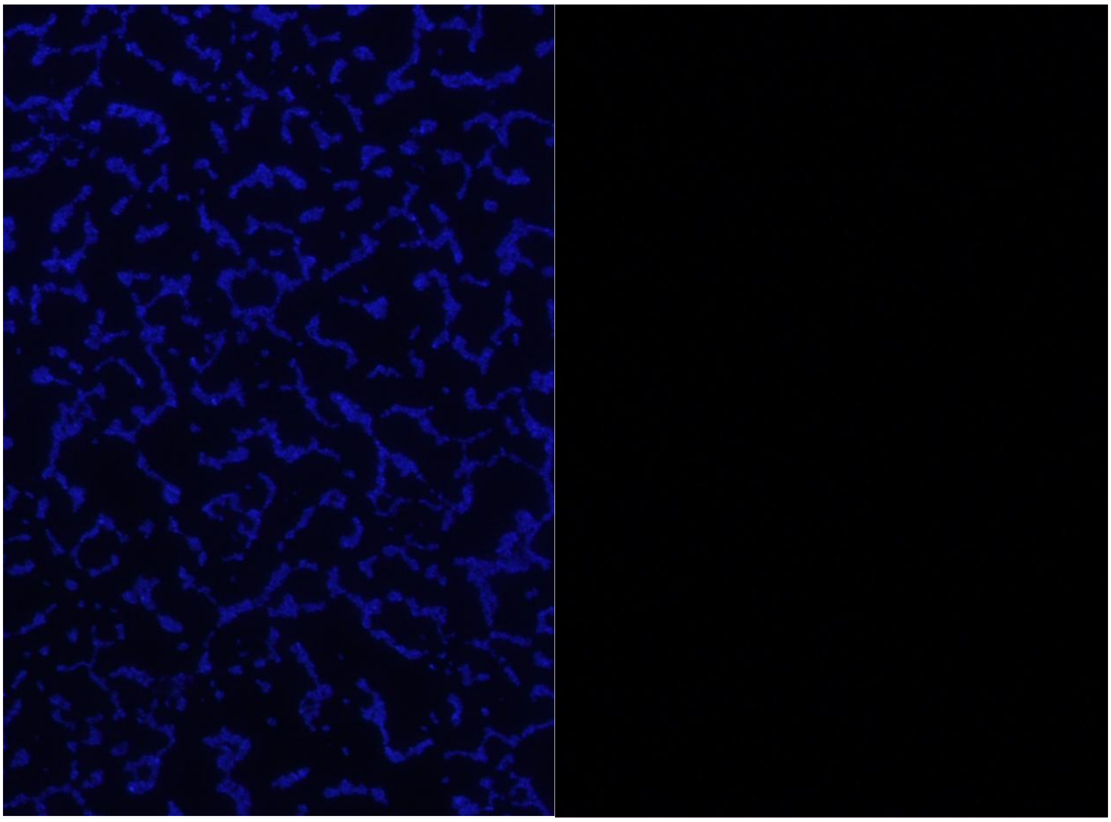

Staphylococcus saprophyticus UM121

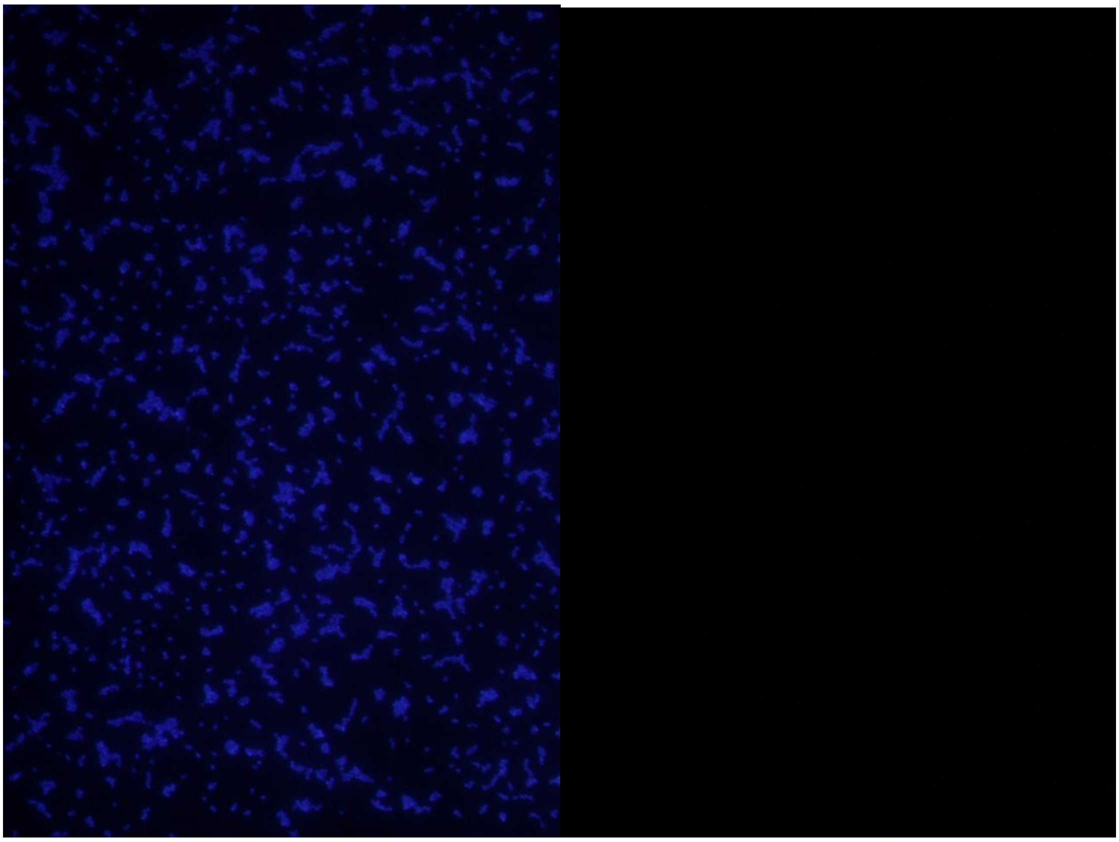

Staphylococcus simulans UM059

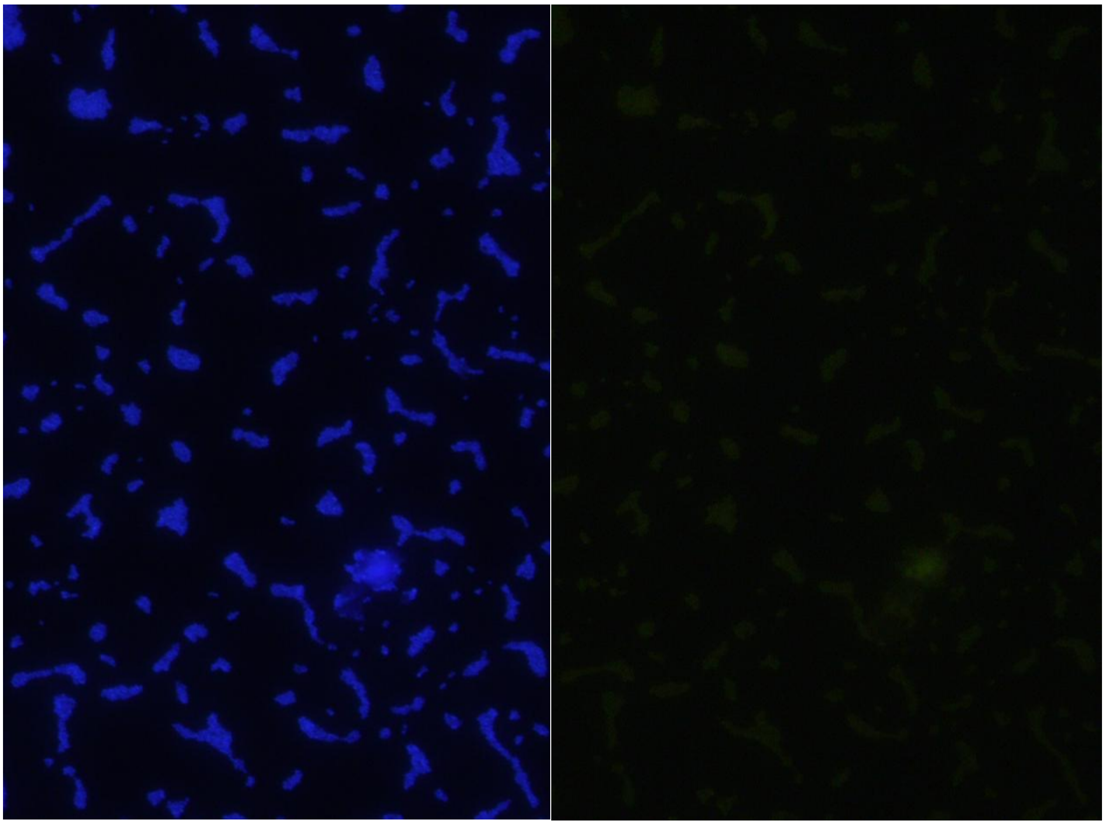

Streptococcus agalactiae UM035

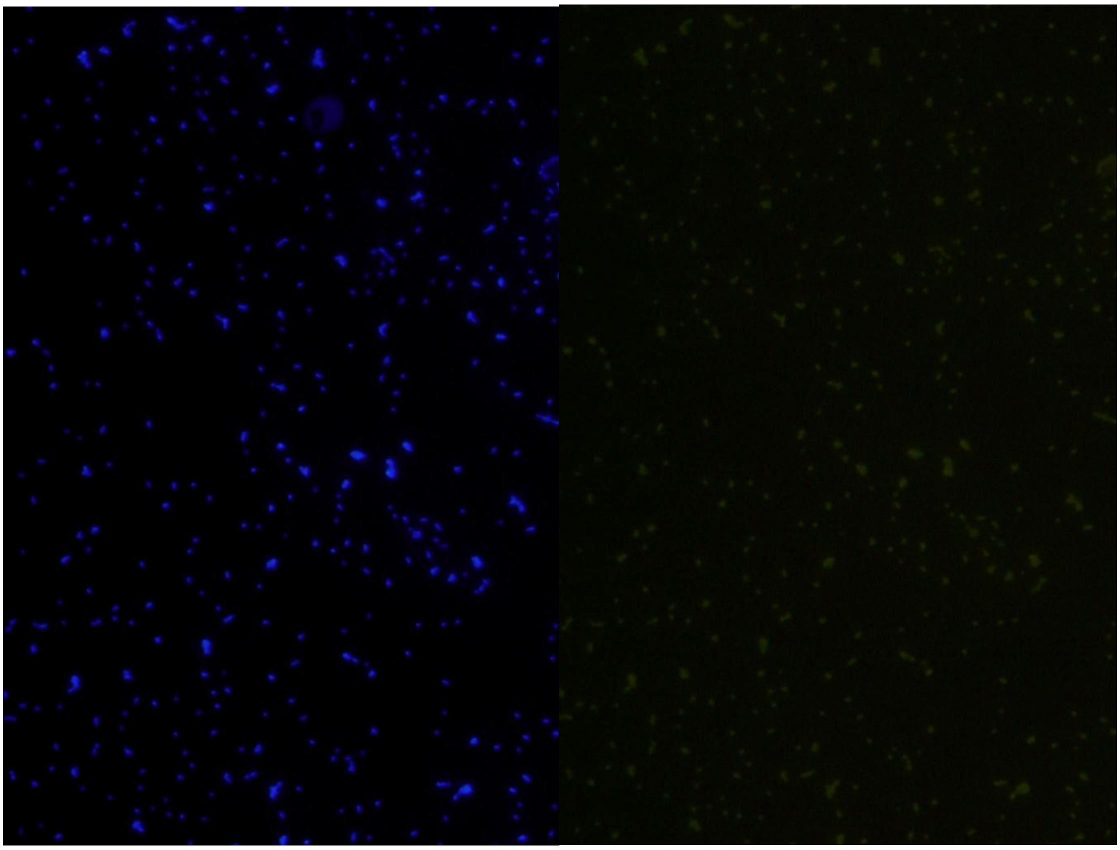

Veillonella parvula CCUG59474

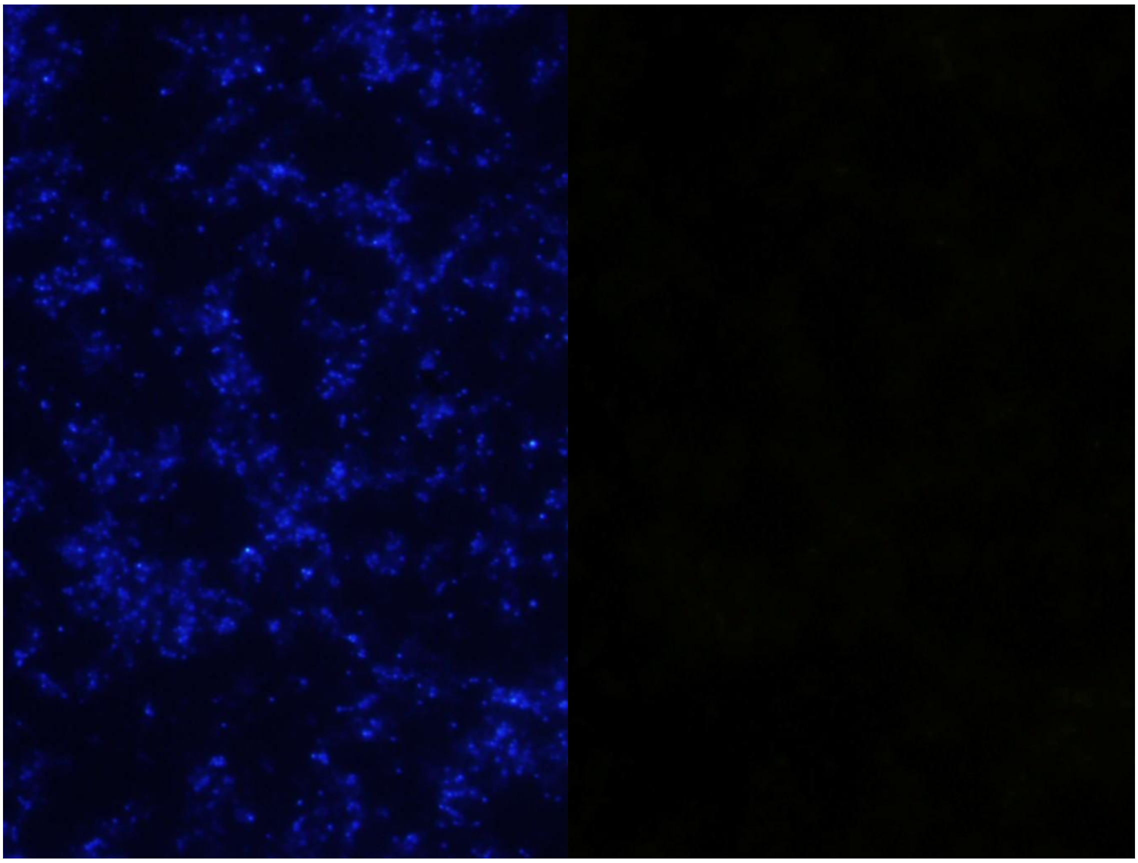

Supplementary Figure 2: The following images present the fluorescence microscopy results of *P. bivia* probe hybridization with all the tested species for sensitivity.The images were obtained using DAPI filter and FITC filter. Magnification of 400x and scale bars represent 20 μm.

Prevotella amnii CCUG 53648

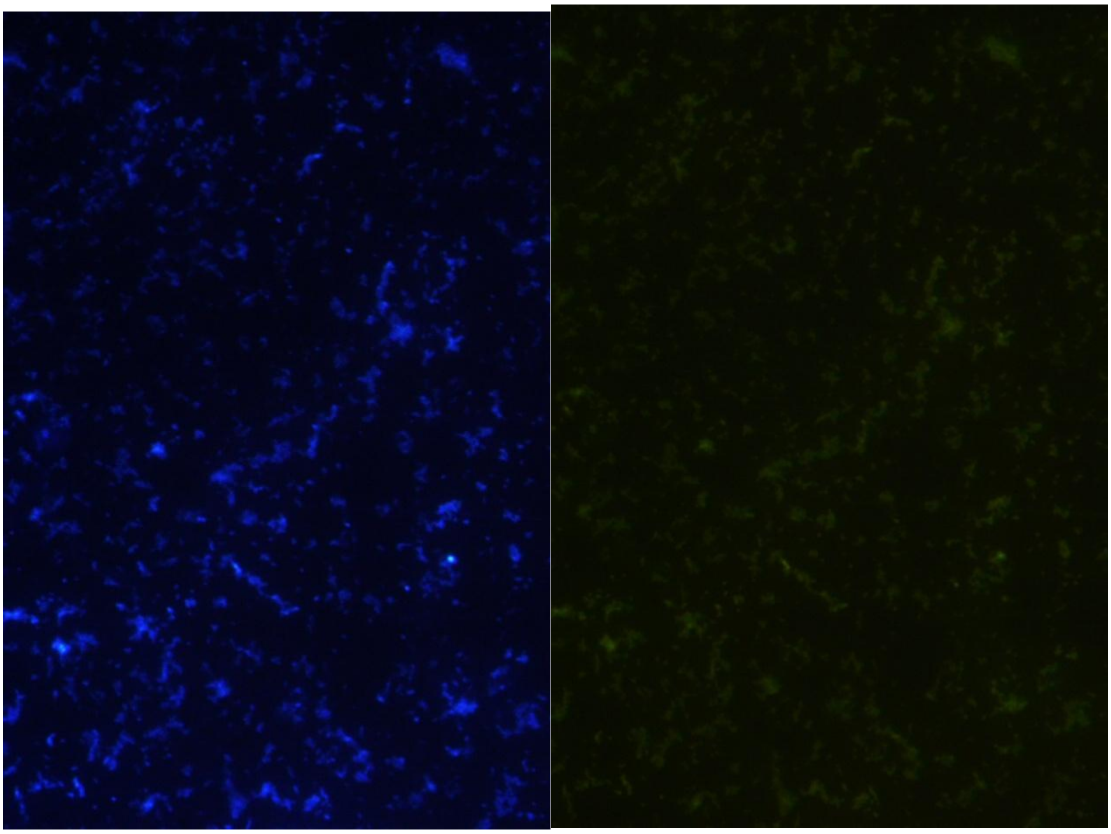

Prevotella bivia ATCC 29303

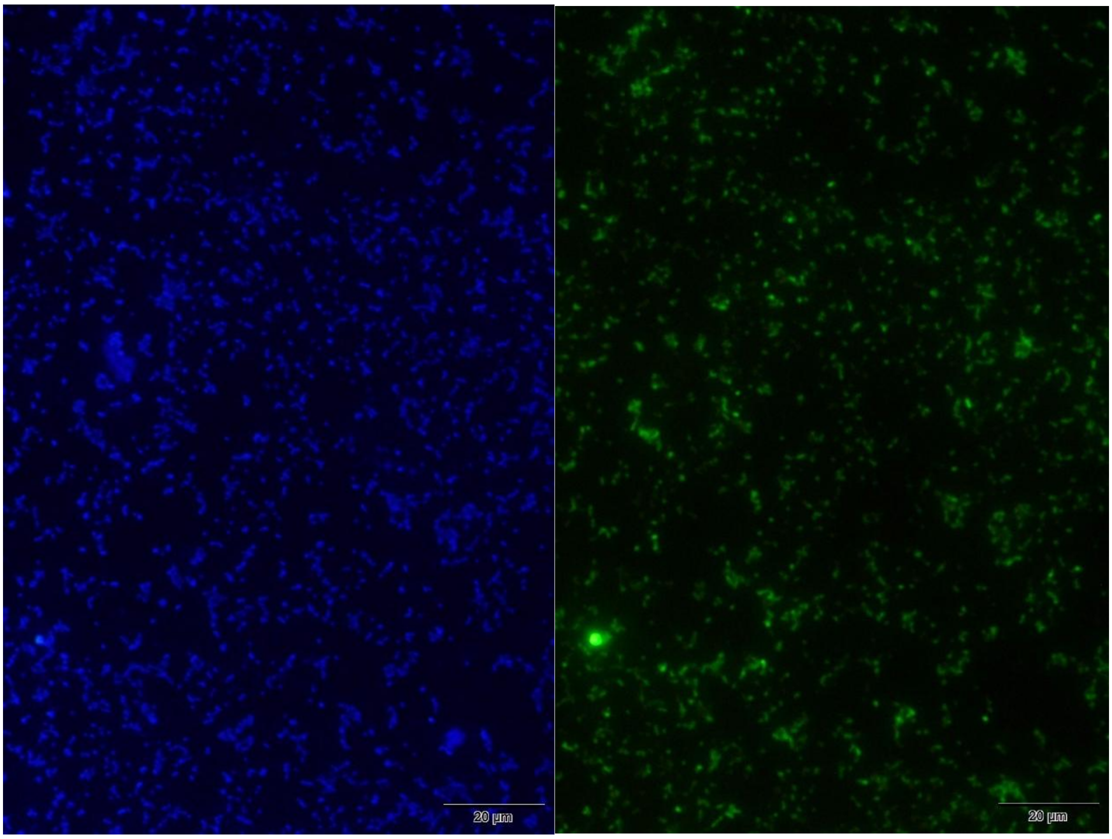

Prevotella bivia CCUG 33360

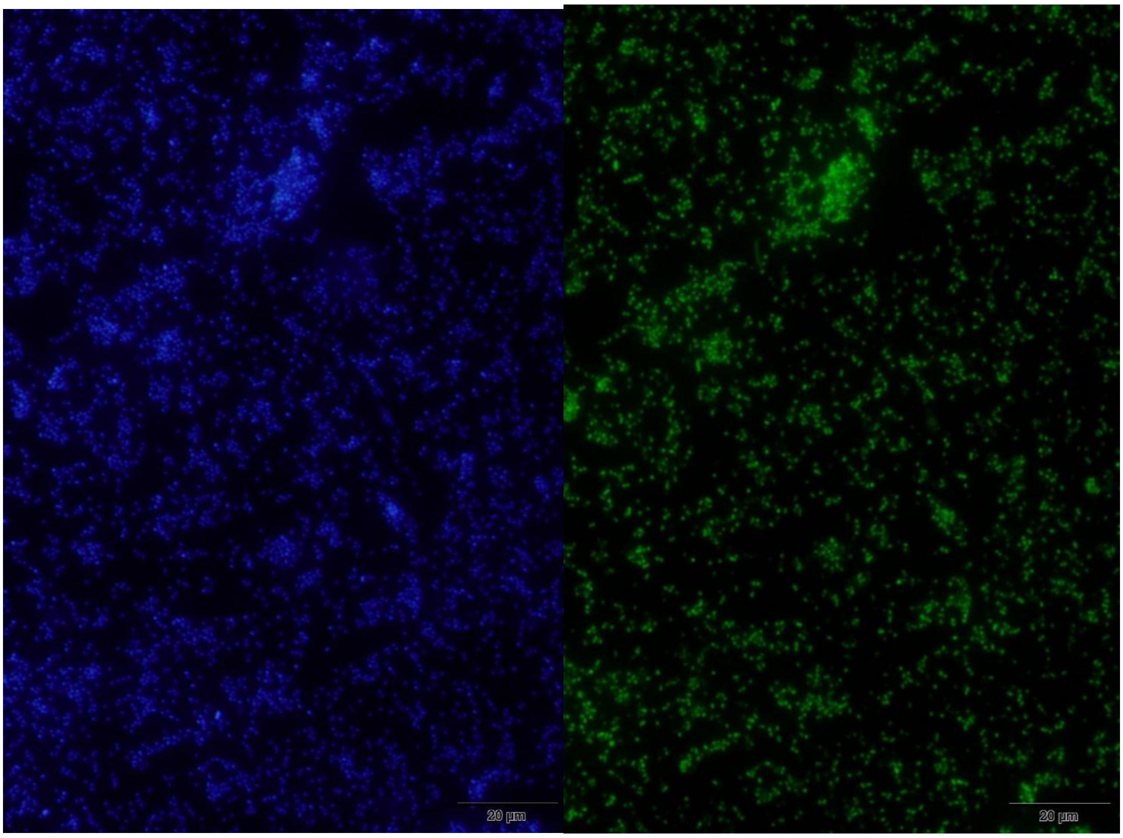

Prevotella bivia CCUG 34046

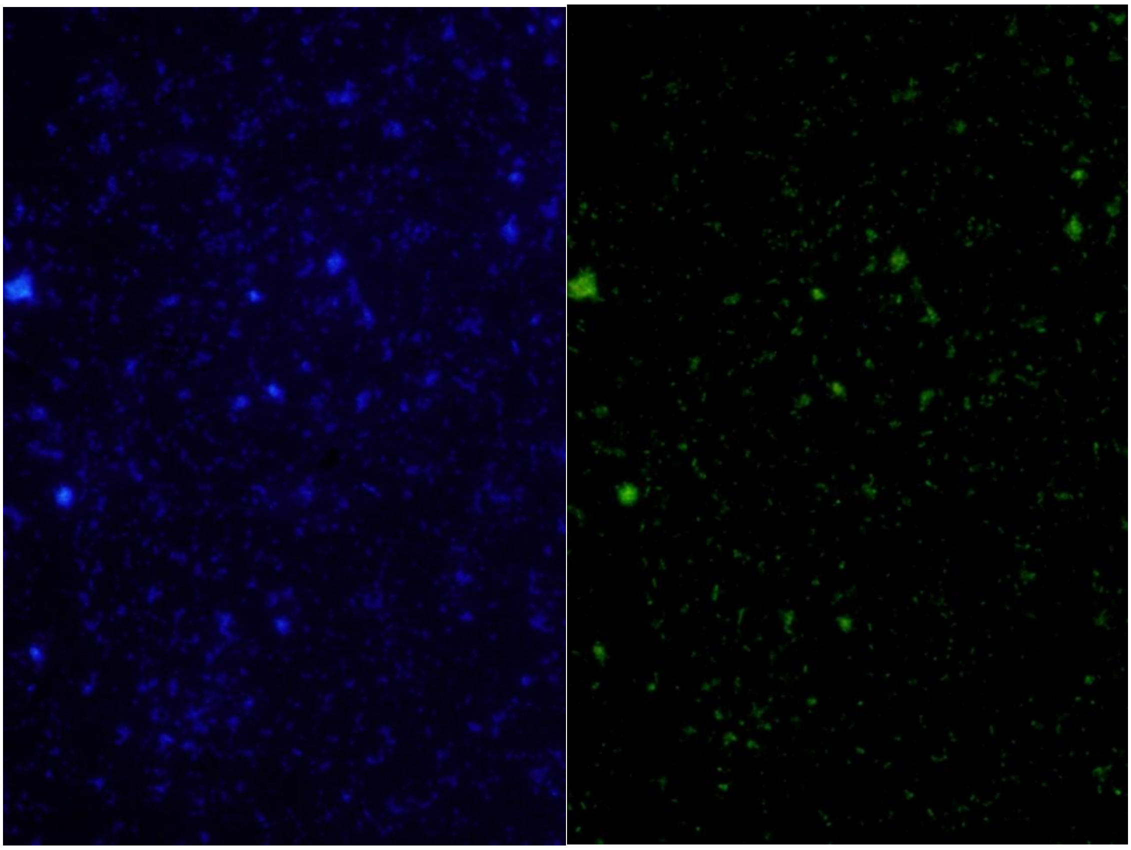

Prevotella bivia CCUG 44195

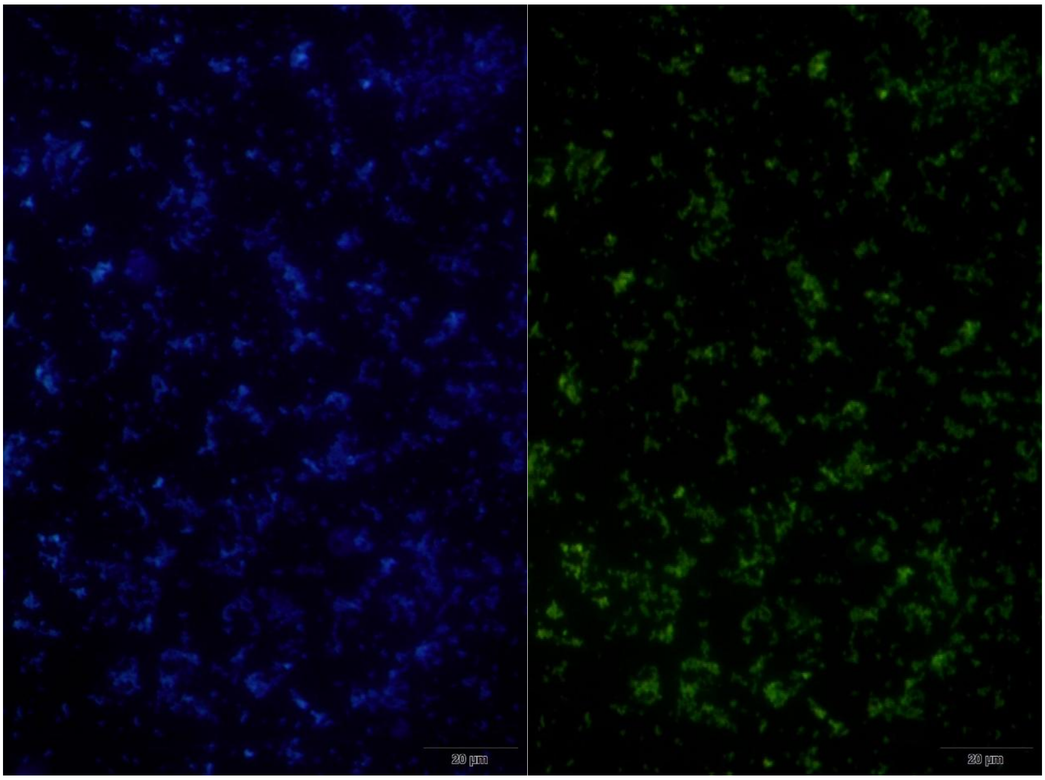

Prevotella bivia CCUG 59496

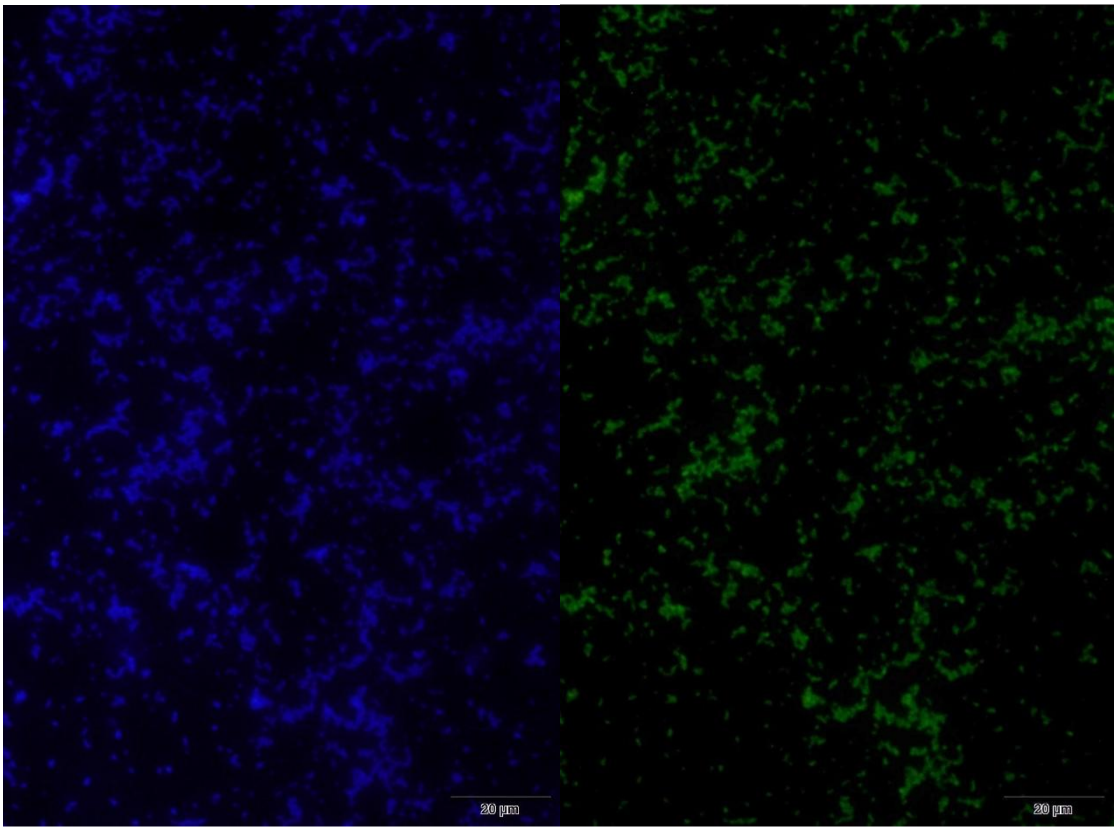

Prevotella brunnea CCUG 72809

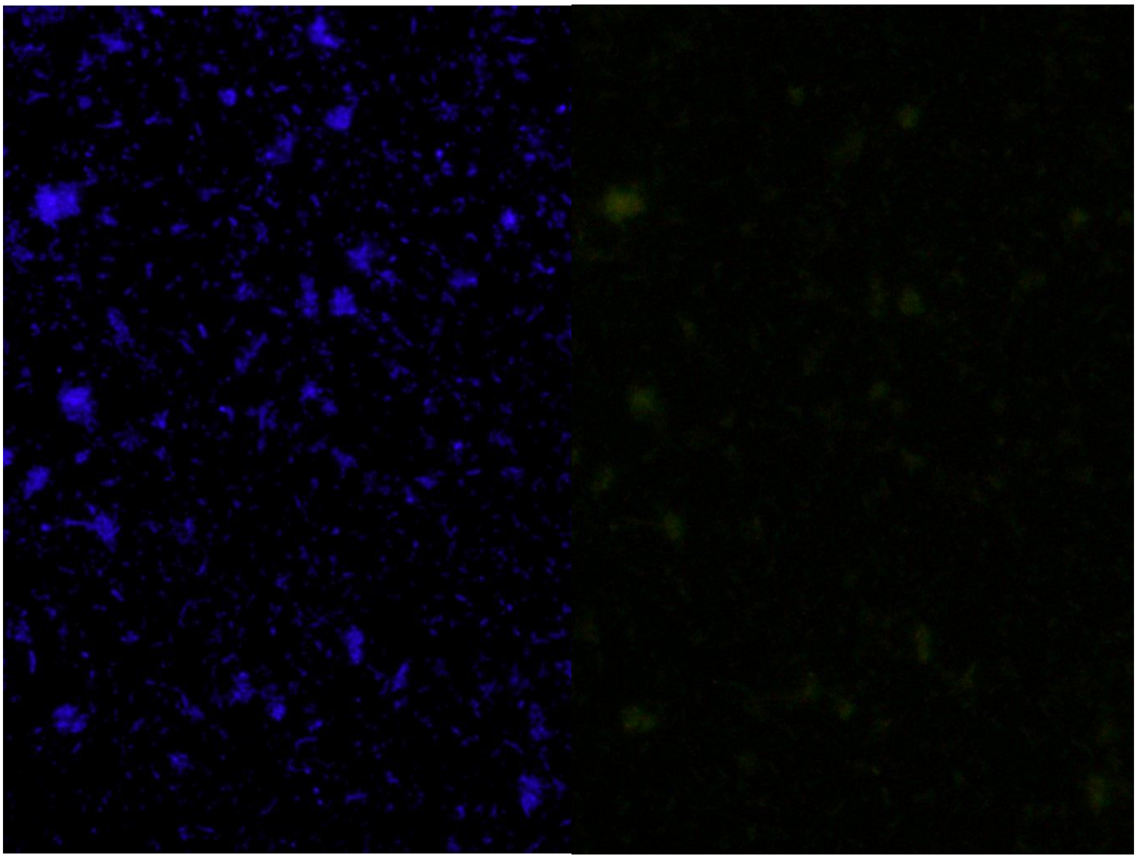

Prevotella buccalis   CCUG 44127

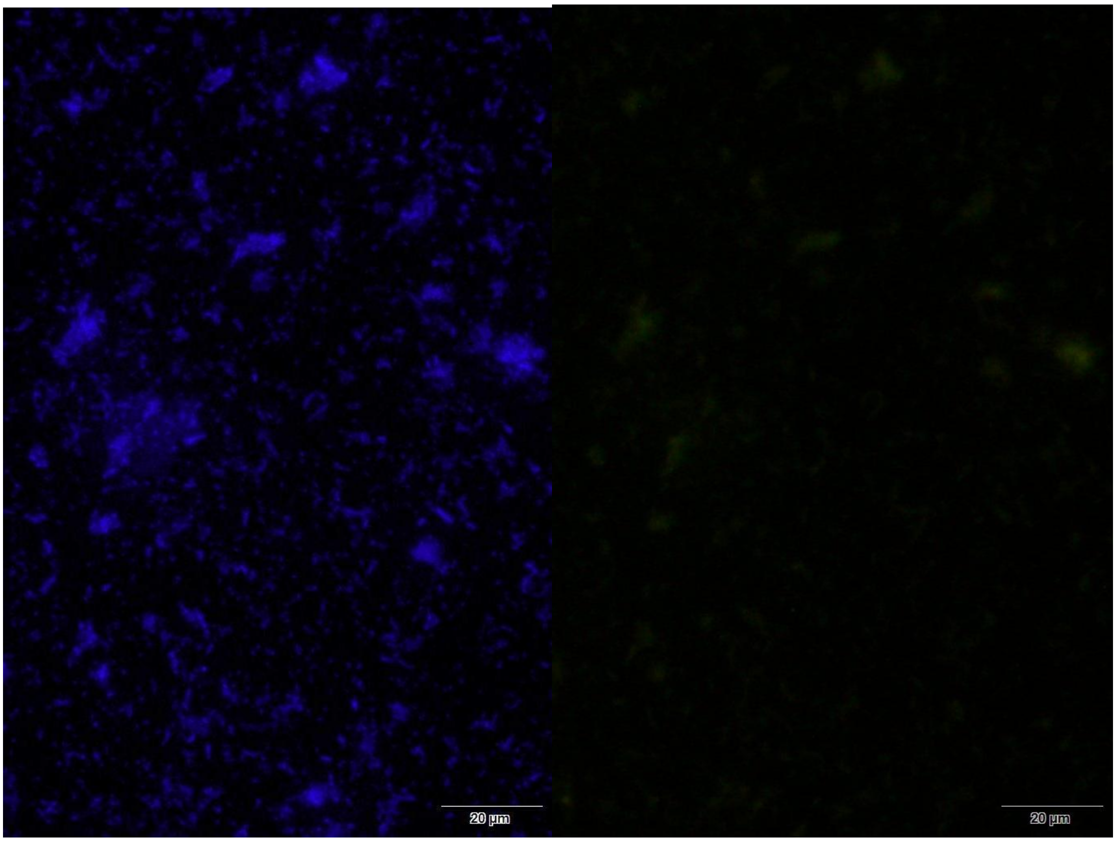

Prevotella copri   CCUG 58058T

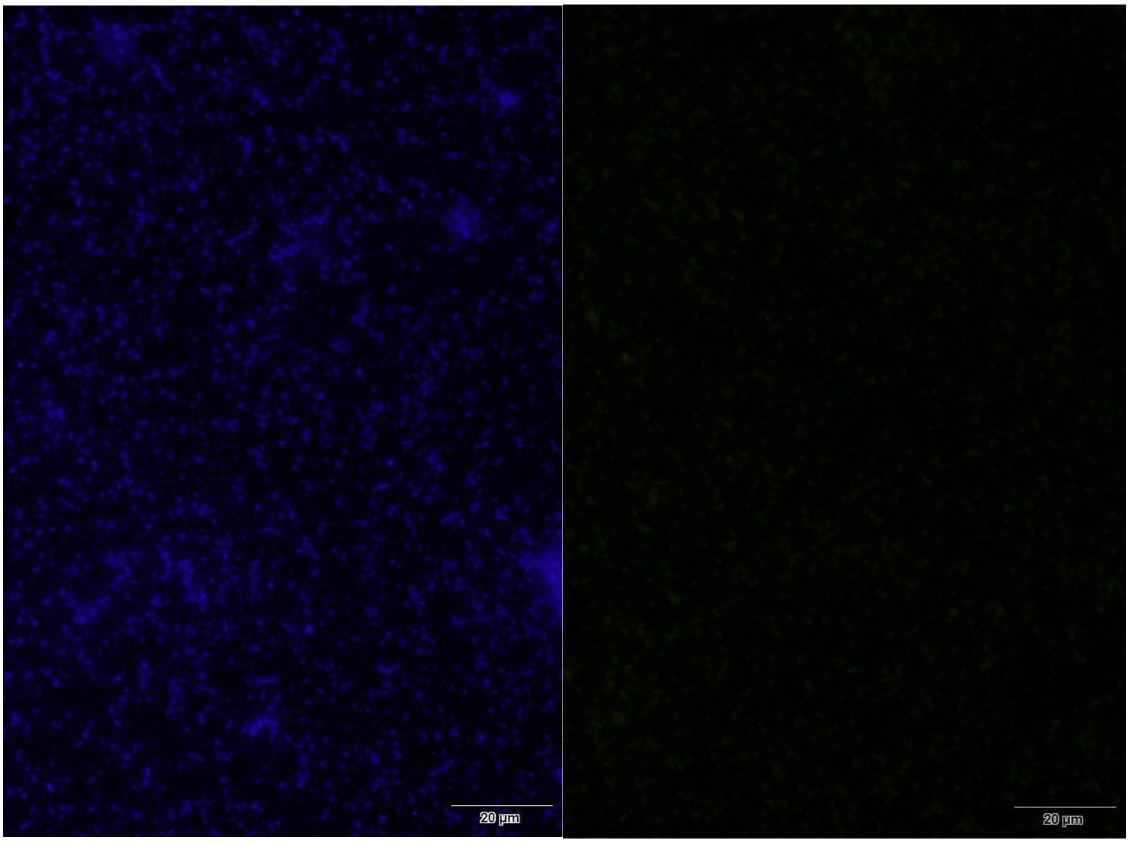

Prevotella corporis   CCUG15404

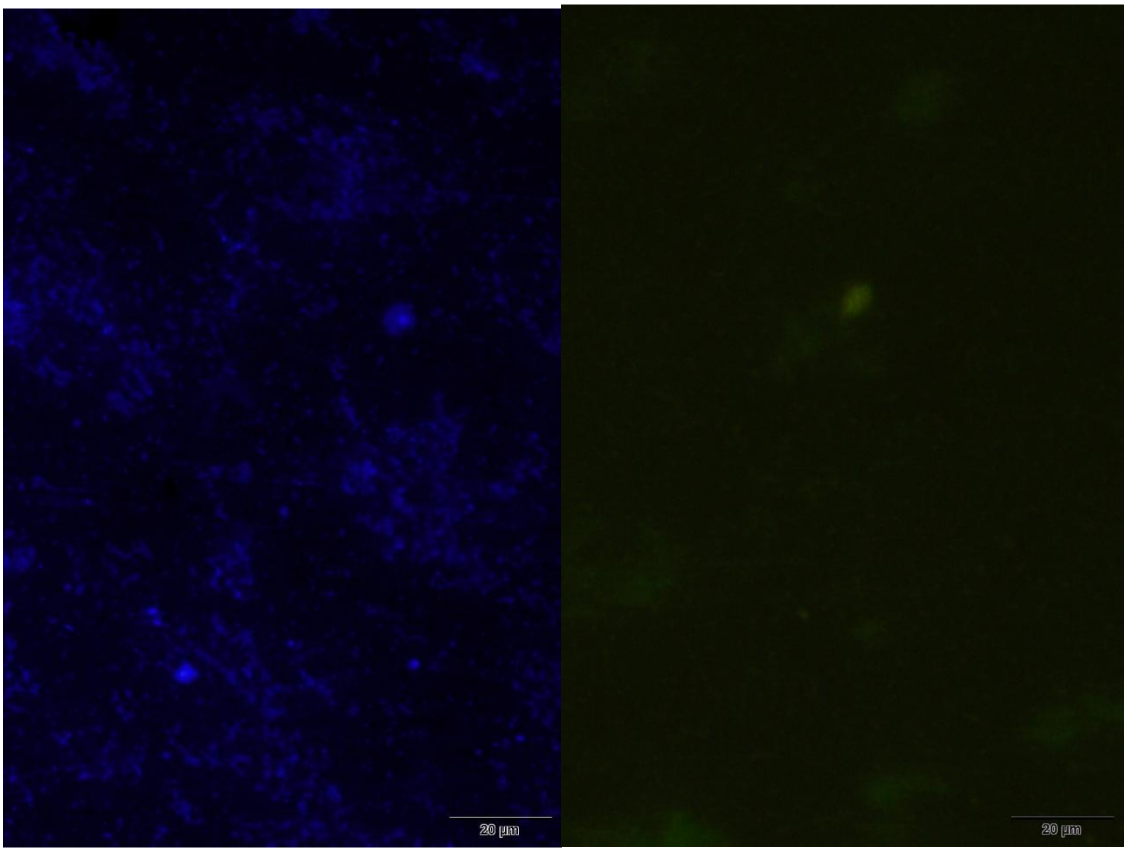

Prevotella dentalis   CCUG48288

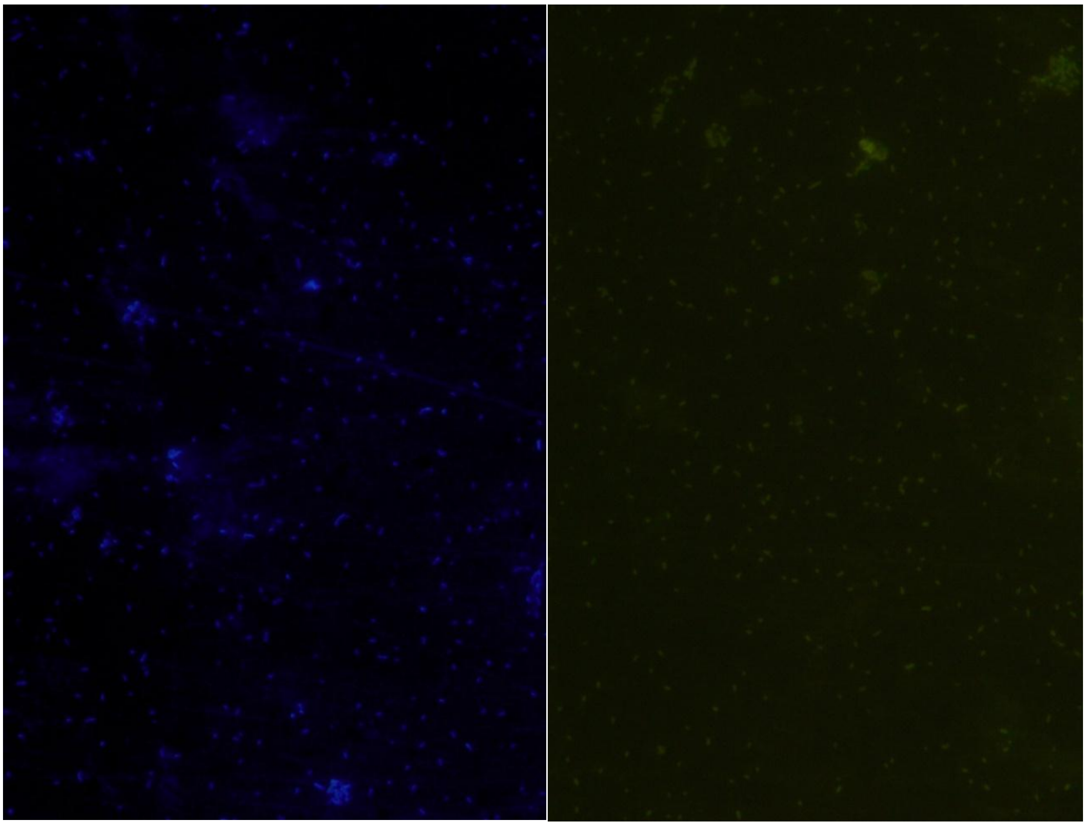

Prevotella denticola CCUG 29542T

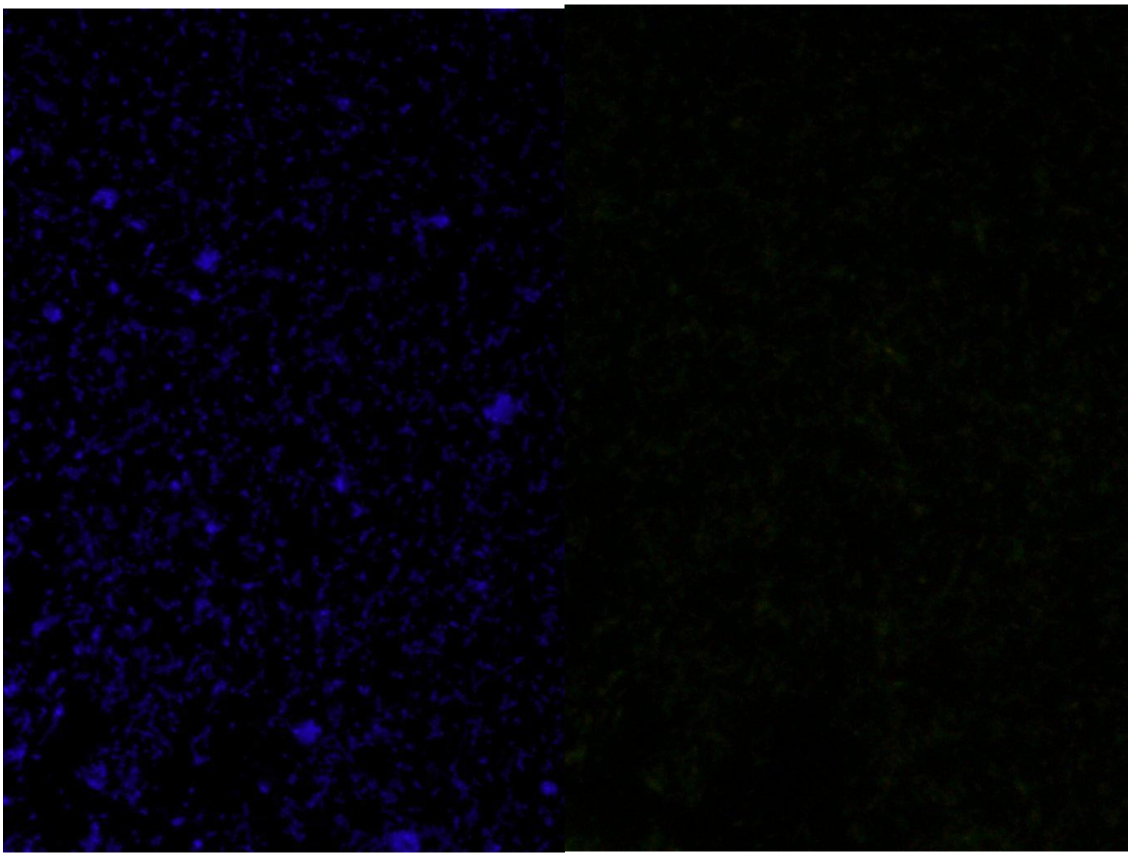

Prevotella disiens CCUG 59491

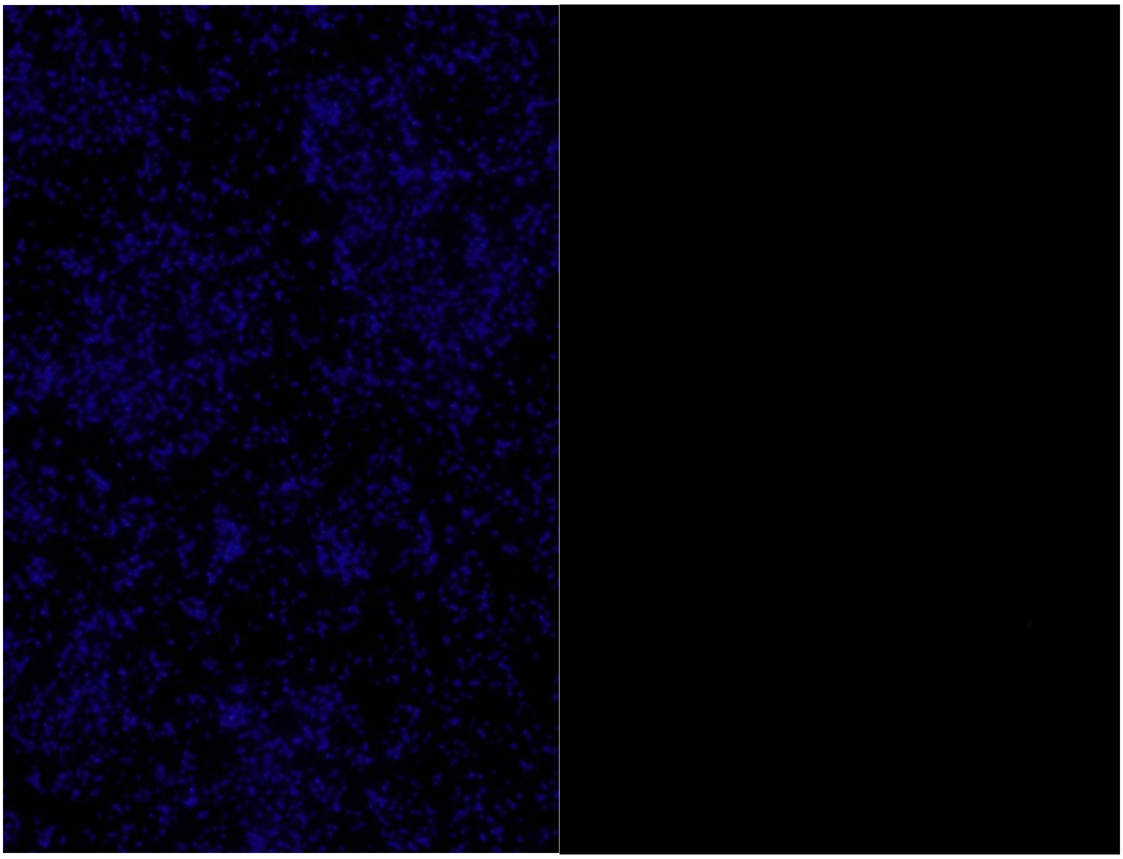

Prevotella fusca CCUG 57946

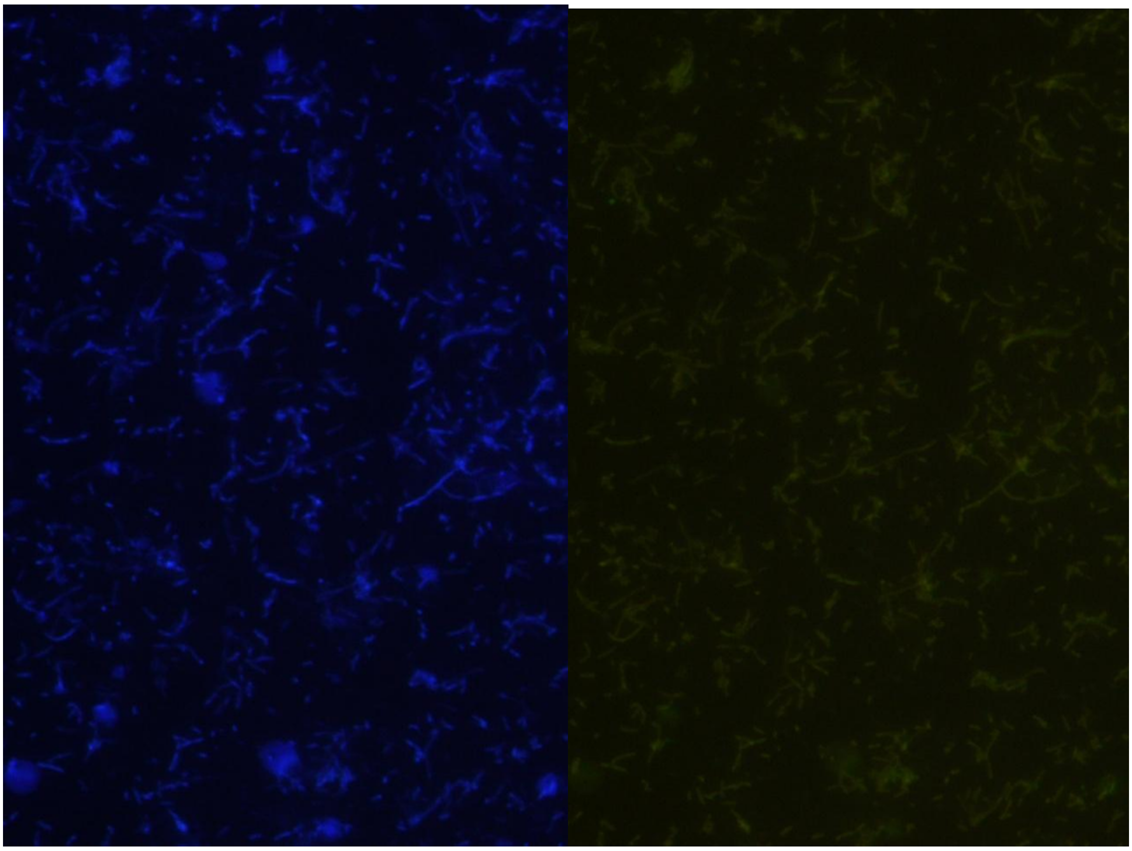

Prevotella histicola CCUG 55407

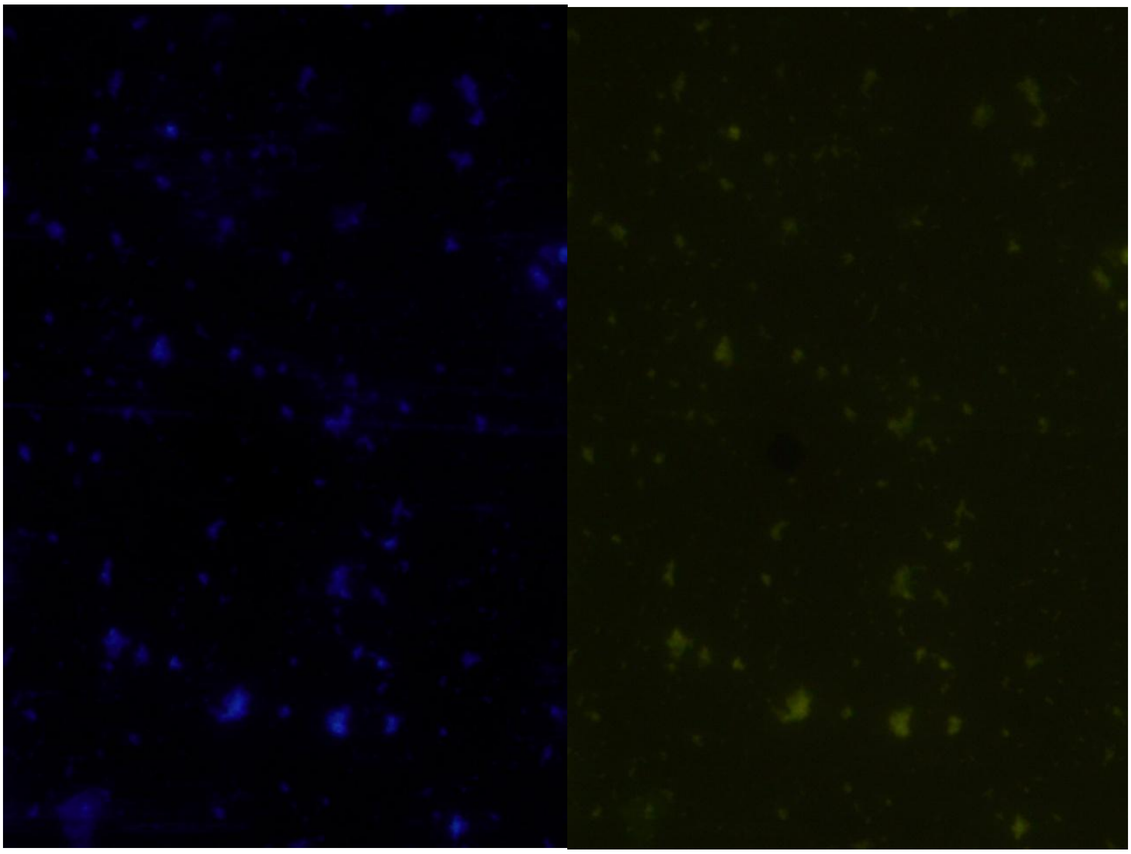

Prevotella illustrans CCUG 72806

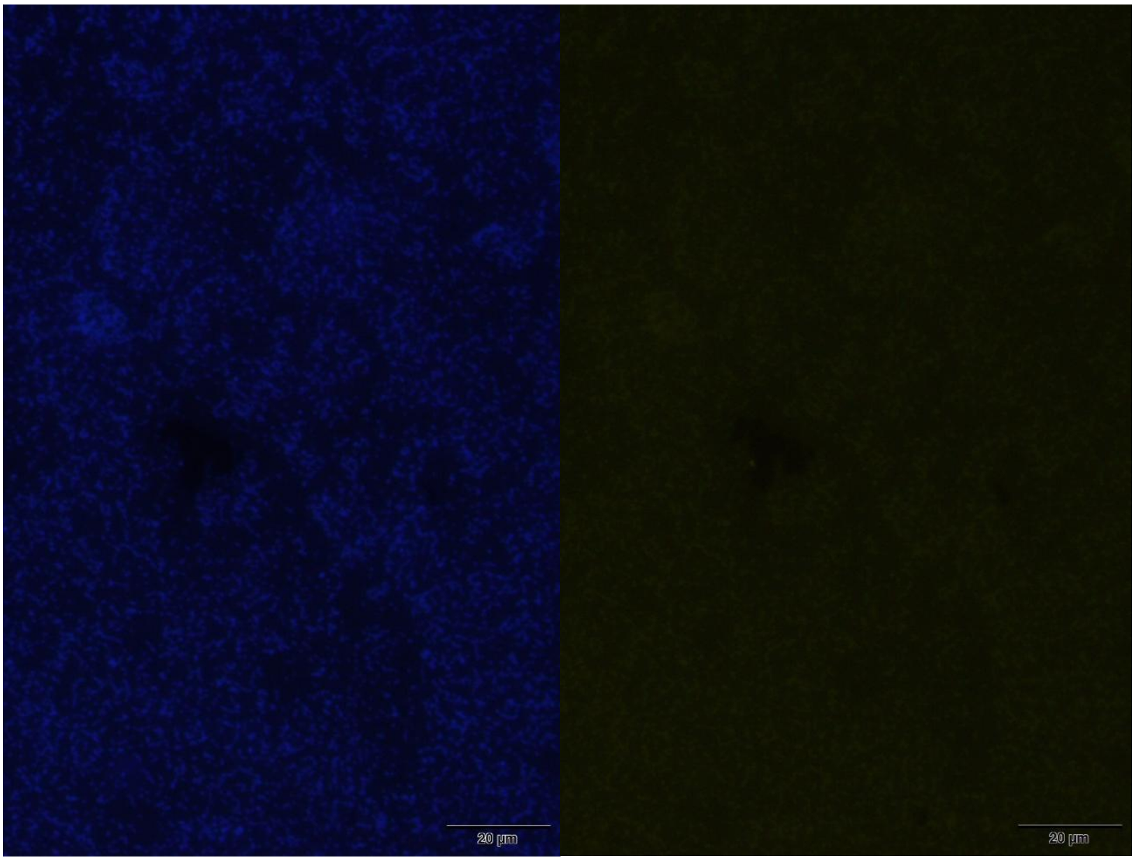

Prevotella imum CCUG 65911

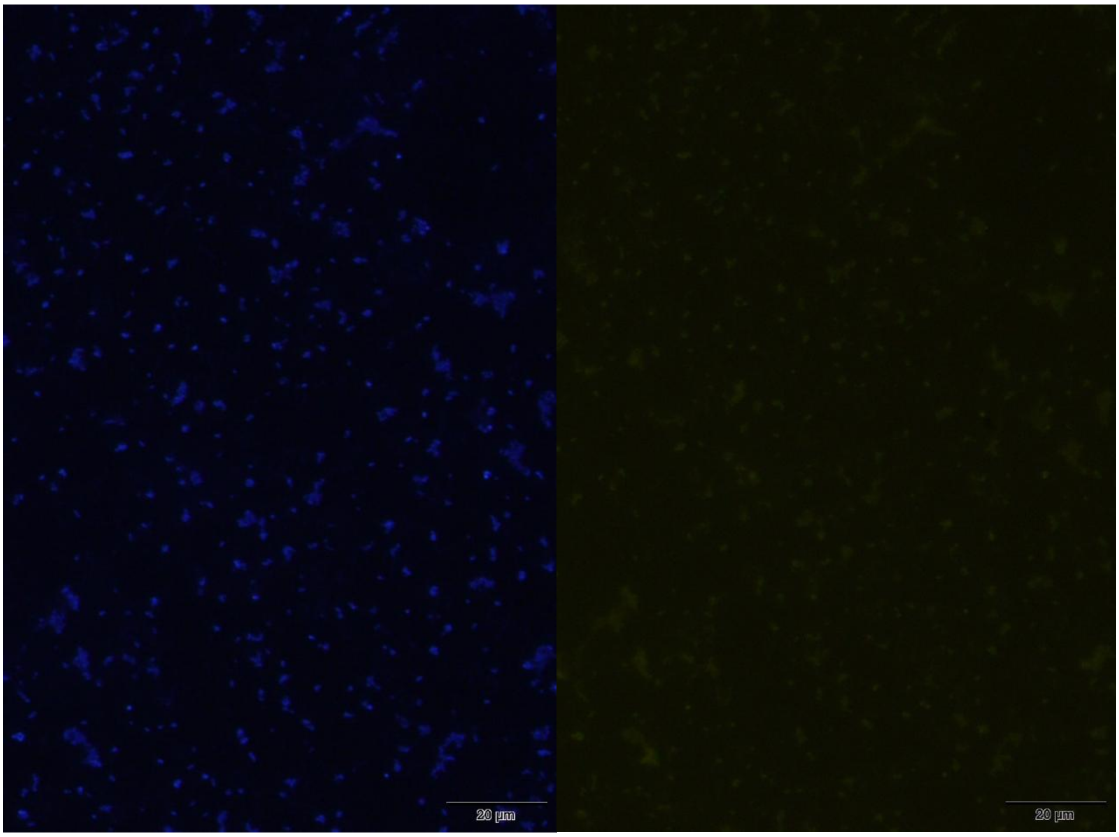

Prevotella intermedia CCUG 31410

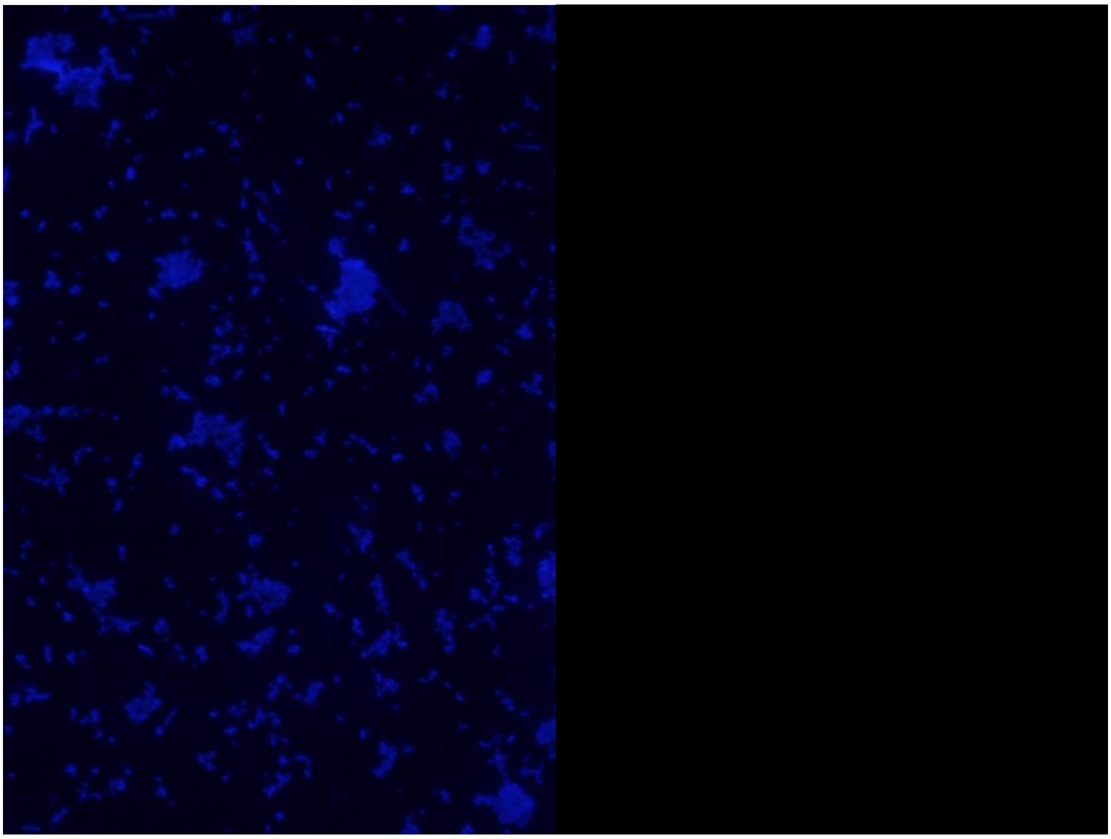

Prevotella jejuni CCUG 60371

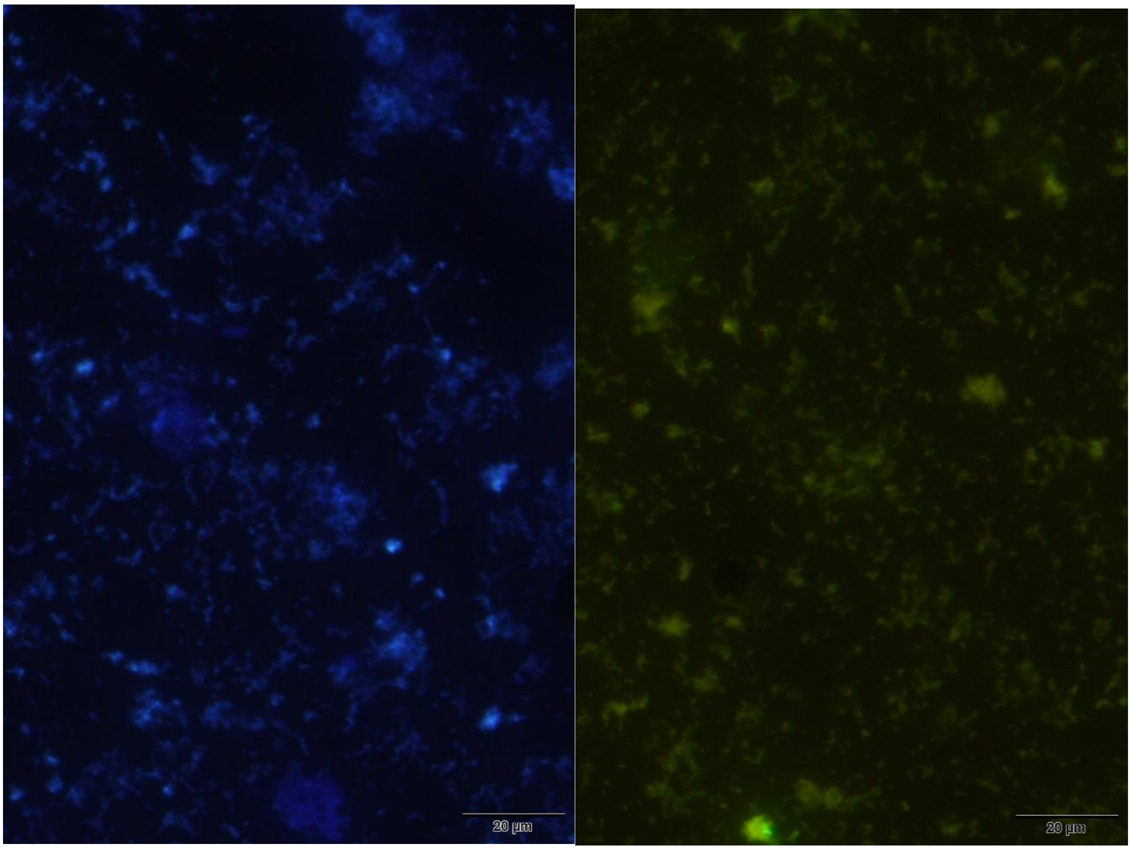

Prevotella melaninogenica CCUG 65141

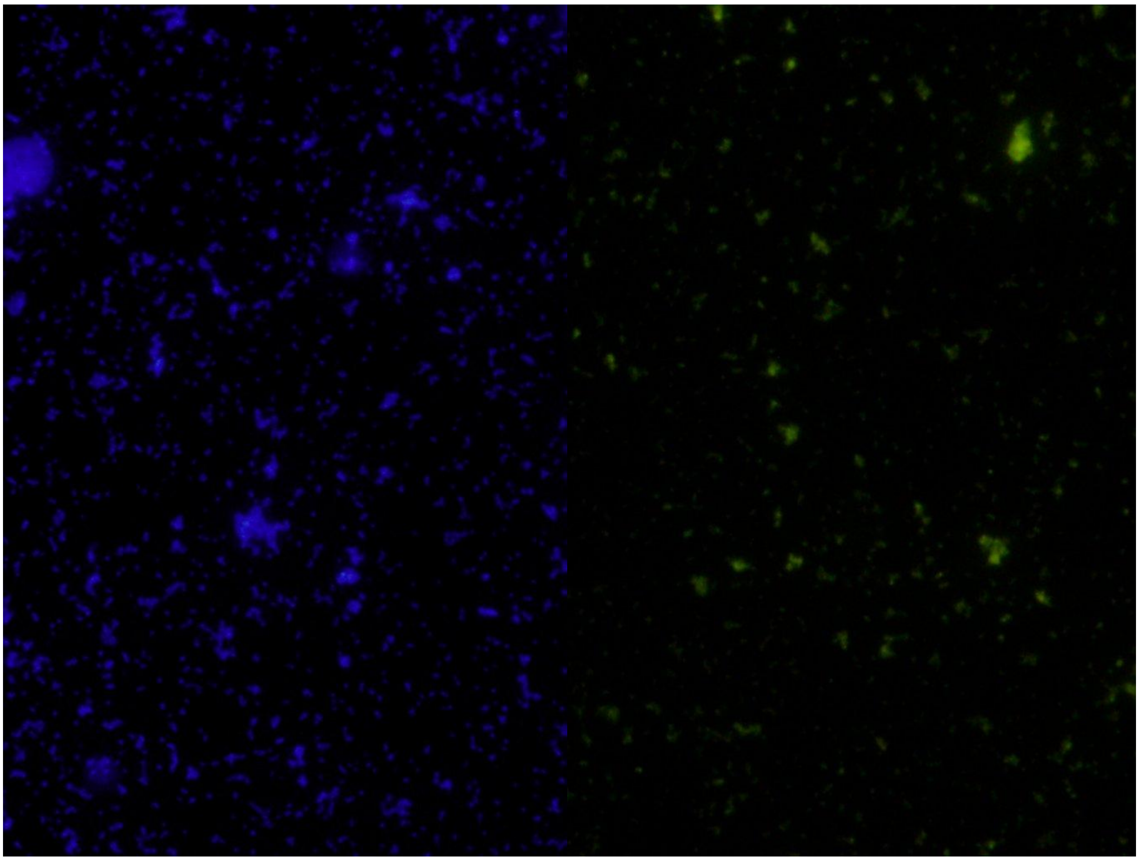

Prevotella micans CCUG 56105

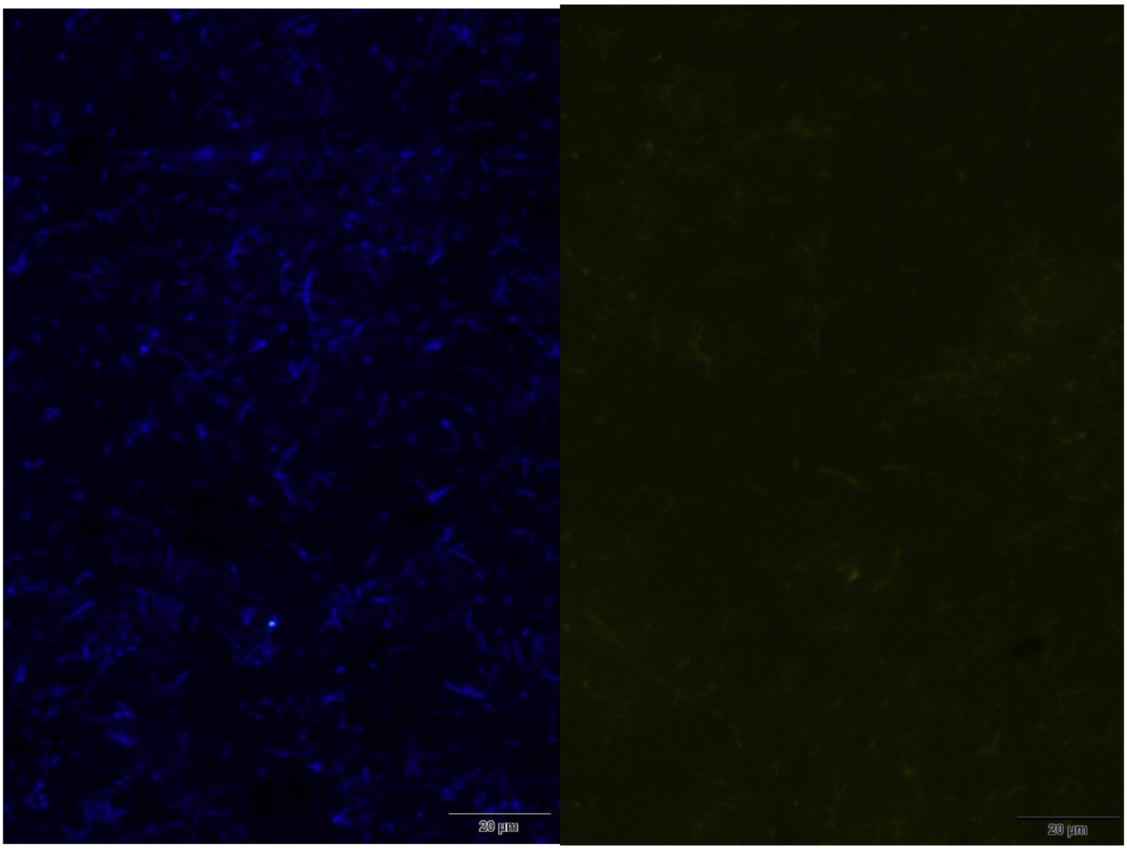

Prevotella multiformis CCUG 51937

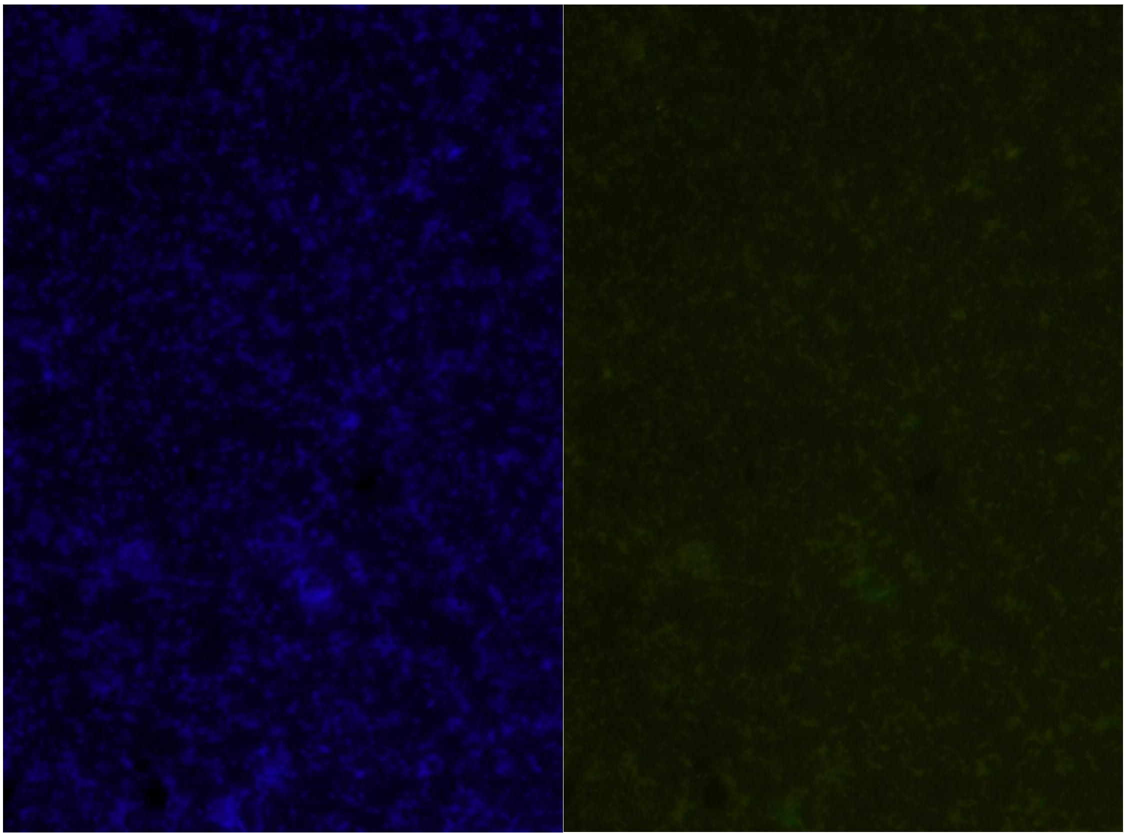

Prevotella nigrescens CCUG 25289

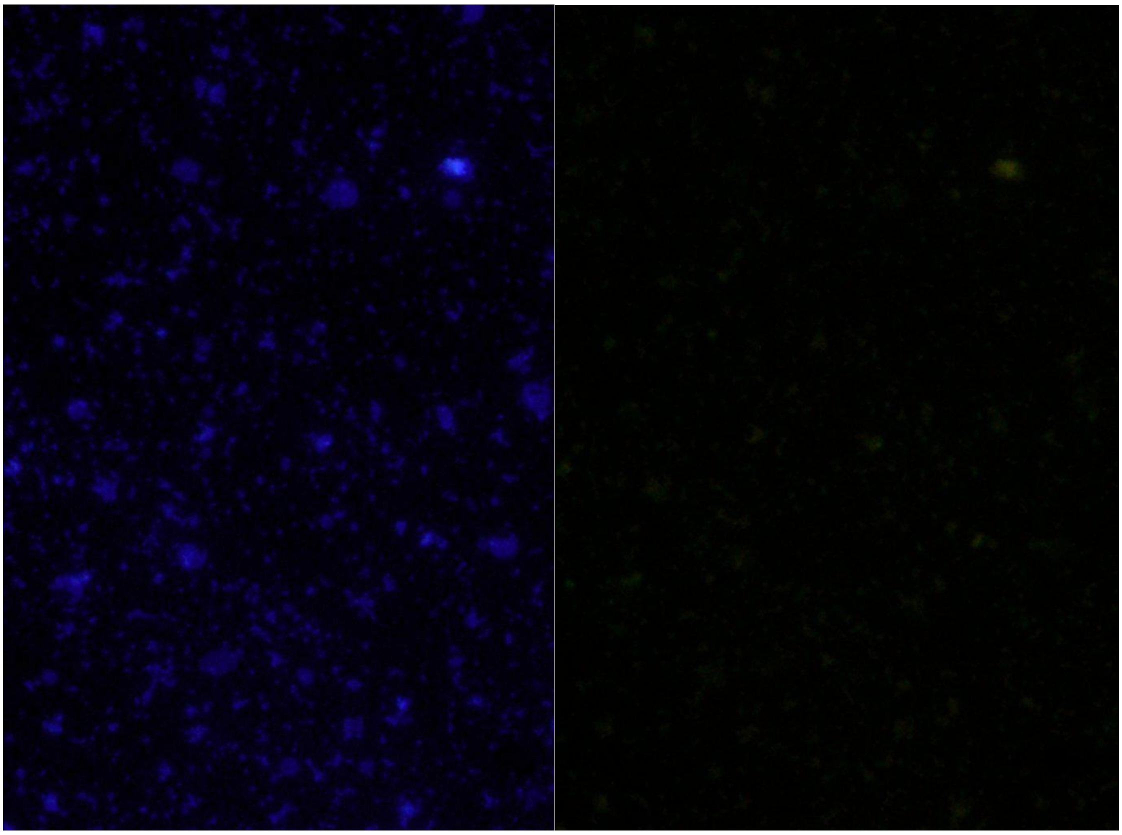

Prevotella pallens CCUG 39484

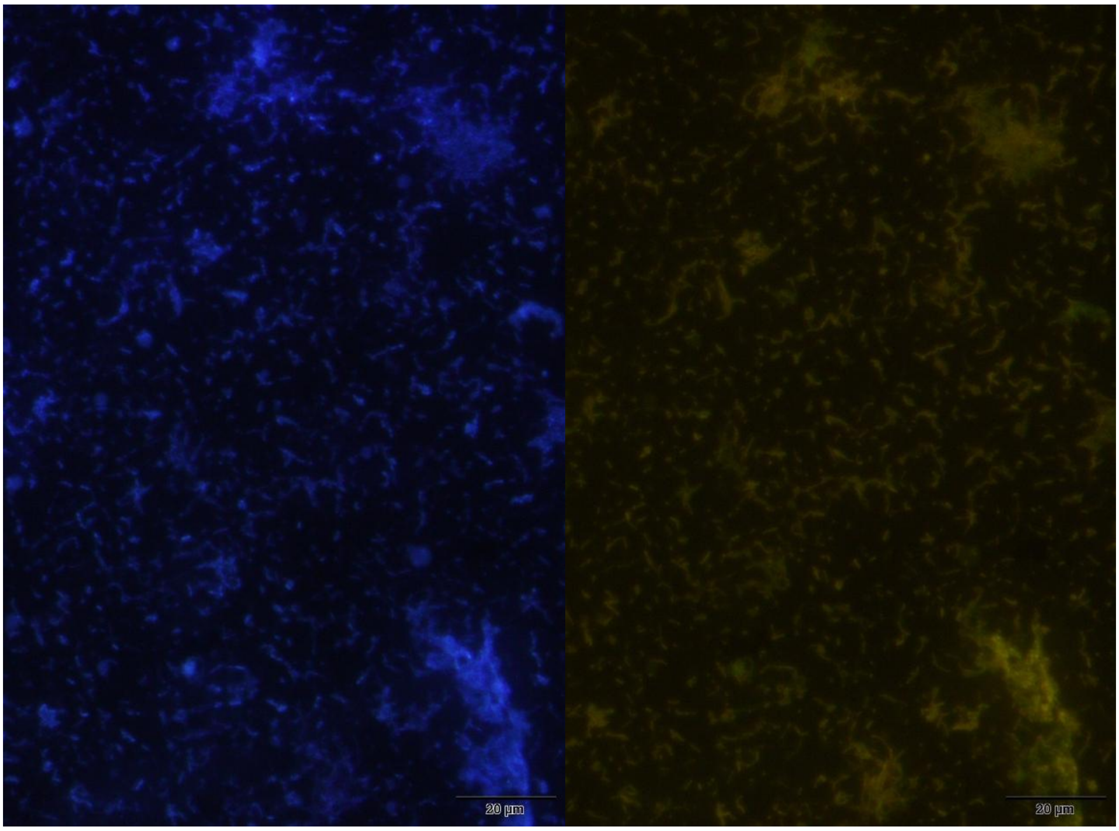

Prevotella scopos CCUG 57945

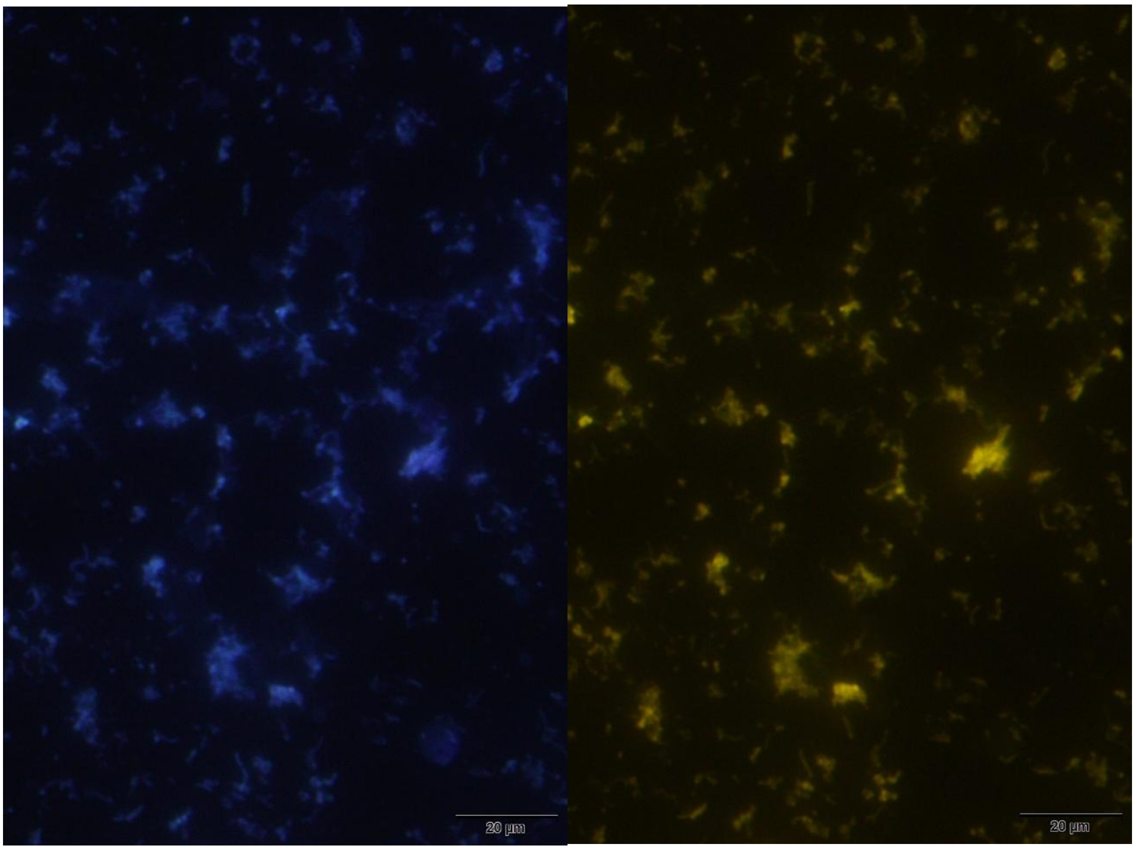

Prevotella timonensis CCUG 59487

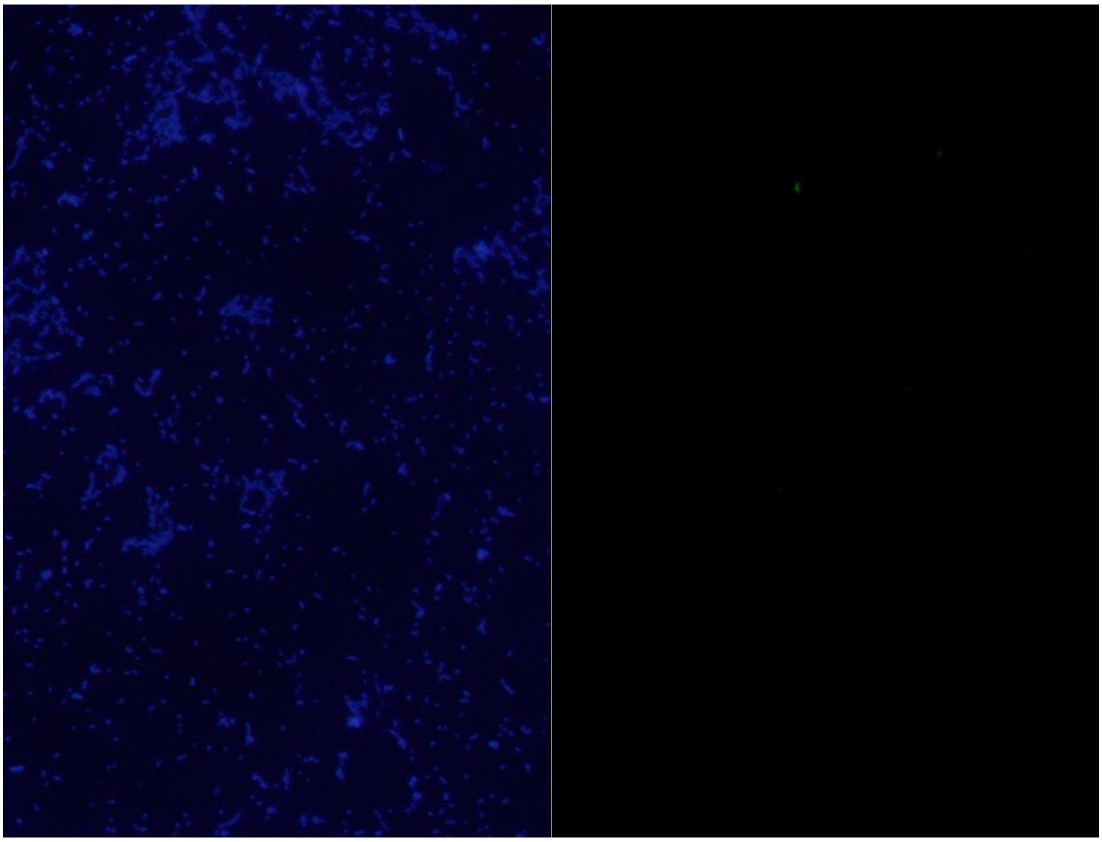

Prevotella veroralis CCUG 15422

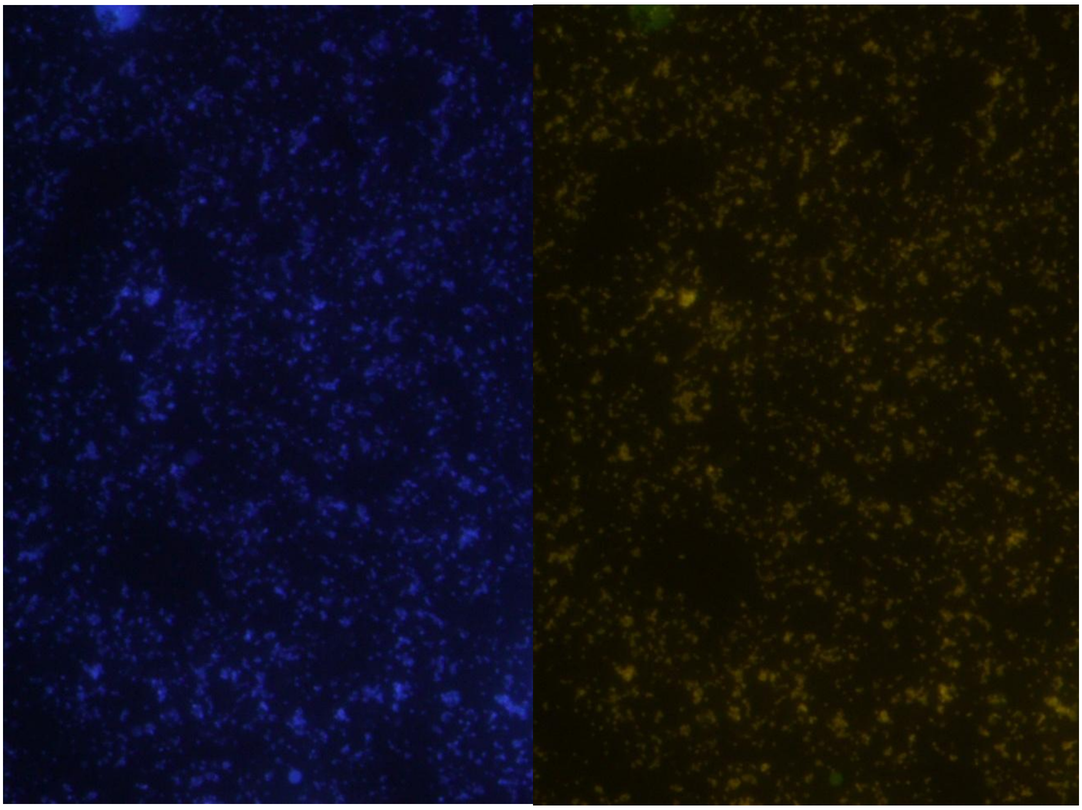

Prevotella vespertine CCUG 72808

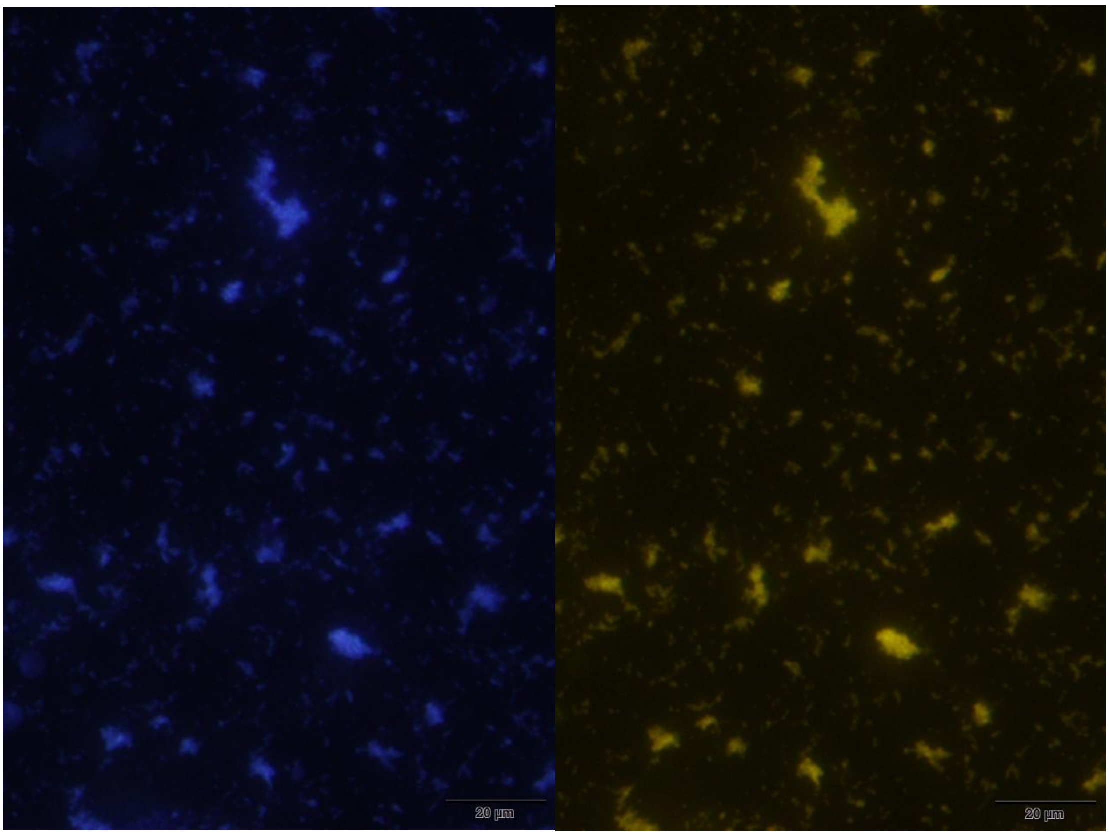

Supplementary Figure 3: Representative images of iBV case vaginal specimens hybridized with the *Prevotella* genus probe (left column) and the *P. bivia* probe (right column). Images taken at 60X magnification on the NanoZoomer S60.

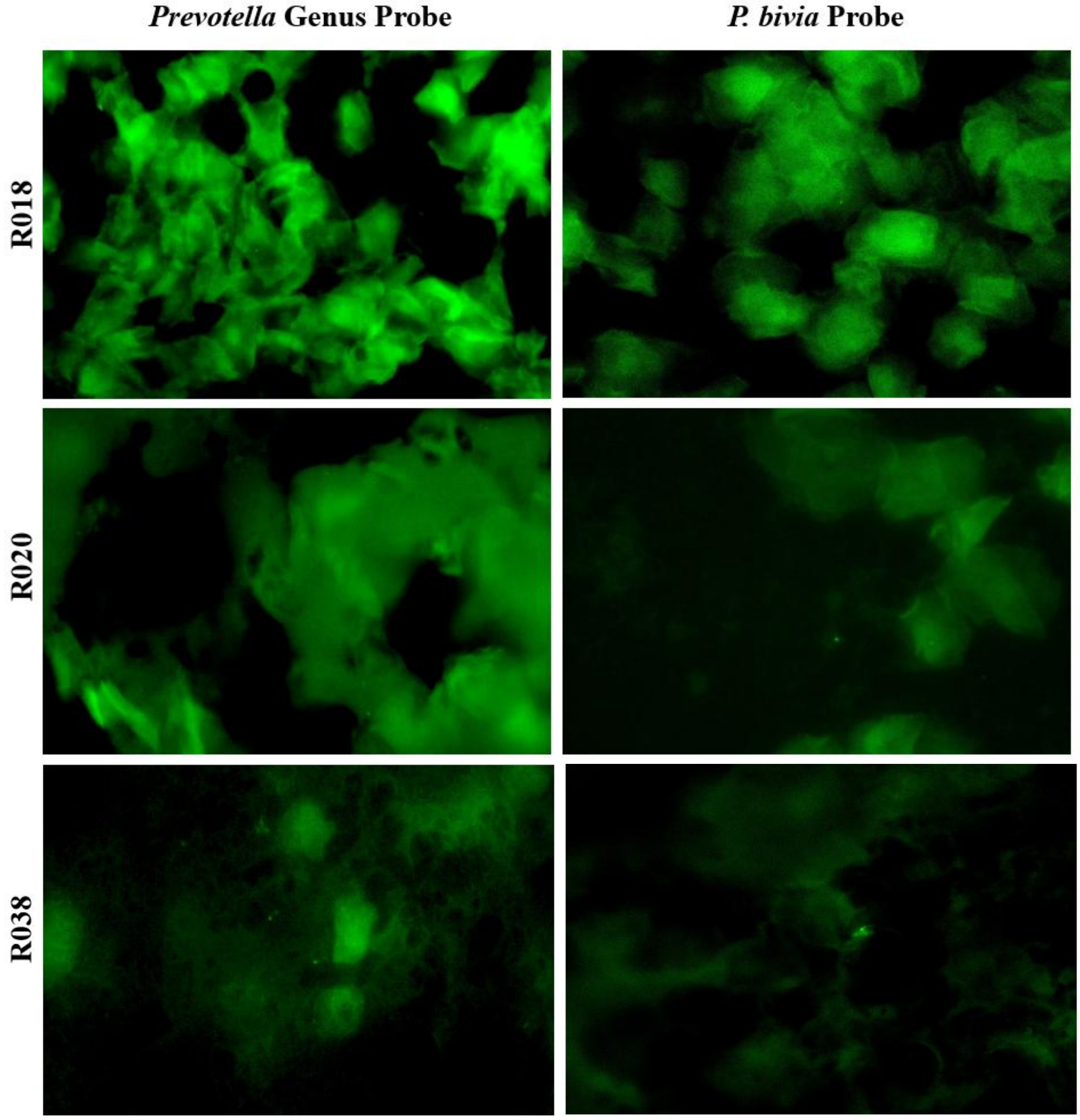

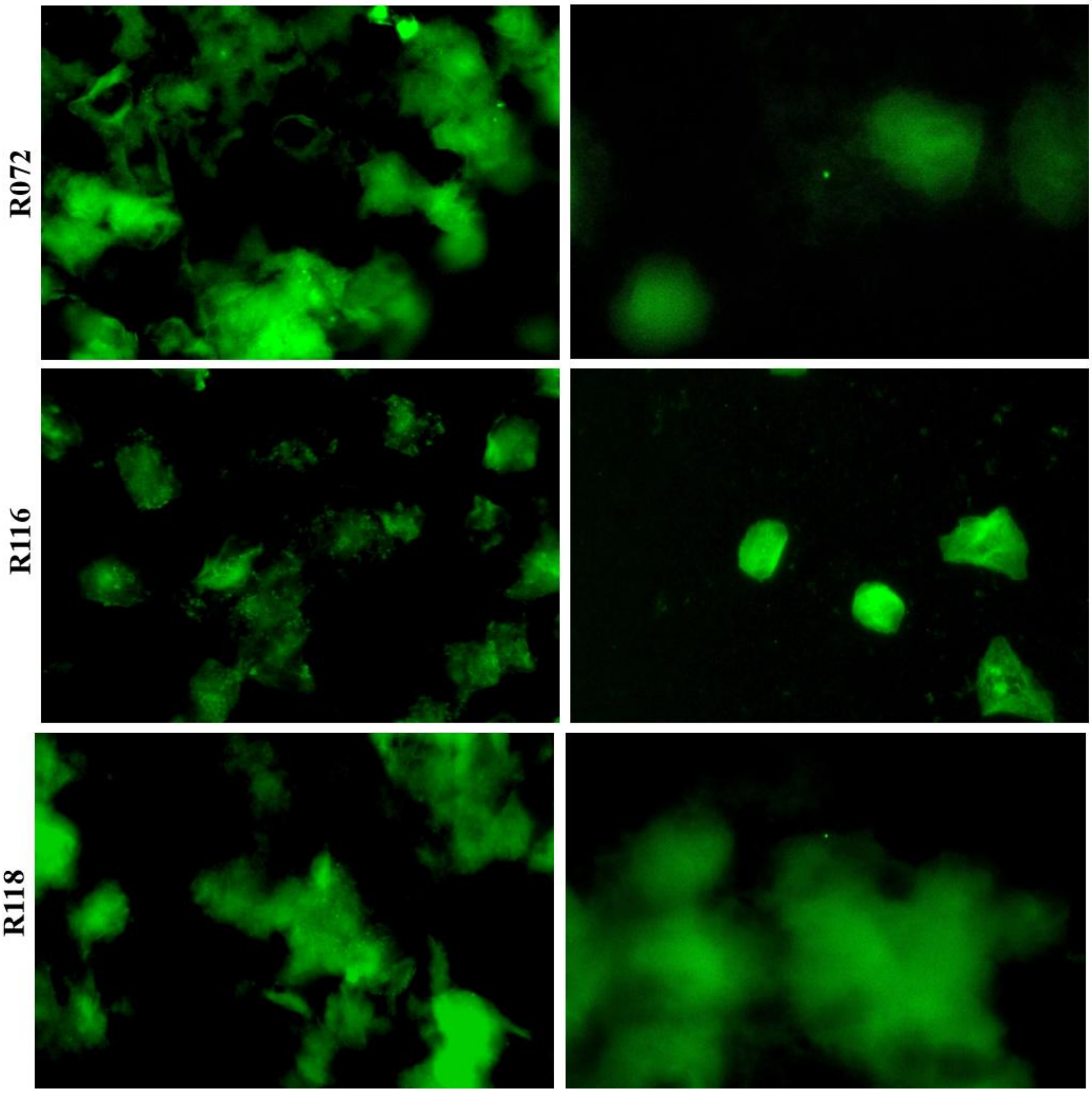
R072
R116
R118

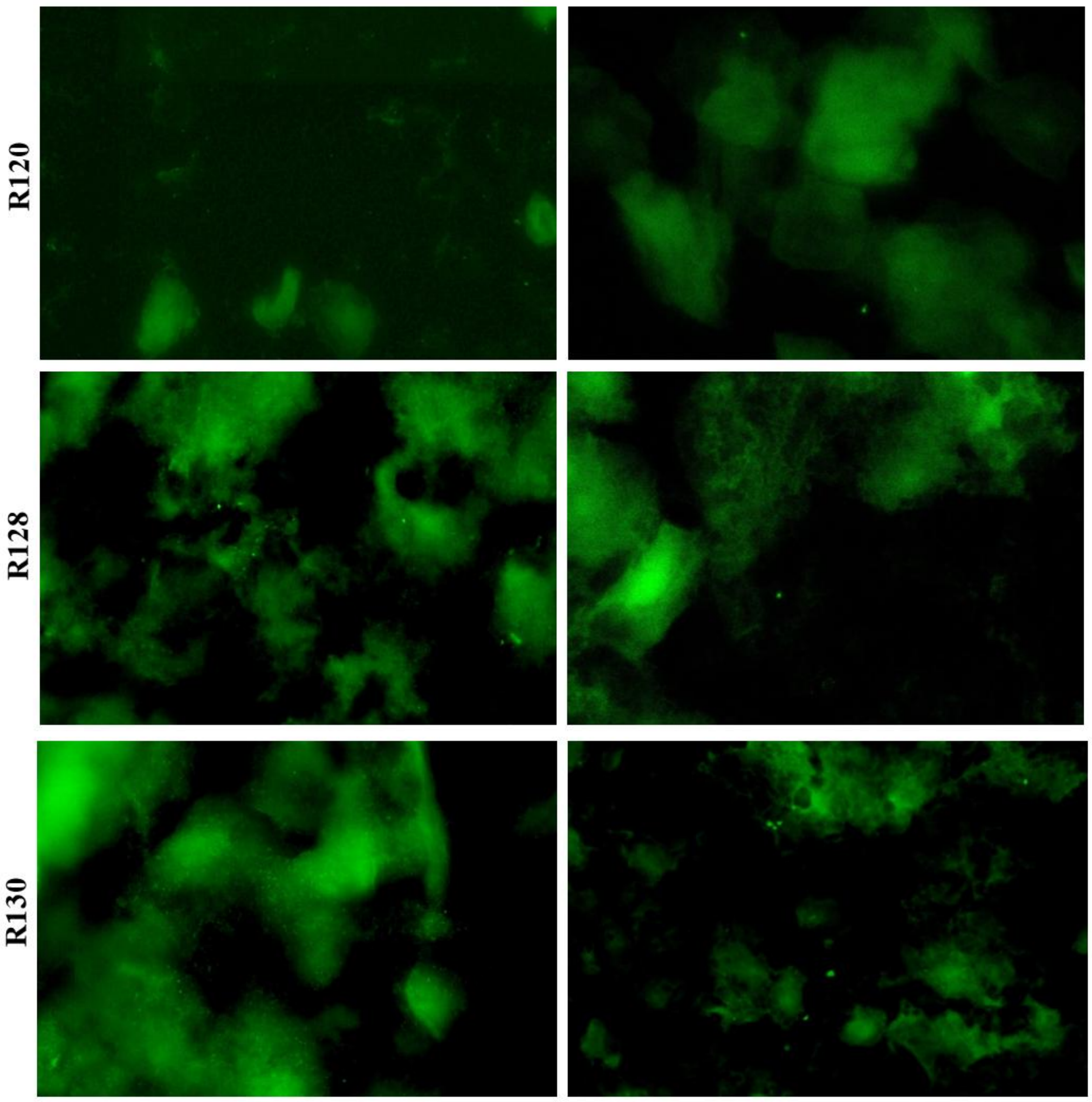
R120
R128
R130

Supplementary Figure 4: The following images show the fluorescence microscopy results for some of the species that present autofluorescence. These species were observed without any probe added to the hybridization solution to confirm if the autofluorescence signal is a result of non-specific hybridization of P. bivia PNA probe. The images were acquired on the DAPI filter (left image, blue) and FITC filter (right image, green), sensitive to the Alexa fluor 488, with a magnification 400x.

Actinomyces urgenitalis CCUG 44038

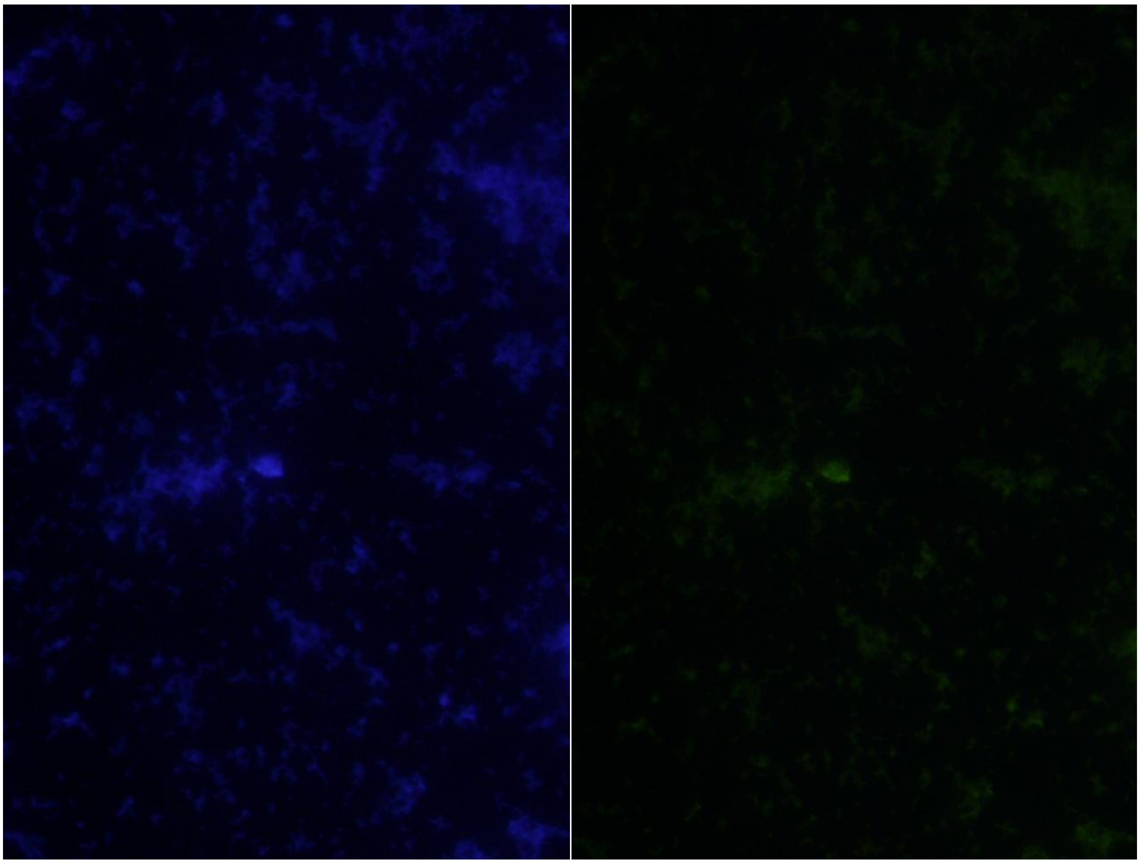

Bacillus firmus UM034

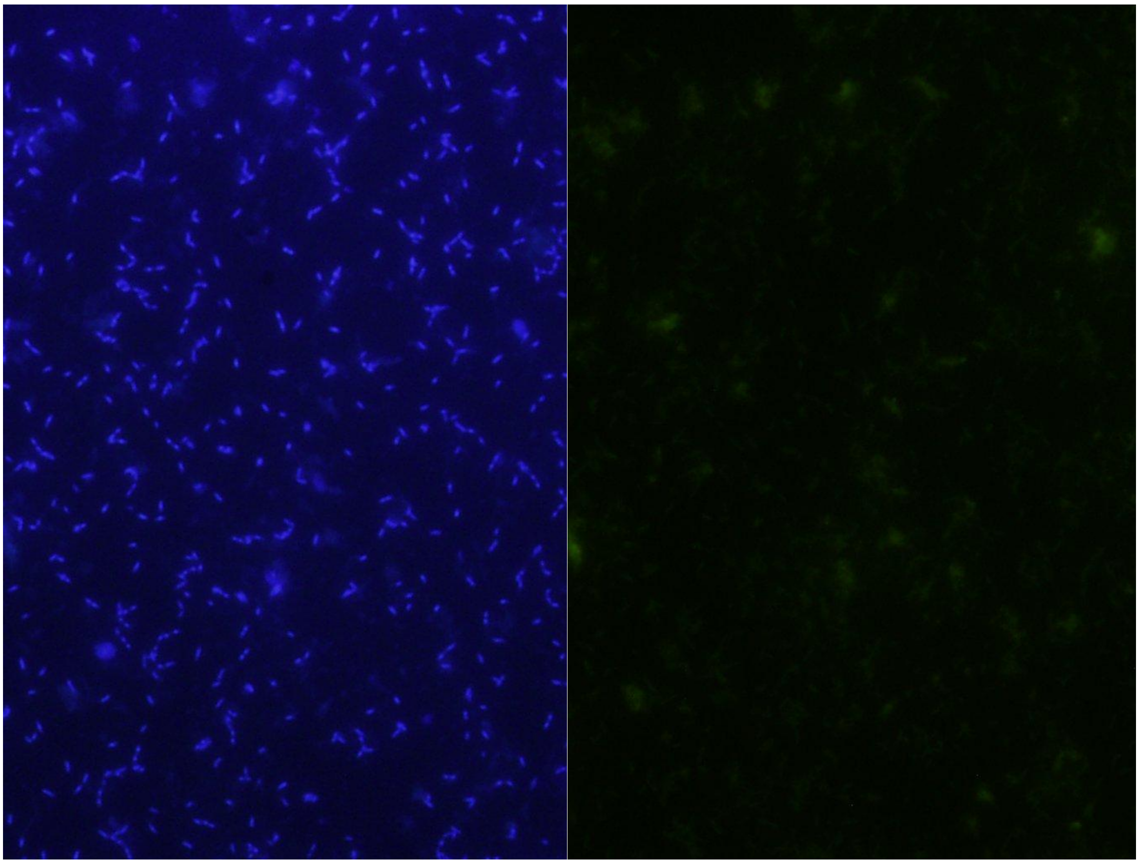

Bifidumbacterium bifidum CCUG 59492

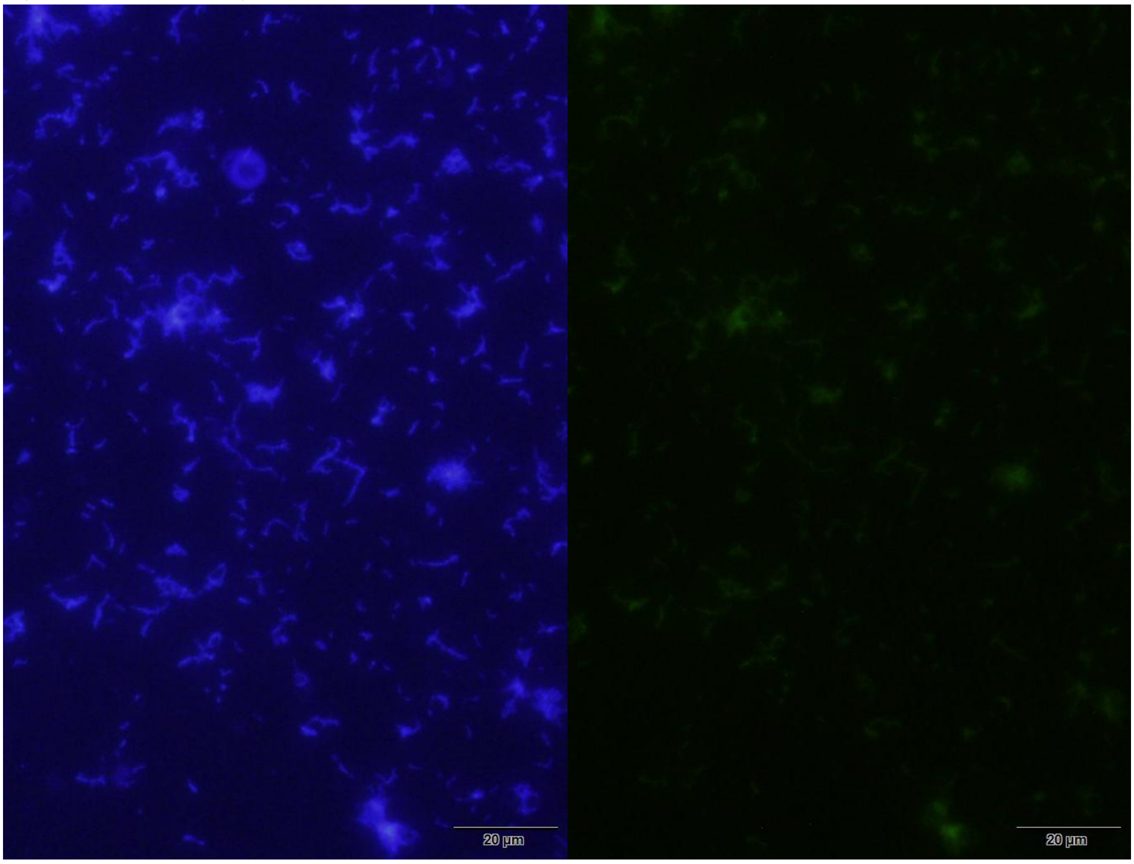

Brevibacterium ravenspurgense CCUG 42923

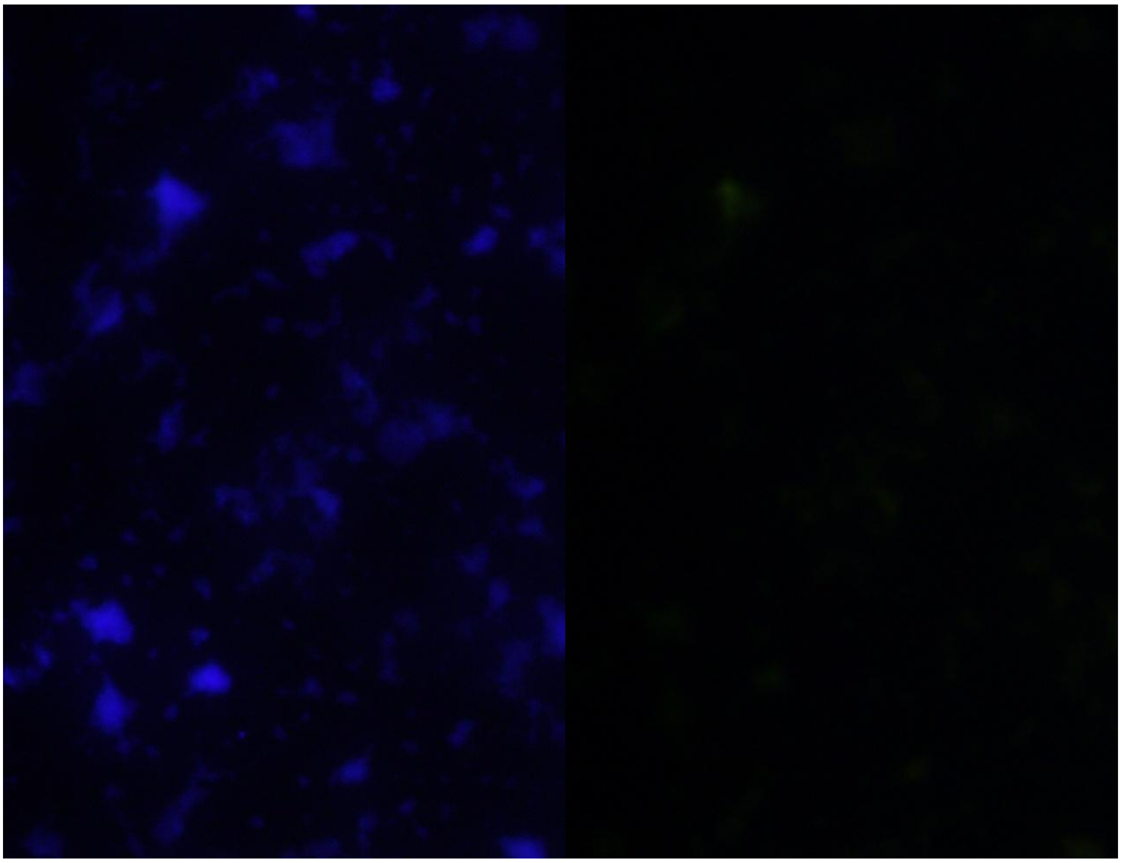

Corynebacterium tuscaniense UM137

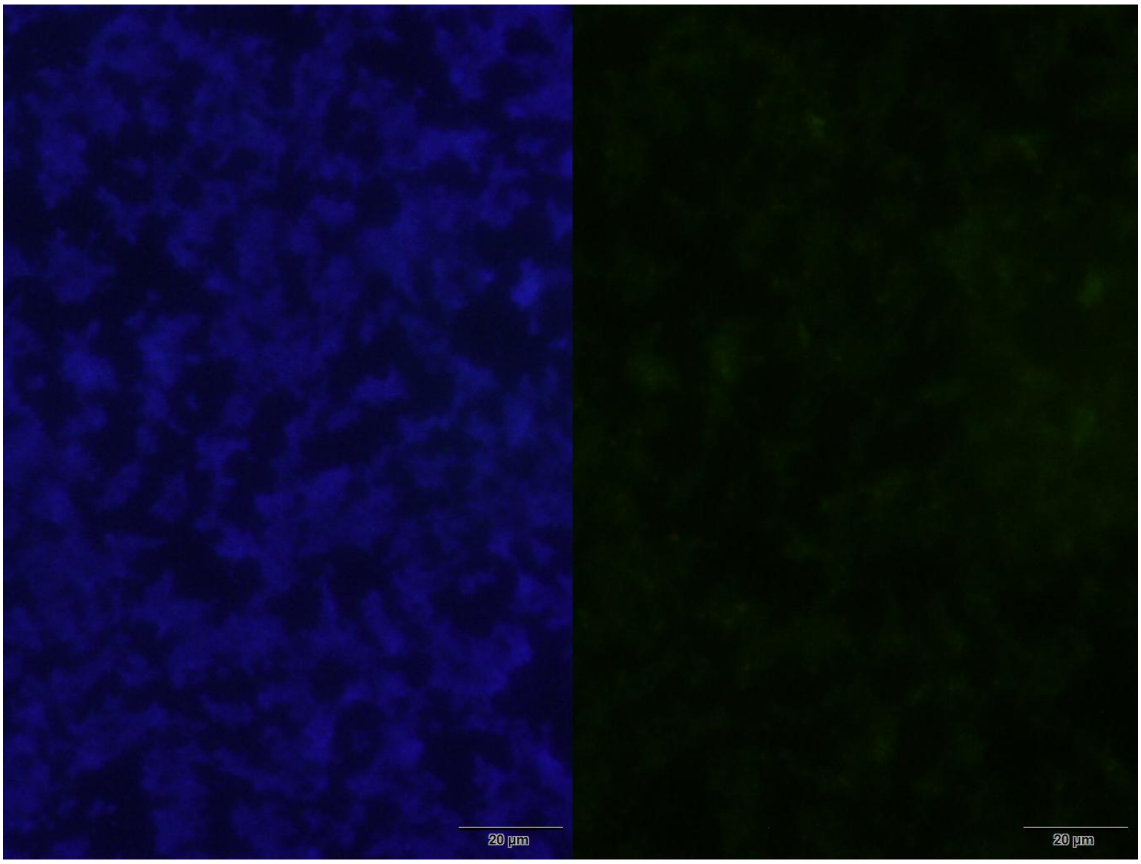

Enterococcus faecalis UM035

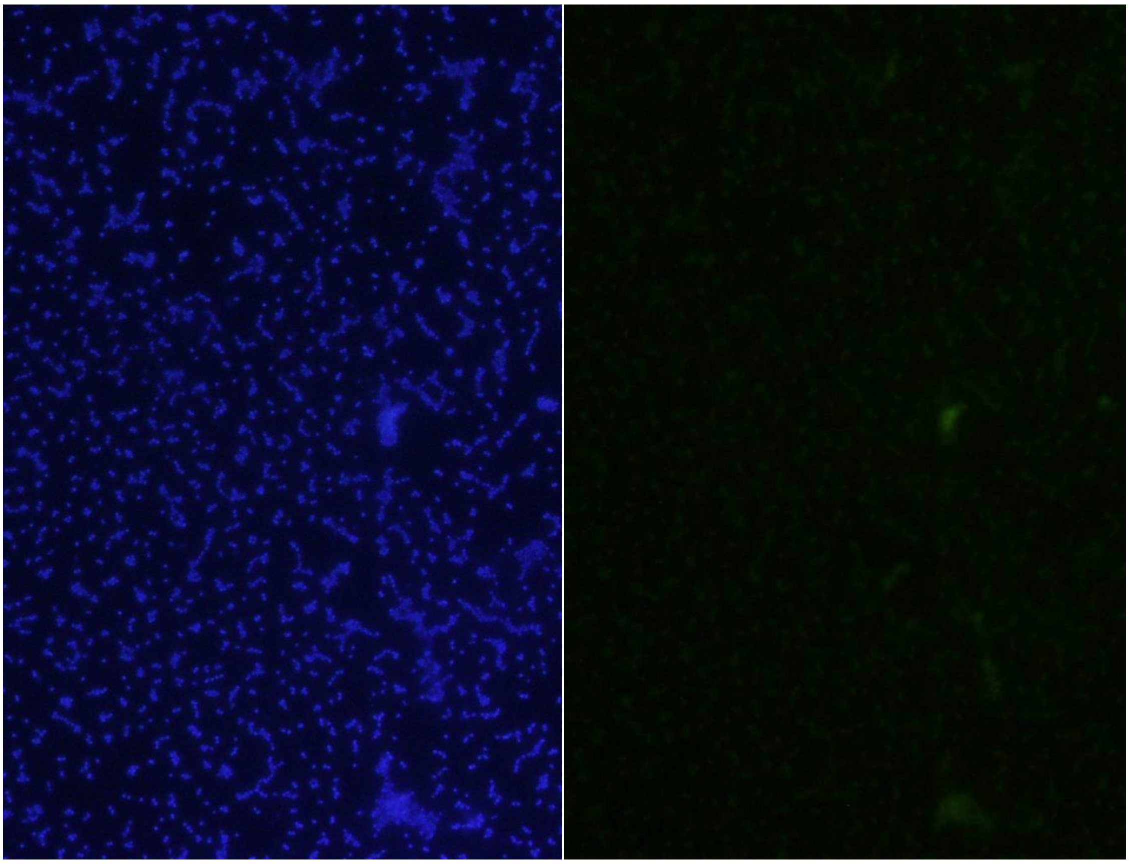

Fannyhessea vaginae ATCC BAA-55

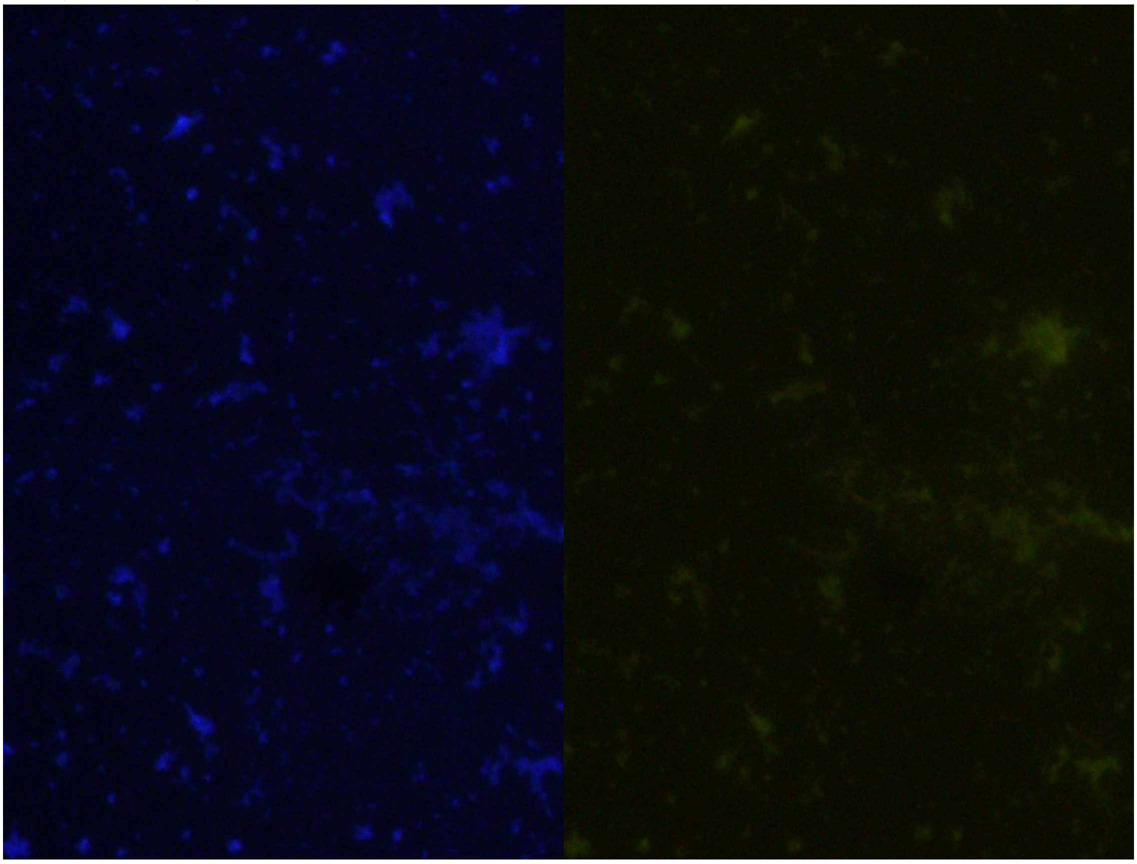

Gardnerella leopoldii UM034

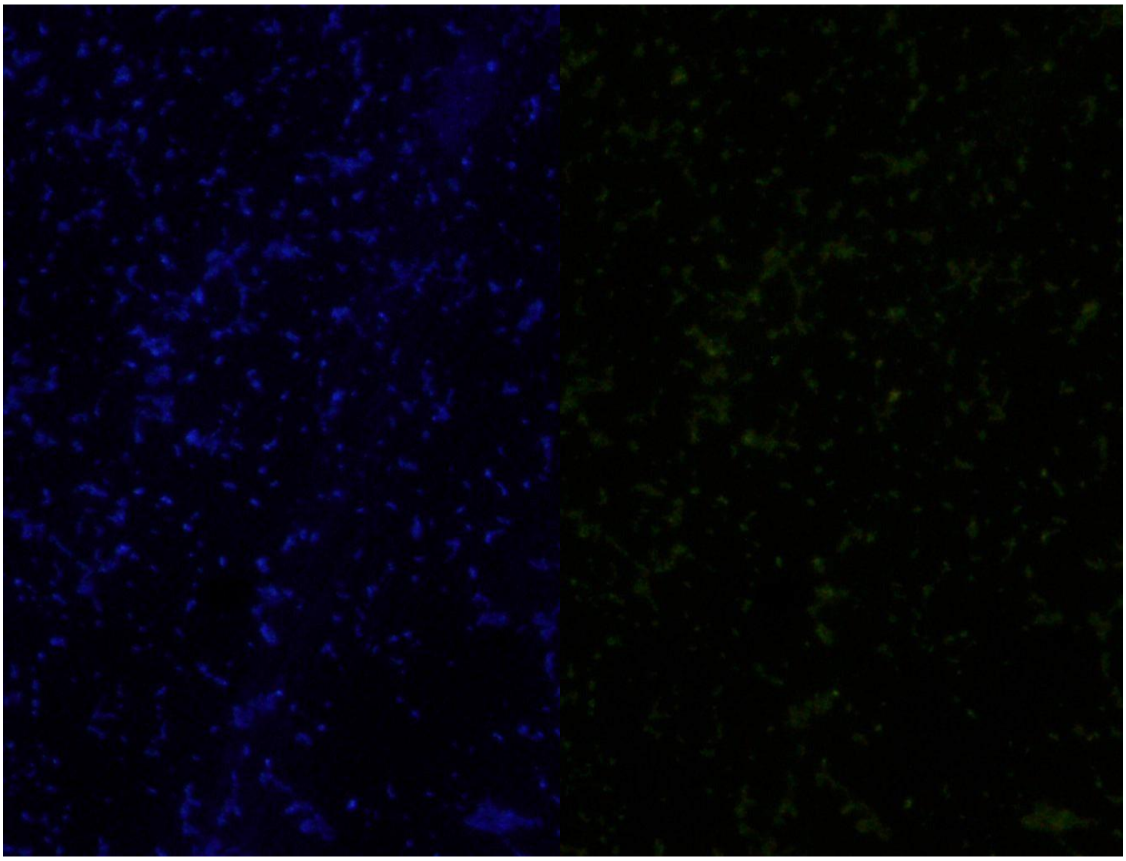

Gardnerella vaginalis ATCC 14018

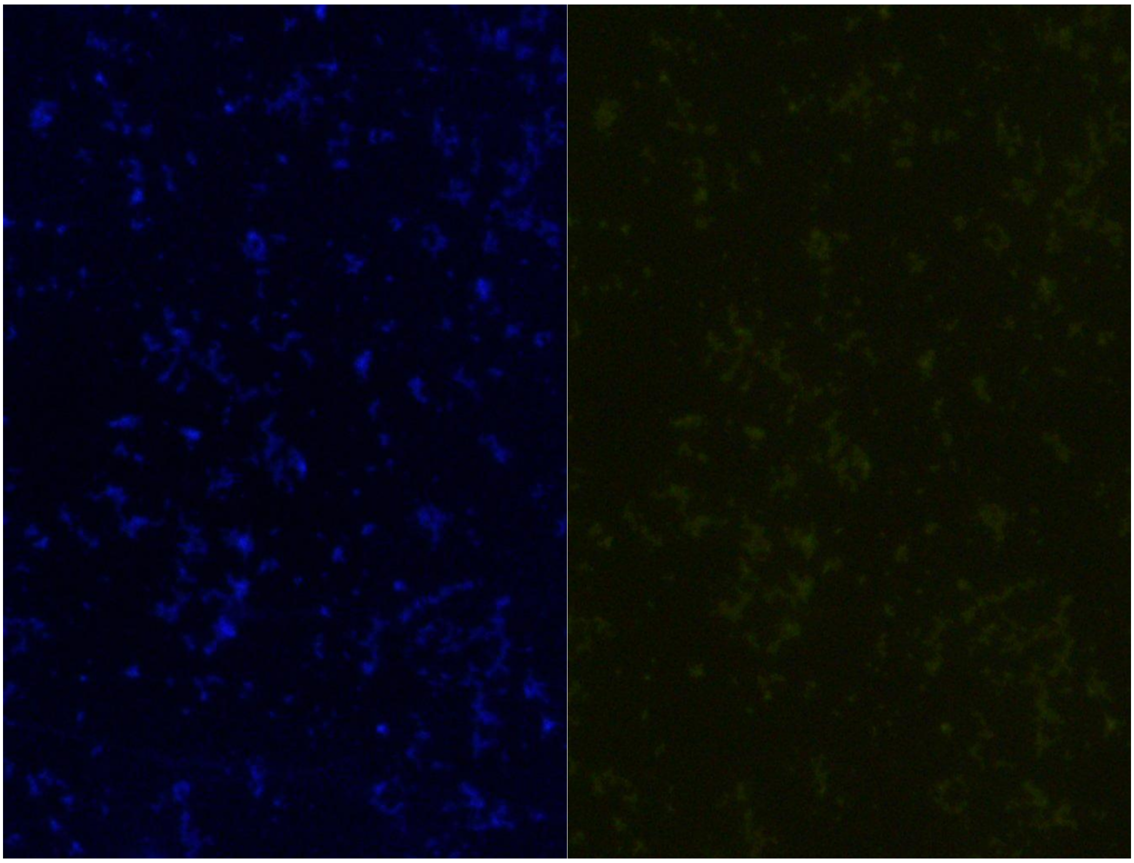

Lactobacillus gasseri ATCC 9857

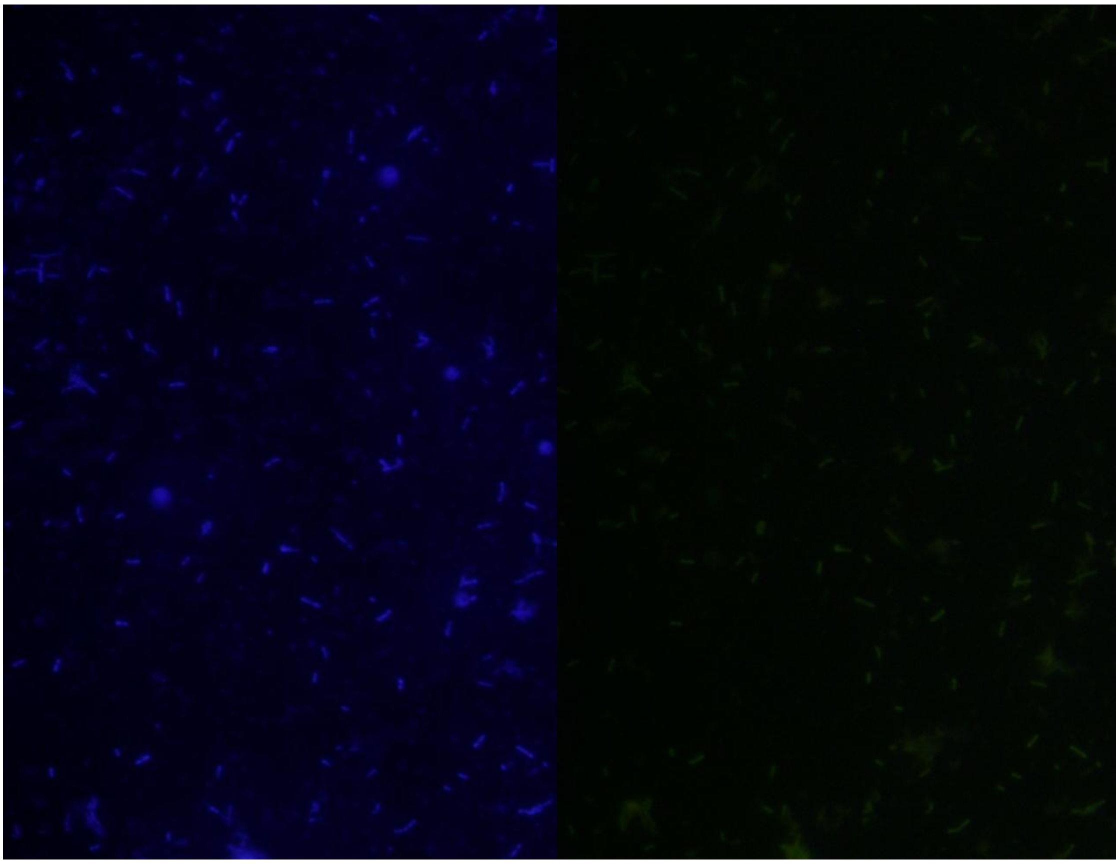

Lactobacillus rhamnosus CECT 288

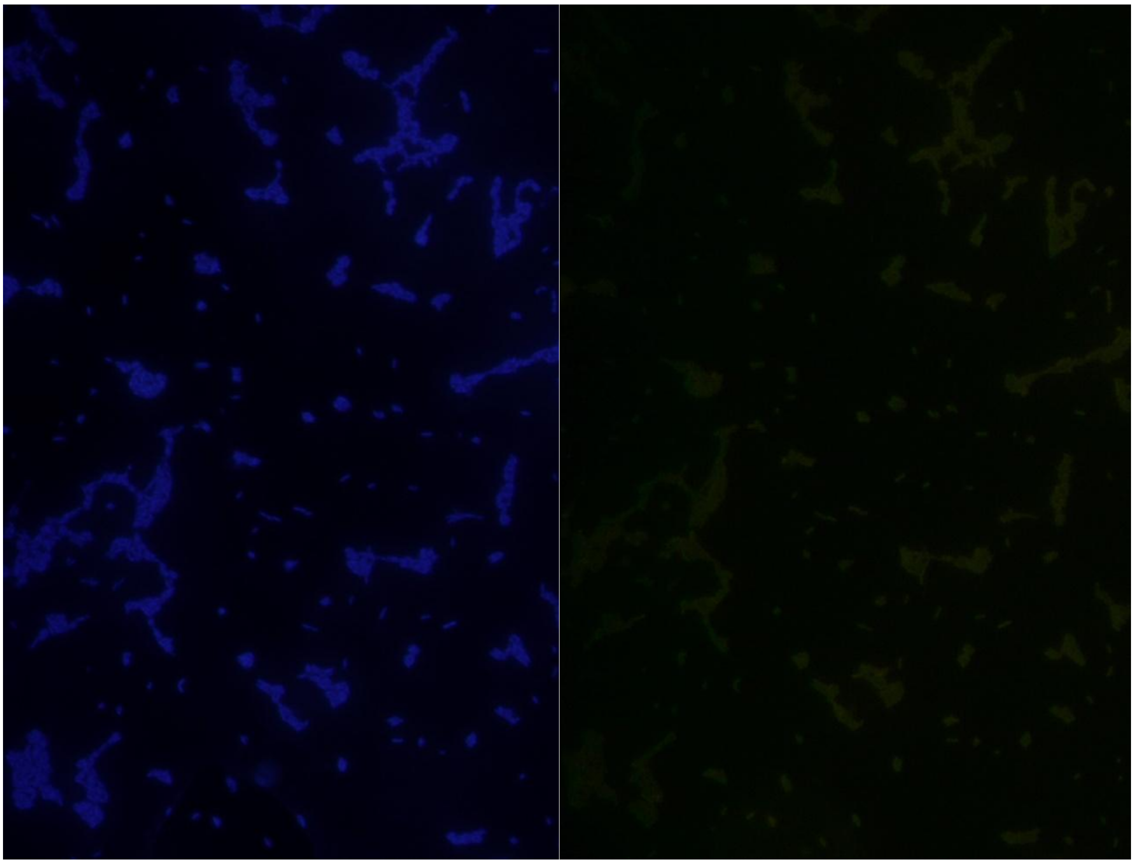

Lactobacillus vaginalis UM062

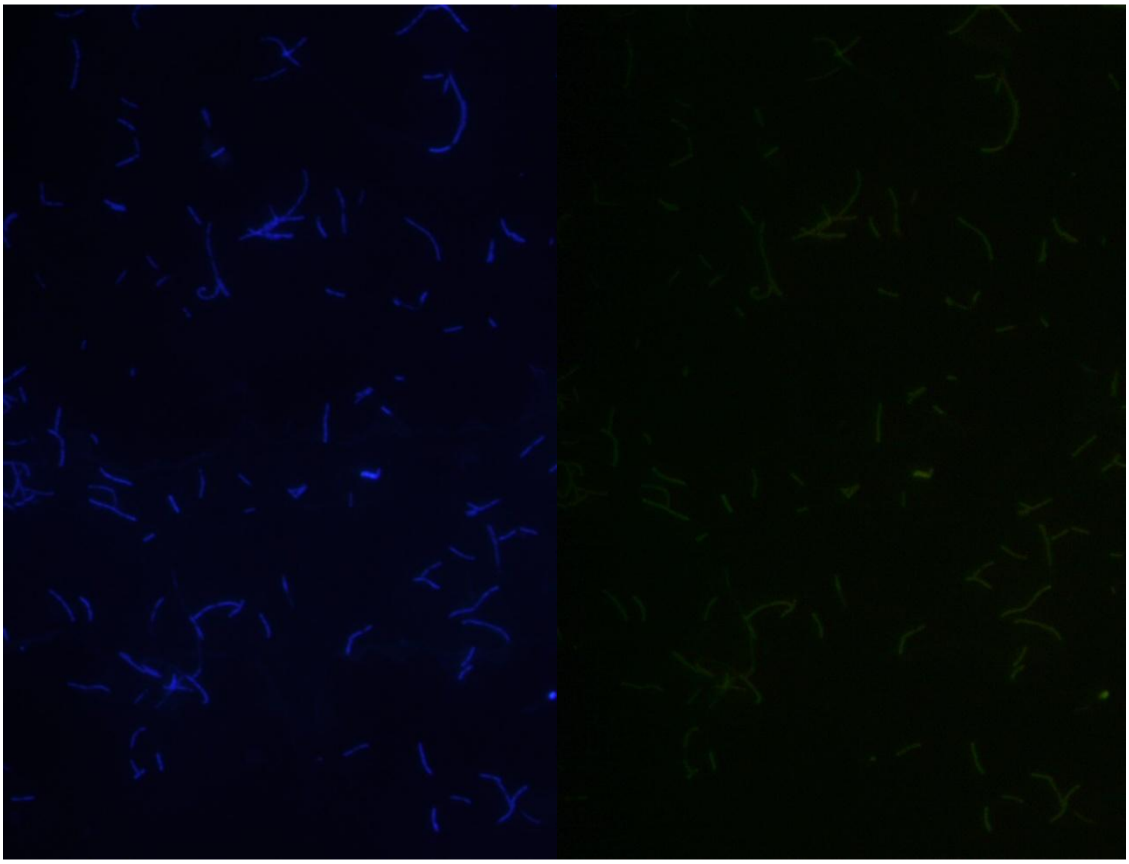

Nosocomiicoccus ampullae UM121

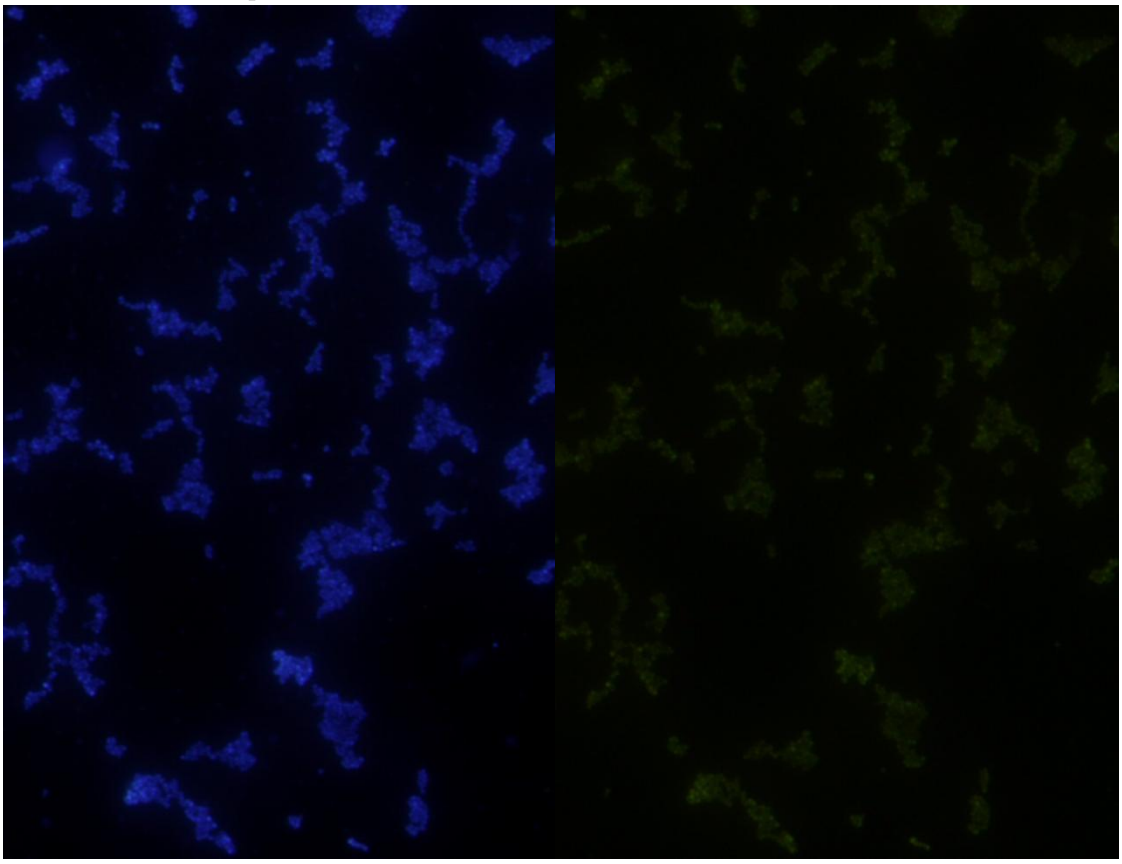

Peptostreptococcus anaerobius ATCC 27337

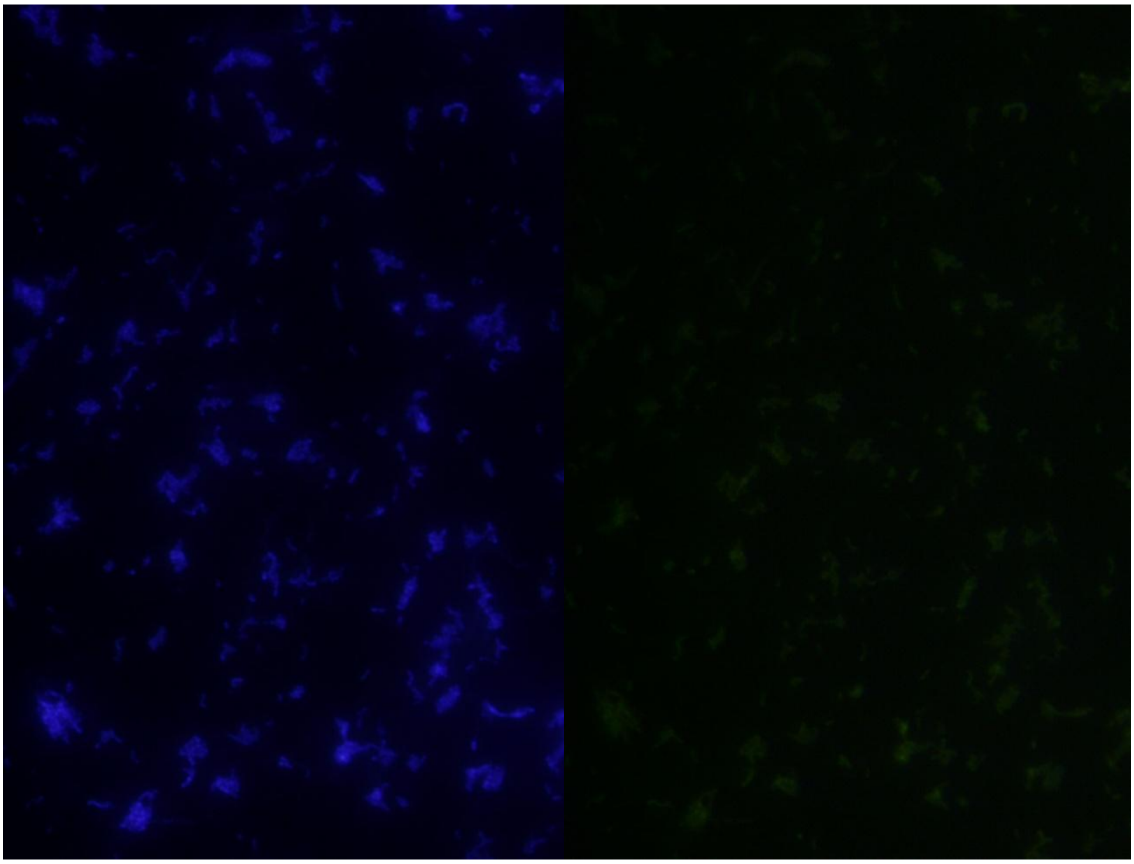

Cutibacterium acnes UM034

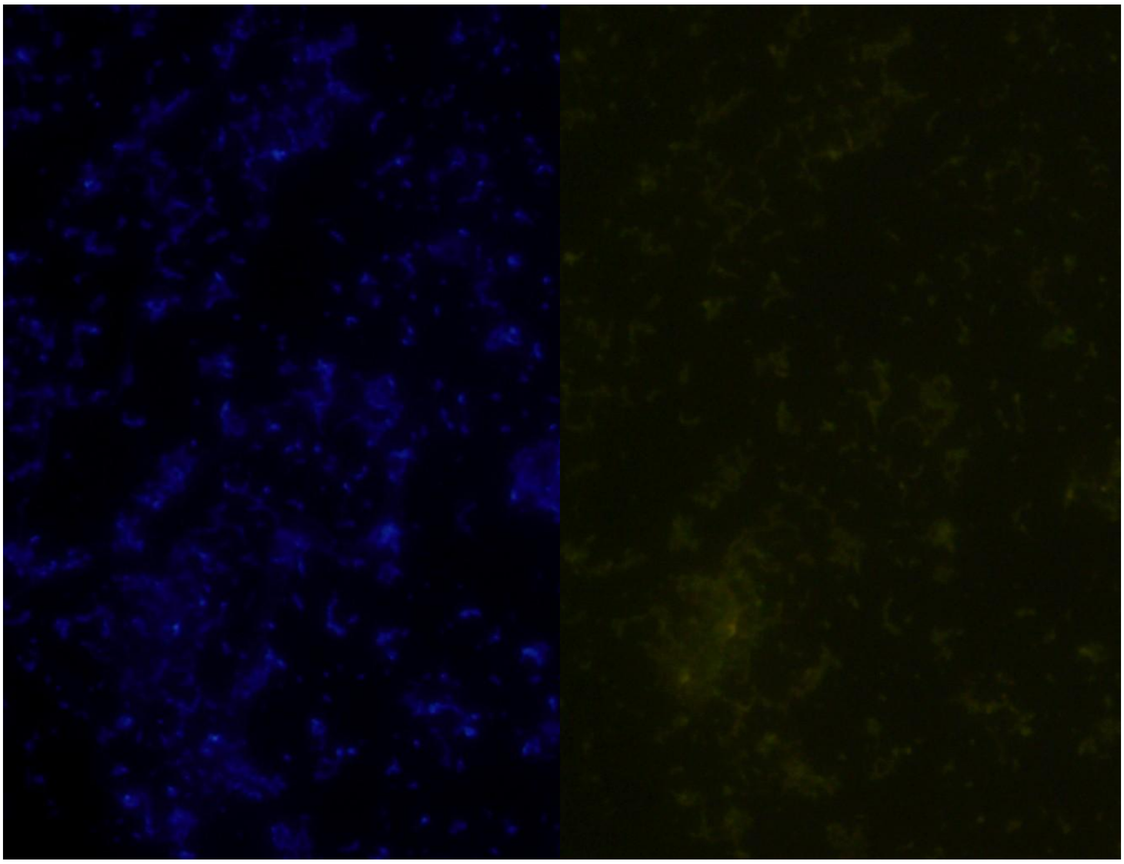

Staphylococcus simulans UM059

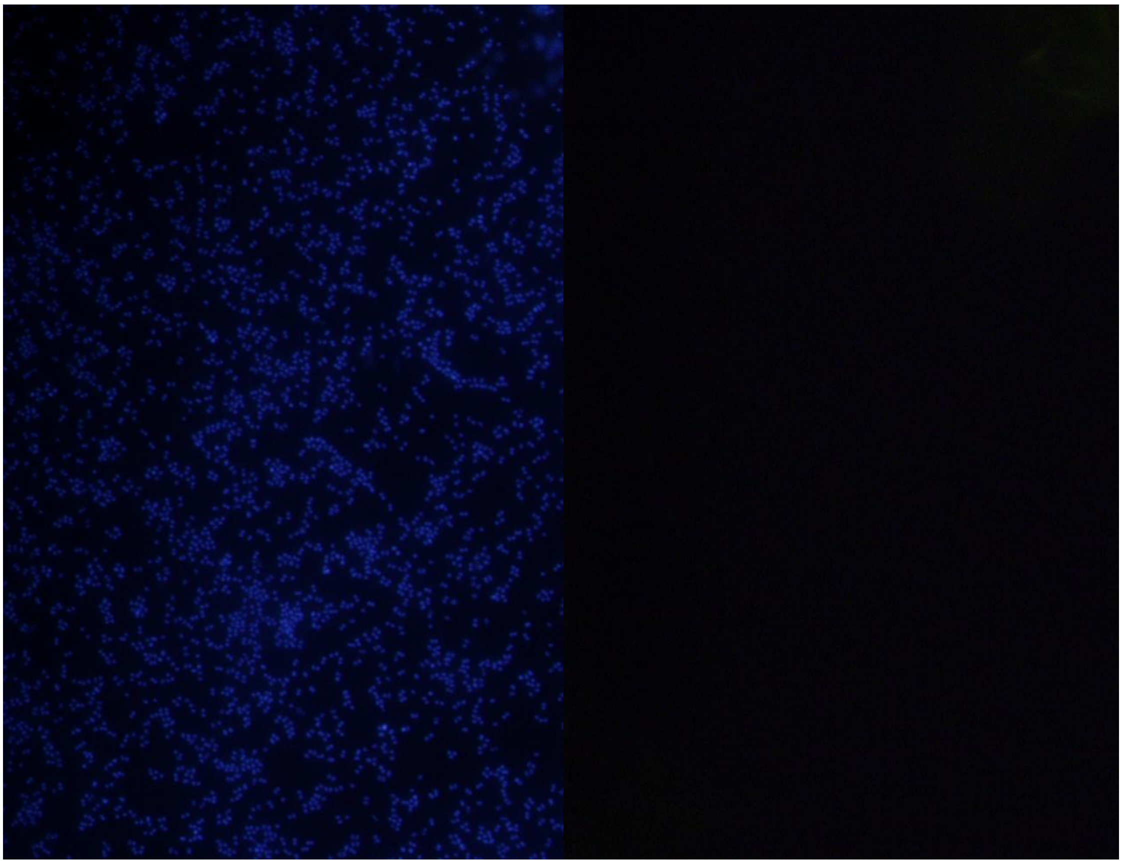

Supplement: Supplemental Information 3 [file peerj-14-20902-s003.pdf]
